# Ion Channels as Drug Targets in Central Nervous System Disorders

**DOI:** 10.2174/0929867311320100005

**Published:** 2013-04

**Authors:** A.M Waszkielewicz, A Gunia, N Szkaradek, K Słoczyńska, S Krupińska, H Marona

**Affiliations:** Department of Bioorganic Chemistry, Chair of Organic Chemistry, Faculty of Pharmacy, Jagiellonian University Medical College, 9 Medyczna Street, 30-688 Krakow, Poland

**Keywords:** ASIC, central nervous system, CNS, KCNQ, ion channels, NMDA, P2X, TRP.

## Abstract

Ion channel targeted drugs have always been related with either the central nervous system (CNS), the peripheral nervous system, or the cardiovascular system. Within the CNS, basic indications of drugs are: sleep disorders, anxiety, epilepsy, pain, etc. However, traditional channel blockers have multiple adverse events, mainly due to low specificity of mechanism of action. Lately, novel ion channel subtypes have been discovered, which gives premises to drug discovery process led towards specific channel subtypes. An example is Na^+^ channels, whose subtypes 1.3 and 1.7-1.9 are responsible for pain, and 1.1 and 1.2 – for epilepsy. Moreover, new drug candidates have been recognized. This review is focusing on ion channels subtypes, which play a significant role in current drug discovery and development process. The knowledge on channel subtypes has developed rapidly, giving new nomenclatures of ion channels. For example, Ca^2+^ channels are not any more divided to T, L, N, P/Q, and R, but they are described as Ca_v_1.1-Ca_v_3.3, with even newer nomenclature α1A-α1I and α1S. Moreover, new channels such as P2X1-P2X7, as well as TRPA1-TRPV1 have been discovered, giving premises for new types of analgesic drugs.

## I INTRODUCTION

Ion channels have been always related with drug discovery process. Their types, primarily recognized as Na^+^, K^+^, Ca^2+^, Cl^-^, have been basically associated with neuronal processes. Therefore, drugs targeted to them influence all organs or systems related with neuronal activity: the central nervous system (CNS), the peripheral nervous system, and the cardiovascular system. Within the CNS, basic indications of drugs are: sleep disorders, anxiety, epilepsy, pain, etc.

Due to the fact that many CNS diseases are related to variable etiology, many drugs have been developed by *in vivo* screening, where pharmacological tests performed on animals resemble a state of disease in a human brain. Therefore, the drugs reveal selectivity towards certain channel types, eg. lamotrigine, a known and quite modern anticonvulsant, acts mostly via Na^+^ and Ca^2+^ channels. 

Lately, novel ion channel subtypes have been discovered, which give premises to drug discovery process which lead towards specific channel subtypes. An example is Na^+^ channels, whose subtypes 1.3 and 1.7-1.9 are responsible for pain, and 1.1 and 1.2 – for epilepsy. Currently hardly any drug is specific to a single channel, which contributes to drug toxicity. However, new drug candidates have been recognized.

This review is focusing on ion channels subtypes, which play a significant role in current drug discovery and development process. Nowadays, there are no specific drugs targeted to a single channel subtype. Moreover, the knowledge on channel subtypes has developed rapidly, giving new nomenclatures of ion channels. For example, Ca^2+^ channels are not any more divided into T, L, N, P/Q, and R, but they are described as Ca_v_1.1-Ca_v_3.3, with even newer nomenclature α1A-α1I and α1S. Moreover, new channels such as P2X1-P2X7, as well as TRPA1-TRPV1 have been discovered, giving premises for new types of analgesic drugs. 

The review has been divided by channels families, subfamilies, and drugs in various stages of development. Structural diversity of channel subtypes has been shown. The biological activity of drugs has been described and structure-activity relationship, where possible, has been commented.

## II ION CHANNELS

### 1 Voltage-gated Sodium Channels

The voltage-gated sodium channels (VGSCs) are heteromeric transmembrane proteins which open in response to alteration in membrane potential to provide selective permeability for sodium ions [1].

Volted-gated sodium channels as drug targets in CNS disorders were recently deeply reviewed by Mantegazza *et al.* [2], Chahine *et al.* [3] and Tarnawa *et al.* [4]. In the current review we would like to summarize up to date information regarding their use in CNS disorders.

**Fig. (2):**
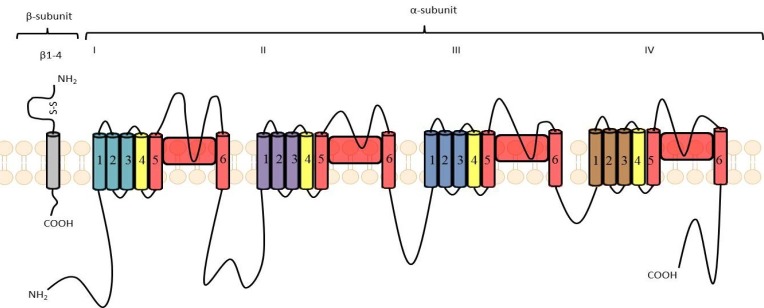
Schematic arrangement of the α-and β-subunits of the VGSC. The pore is colored in red, the voltage sensors are yellow [according to: 2, 5].

Sodium channels are built by several subunits. Subunit α forms a Na^+^ selective pore. It has molecular mass of 260 kDa. It consists of four homologous domains (I-IV or D1-D4), of which each contains six α-helical transmembrane segments (S1-S6) and one non-helical relatively short reentrant segment (SS1/SS2), known also as the P-segment, located between S5 and S6. All segments and domains are connected by internal or external polypeptide loops (Fig. **2**). The S4 segments are positively charged due to presence of positively charged amino acid residues and their role is to initiate the voltage-dependent activation of sodium channels by moving outward while influenced by the electric field. Therefore, S4 segments serve as voltage sensors. The short intracellular loop connecting domains III and IV occlude the cytoplasmic end of the pore when channel inactivates. The membrane reentrant loops between S5 and S6, which are the part of P-segment form the ion selectivity filter and ion pathway as well as the outer region of the pore. Sodium channels possess also one or more β-subunits of about 35 kDa [5, 6]. The role of β-subunits is influencing the properties of α-subunits including modulation of sodium currents. Moreover, they function as cell adhesion molecules and play role in aggregation, migration as well as cell surface expression. β-subunits typically possess a large extracellular immunoglobulin-like N-terminal domain, a single transmembrane region and intracellular C-terminal region. α- and β-subunits are associated non-covalently (α with β1 or β3) or covalently, *e.g*. by means of disulfide bond (α with β2 or β4) [6, 7]. 

**Fig. (3):**
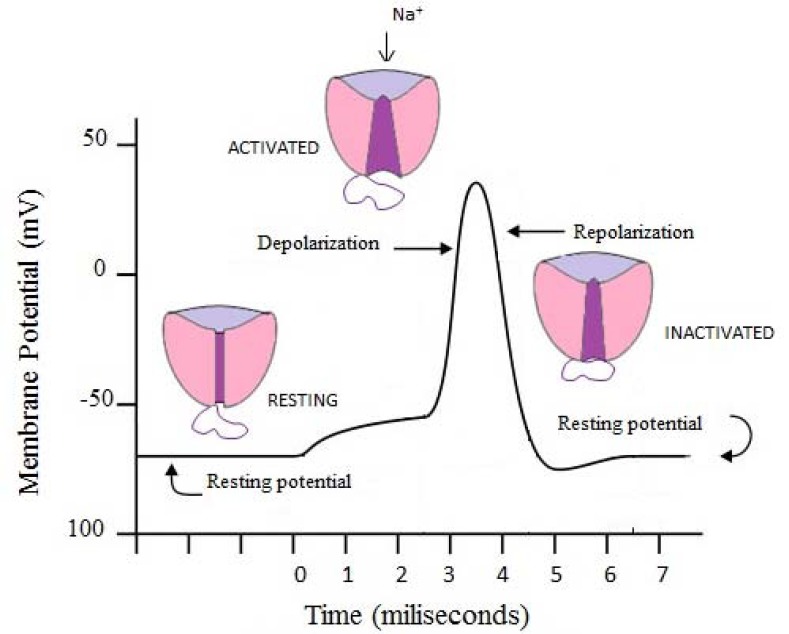
Action potential of neuronal membrane in different functional states of VGSC.

There are at least three functional states of sodium channels (Fig. **3**). The “resting” (closed, activateable) state emerges from conformational change that requires repolarization of the membrane (membrane potentials below -60 mV). In that state the channels are ready to open. In response to membrane depolarization they become “open” (or “activated”) and allow rapid influx of sodium ions. Then they are converted to “inactivated”, when the channels undergo conformational changes in which isoleucine, phenylalanine, and methionine between domains III and IV play an important role. When inactivation takes a time of around 1 ms it is called “fast-inactivation” and channels shift into a fast-inactivated state. On the other hand, some channels undergo slow-inactivation, which takes considerably longer time (seconds to minutes). As a result “slow-inactivation” state, a fourth possible functional state is created [1, 6]. 

Moreover, functional changes and increased permeability of Na^+^ ions result in generation of sodium currents, which can be measured. Those currents are responsible for the upstroke of action potential. Many neurons possess two types of sodium currents: transient and persistent. Transient Na^+^ current (*I*_NaT_) is related with opening of the channel when sodium ions passively move through the channel on the basis of its electrochemical gradient [8]. On the other hand, persistent Na^+^ current (*I_NaP_)* is a small slowly inactivating sodium current with relatively long kinetics of inactivation (tens of seconds), which appears when inactivation of channels is incomplete [2]. It activates as potentials close to or slightly more negative than resting membrane potential and hardly inactivates. Its amplitude is relatively small, it constitutes about 1% of the peak amplitude of the transient sodium current [8, 9].

First classification of sodium channels divided them into two groups, either sensitive or non-sensitive to the puffer fish toxin, tetrodotoxin (TTX). The existing names of voltage-gated sodium channels are derived from the kind of α-subunit that have been cloned. Channels named as Na_V_1.1 to Na_V_1.9 refer to proteins and differ in functional form of α-subunit. Moreover, a unique α-subunit known as Na_X_ has been also recognized. The subunit is lacking amino acids needed for proper voltage gating and it has been shown to be gated by extracellular sodium concentration [6]. TTX-resistance is characteristic for Na_V_1.5, Na_V_1.8 and Na_V_1.9 (blockade in micromolar concentration), while Na_V_1.1, Na_V_1.4, Na_V_1.6 and Na_v_1.7 are sensitive to nanomolar concentrations of TTX [6]. Expression of different subtypes shows cell and tissue specificity (Table **1**).

Names SCN1A-SCN5A and SCN8A-SCN11A refer to genes which code proteins building different α-subunits of sodium channels, and SCN6A/SCN7A codifies Na_x _subunit [2]. SCN1B-SCN4B are genes responsible for encoding of β-subunit, β1-β4, respectively [7]. SCN1B is expressed as two splice variants, β1 and β1B, both characteristic for human brain and heart [10]. Four sodium channel genes are primarily expressed in CNS: SCN1A, SCN2A, SCN3A, and SCN8A [11]. 

### Epilepsy

Epilepsy is one of the most common neurological disorder. In general, it is a tendency to the occurrence of unprovoked epileptic seizures which are a result of synchronous discharges of large number of neurons [19, 20]. It is now known that abnormal expression or function of VGSCs may be involved in the pathophysiology of both acquired and inherited epilepsy [2]. Great evidence of the role of some VGSCs in epileptogenesis emerges from the identification of several mutations in VGSCs genes leading to inherited epileptic syndromes. Some examples are listed in (Table **2**).

The disease in also thought to be associated with significantly increased persistent Na^+^ current, 2-5 times larger than under physiological conditions, which is observed in models of temporal lobe epilepsy and in neurons obtained from the resected temporal lobe of epileptic patients [2]. This current may result in enhancing of synaptic potentials, generation of subthreshold oscillations, facilitation of repetitive firing, and prolongation of depolarized potentials [23]. Persistent sodium current is especially interested when observed in neocortical pyramidal neurons [24].

**Fig. (4):**
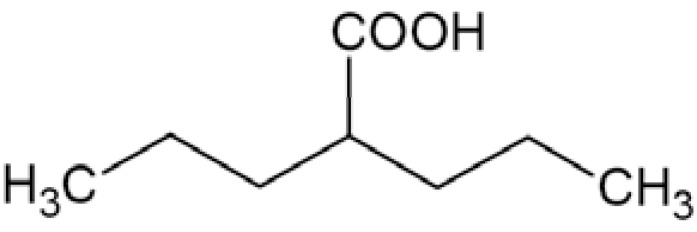
Chemical structure of valproic acid.

For any compound, including sodium channel blockers, anticonvulsant activity is not possible without penetration of blood-brain barrier, because chemical molecule must reach its target Na_V_1.1, Na_V_1.2, Na_V_1.3, and/or Na_V_1.6 channels localized in brain [12, 11]. Moreover, state-dependent affinity to open and/or inactivated channels is beneficial with respect to pathomechanism of epilepsy. Thus, anticonvulsants possess little effect on normal brain activity but affect pathological discharges during seizures. Inhibition of open and/or inactivated channels results in strong delaying the recovery from that state and reduction of sodium conductance [25]. Sodium channel blockers used as antiepileptic drugs do not show significant species or subtypes differences in their potency, which may be result of highly conserved nature of the binding site of those drugs [12]. Phenytoin and carbamazepine belong to antiepileptic drugs (AEDs) which exert their action mainly through inhibition of Na^+^ current. Their clinical efficacy in partial and generalized tonic-clonic seizures and lack of activity against absence seizures stay in agreement with their activity profile in animal seizure models. They are effective in maximal electroshock seizure (MES) test which is thought to be a predictive model for generalized tonic-clonic seizures, but they are not active in subcutaneous pentetrazole test (scMet) which is considered as predictive for drug’s activity against nonconvulsive seizures [22, 26]. On the other hand, many currently used AEDs have a mixed mechanism of action and sodium channels inhibition or modulation only accompanies influencing additional targets in brain. Those drugs are clinically effective against different types of seizure. There are still many concerns about contribution of VGSCs inhibition to their anticonvulsant efficacy [26]. Valproic acid (2-propylpentanoic acid) (Fig. **(4**) may serve as an example. It is a one of the most widely used AEDs in the treatment of generalized and partial seizures both in adults and children [27]. Several studies reported its possibility of reducing sodium currents in neocortical rat neurons (at concentration 0.2-2mM) [28], and especially persistent Na^+^ current (with high potency IC_50_ of 13.87±0.36 µM) [24] as well as in recombinant human Na_V_1.2 channels (IC_50_ 514 µM) [12]. The proposed mechanism of VGSCs alteration is that valproate, being a fatty acid, may modulate the channels by influencing the biophysical properties of the channel’s membrane but it does not explain the whole activity [27, 29].

### Neuronal Cell Damage (Neurodegeneration)

**Fig. (5):**
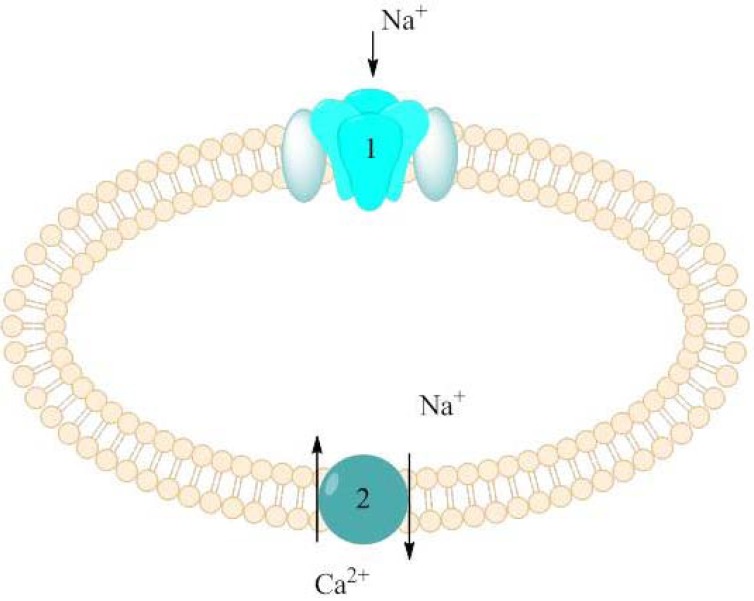
Raised Ca^2+^ concentration in cell as a consequence of increased Na^+^ influx.

Neuronal cell damage may be caused among others by hypoxia which appears in case of decreased or interrupted oxygen delivery. Persistent sodium current was identified as early and fundamental event in hypoxia. Other mechanisms involved in central axons injury are Na^+^-K^+^ ATPase failure and K^+^ efflux. As a result, increased concentration of sodium ions as well as depolarization of cell membrane were observed, among other in rat hippocampal neurons [30, 31]. Raised level of Na^+^ concentration triggers action of the Na^+^/Ca^2+^ exchanger (Fig. **5**). As a consequence Ca^2+^ concentration significantly increases generating Ca-dependent injury mechanism. Thus, drugs which inhibit persistent sodium current might reduce cell damage in CNS neurons during ischaemia or hypoxia, be means of secondary decreasing of Ca^2+^ concentration in cells [9]. 

Na_V_1.6 were found to produce larger sodium persistent current than other types of sodium channels which may be responsible for increasing action of the Na^+^/Ca^2+^ exchanger. As a result, injury of demyelinated axons in spinal cord is likely to occur [17]. Persistent sodium current may also constitute an important factor contributing to neuronal damage in Amyotrophic Lateral Sclerosis (ALS) [32]. It is worth to mention that multiple sclerosis (MS), a prototypical white matter disorder is not only neuroinflammatory condition but may be also caused by mitochondrial dysfunction and its molecular mechanism of tissue damage is similar to that of hypoxic CNS injury. It has been proposed that neuroprotecting agents designed for ischemia may be effective in MS as adjuvant therapeutics [33]. Increased influx of sodium ions into the cells has been also postulated as a key early event in the pathogenesis of secondary traumatic central nervous system injury. Some VGSCs blockers like phenytoin, or riluzole showed neuroprotective activity in many experimental spinal cord injury studies in aspects of motor, neurobehavioral and histopathological recovery [34]. 

### Migraine

Migraine is a common episodic CNS disorder, which is characterized by recurrent attacks of disabling headaches and associated symptoms [35]. Several sodium channels blockers have been implicated in the therapy of migraine, however, their precise mechanism of action in that condition is not fully understood [2]. It is generally accepted that inhibition of sodium currents can decrease cortical hyperexcitability predisposing to migraine. Some hypotheses say also that VGSCs blockers may influence cortical spreading depression (CSD), defined as slowly propagating wave of neuronal and glial depolarization. There is growing evidence that migraine aura and headache are triggered by CSD. Moreover, familial hemiplegic migraine, an autosomal dominant migraine syndrome, may be caused among others by mutation in SCN1A gene [36].

### Neuropathic Pain

Neuropathic pain is a kind of pain caused by functional abnormality of neurons, related with their damage [37]. Voltage-gated sodium channels located in peripheral sensory neurons play an important role in its pathophysiology because of their hyperexcitability and generation of spontaneous action potential firings. Blockade of VGSC contributes to analgesic activity [38]. Most beneficial in pharmacotherapy of neuropathic pain could be some selective Na_V_1.7, Na_V_1.8 or Na_V_1.9 channels blockers, till now not available at the pharmaceutical market. In that situation non-selective blockers are used which were initially evaluated in different disorders *e.g.* epilepsy [37]. Na_V_1.3 sodium channels have also been implicated in peripheral neuropathic pain. They are expressed at relatively high level in rat embryonic dorsal root ganglion (DRG) neurons but in adult rodent their expression in DRG is very limited [39]. However, after either chronic inflammation or nerve injury they are upregulated in second-order dorsal horn sensory neurons. These findings suggest that Na_V_1.3 might be related with central neuropathic pain [39, 40]. Thus not only Na_V_1.7, Na_V_1.8 or Na_V_1.9 blockers, but also Na_V_1.3 blockers may be used in the treatment of neuropathic pain. 

### Ataxia

Ataxia is another condition associated with sodium channels, and more specific with Na_V_1.6. The reason of that assumption rose form genetic studies which showed that mutations in mice Scn8a gene result in a variety of symptoms ranging from mild ataxia to dystonia, paralysis, and juvenile lethality [41]. Other research proved the role of their mutations in epileptic syndromes. They might serve as genetic modifiers of SMEI and GEFS+ [11]. Studies in human genome selected one mutation in the SCN8A gene expressed Na_V_1.6 channels which may be responsible for motor and cognitive deficits of variable expressivity. This heterozygous null mutation leads to cerebellar atrophy, ataxia and mental retardation, but an epileptic phenotype in humans was not seen [42]. Selective ligands for Na_V_1.6, if arise, may find a place among therapeutics for ataxia. 

### Sodium Channels/Sodium Currents Blockers

**Fig. (6):**
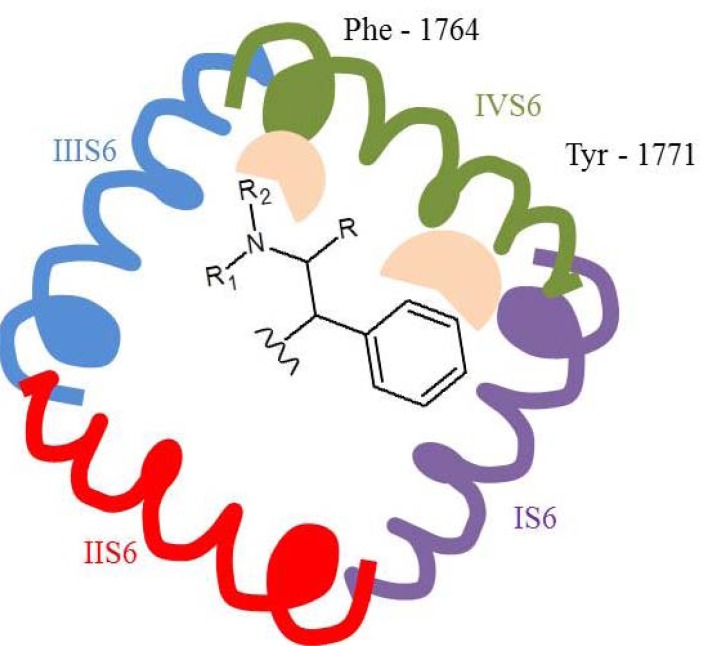
Simple presentation of binding sites of AEDs acting as sodium channels blockers in the Na_V_1.2 homology model [46]. Aromatic ring has an aromatic-aromatic interaction with Tyr-1771, and amide, imide or amine moiety interacts with the aromatic ring of Phe-1764 by a low-energy amino-aromatic bond.

Sodium channel blockers were used in therapy even before their pharmacological targets had been identified or cloned. For example, the first local anaesthetic agent, cocaine, was introduced in 1884 for perioperative analgesia [6]. With the development of science, detailed information about structure of sodium channels has become available. At first, simple models were derived, showing how subunits are embedded in membrane (see (Fig. **2**)), then schematic pore-forming regions were created using known potassium channels as model channel [1]. Finally, crystal structure of *Arcobacter butzleri *voltage-gated sodium channel (Na_V_Ab) was obtained [43, 44]. Parallel with structural studies on sodium channels, functional assays have been developed. For that purpose, cloning of individual sodium channels, including human recombinant, both wild-type or even mutant, in different cell lines have been implicated. That enabled detailed studies on targeting a particular population of channels. Moreover, many technologies have been implicated to investigate biological properties of sodium channels blockers, *e.g. *radioligand binding assays, radioactive flux assays, fluorescence-based assays, usage of voltage-sensitive dyes [1]. Several binding sites on VGSCs have already been identified. Sodium channels are the molecular target of many neurotoxins which may serve as tools in studying the function and the structure of channels. For example, tetrodotoxin and saxitoxin bind to site 1 (domains I SS2-S6, II SS2-S6, III SS2-S6, and IV SS2-S6), batrachotixin, veratridine and aconitine to site 2 (domain IS6), α-scorpion toxins to site 3 (I S5-S6), β-scorpion toxins to site 4, DDT site 7, last but not least, common anticonvulsants and local anesthetics bind to site 9. Binding to specific site of the receptor is related with a defined physiologal effect, direct affecting of ion transport is characteristic for sites 1 and 9 and modification of gating process with sites 2-8 [5]. Phenytoin, carbamazepine and lamotrigine, despite different chemical structures, seem to bind to the same site of the receptor. The model of interaction is bimolecular which means one-to-one binding process. Moreover, anticonvulsant drug action’s site is thought to be located on the extracellular side of channel [45]. Molecular modeling has been also implicated in evaluation of binding of different sodium channels blockers but it uses homology models with other voltage-gated ion channels: calcium and potassium [25, 46]. Both molecular modeling and site-directed mutagenesis studies helped in indentifying residues which are important for binding to the channel (Fig. **(6**). A pharmacophore model of the AED binding to VGSCs suggests that presence of aromatic ring and polar amide, imide or amine group in special spatial arrangement is required [46]. In the field of sodium currents research invention of whole-cell patch clamp recordings enabled measurement of transient and persistent currents and thus activity of particular compounds on specific currents. 

Currently, sodium channels blockers serve as drugs in several common disorders. They are also extensively investigated, especially with respect to their selectivity. Many sodium channels blockers are state-dependent which means that they have preferential affinity for the open and/or inactivated channel’s state when compared to its resting state [6]. The same situation refers to both anticonvulsant drugs as well as local anesthetics. Although both groups significantly differ in chemical structure, they seem to bind to the same site of the channel’s pore with similar affinity showing different therapeutic effect [46]. VGSCs blockers act through different sites of α-subunit, no drugs are known to interact directly with β-subunit [1].

### Phenytoin (PHT)

Phenytoin (5,5-diphenylhydantoin), while tested in animals is active in MES test but not in scMet [26]. It is considered as a prototype sodium channel blocker among antiepileptic drugs. PHT preferentially binds to fast-inactivated rather than to the resting sodium channels [45], which has a beneficial role in inhibition of action potential spread. 

Preclinical evaluation showed also beneficial role of phenytoin in mice model of multiple sclerosis – experimental autoimmune encephalomyelitis (EAE). Administration of PHT provided improved clinical status, preservation of axons, enhanced action potential conduction and reduced immune infiltrates, so it definitely acted as neuroprotectant [47]. 

It is been shown that PHT binds to inactivated VGSCs with K_i_ 7 µM (tested in rat hippocampal neurons) [48] and 19 µM (tested in wild type sodium channels expressed in *Xenopus* oocyte) [49].

Molecular docking to Na_V_1.2 channel using homology model with crystal structures of potassium channels, showed that it might bind to the site located in domain IV-S6. Aromatic ring is responsible for aromatic-aromatic interaction with Tyr-1771, and polar amide or imide group interacts with the aromatic ring of Phe-1764 by a low-energy amino-aromatic hydrogen bond [46]. Several phenytoin derivatives have been synthesized and evaluated for affinity to Na^+^ channels [50] resulting in conclusion that the second aromatic ring in position 5 is not obligatory for VGSCs binding, it can be replaced with aliphatic pentyl, hexyl or heptyl chain. Moreover, phenytoin is effective in partial and generalized tonic-clonic seizures but not nonconvulsive seizures [26].

### Carbamazepine (CBZ)

**Fig. (8):**
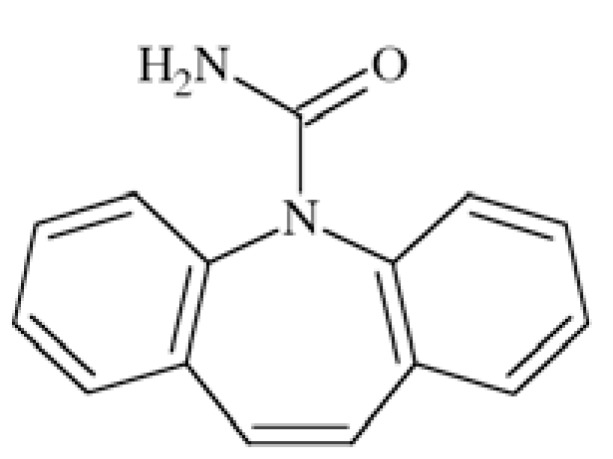
Chemical structure of carbamazpine.

Carbamazepine (5*H*-dibenzo[b,f]azepine)-5-carboxa mide) Fig. **(8**) is and old AED, developed in late 1950s - 1960s. While tested *in vivo *CBZ showed effectiveness in MES test (ED_50_s 7.81 mg/kg b.w. (body weight) mice, *i.p.* and 17 mg/kg b.w. mice *p.o*.) as well as amygdala-kindled rats model but it did not show activity in subcutaneous pentetrazole test [26, 37].

Although well established position in pharmaceutical market, its mechanism of action is not yet completely understood. One of the fact is that it inhibits VGSCs in a voltage-dependent and frequency-dependent manner [2]. It has higher affinity to fast-inactivated that to resting VGSC. CBZ also blocked persistent Na^+^ current in concentration dependent manner in Na_V_1.3 channels expressed in HEK293 cells with EC_50_ 16±4 µM and maximum block (E_max_) 46±4%. Inactivation of persistent sodium current seems to be parallel with inhibition of transient Na^+^ current [8]. Molecular modeling showed that the pharmacophore segments of CBZ and PHT are similar, and CBZ is also able to develop interaction with binding site in domain IV-S6 [46]. 

CBZ is used in the treatment of simple partial, complex partial and generalized tonic-clonic seizures but it is not effective against absence seizures [26, 37]. 

### Eslicarbazepine Acetate (ESL)

**Fig. (9):**
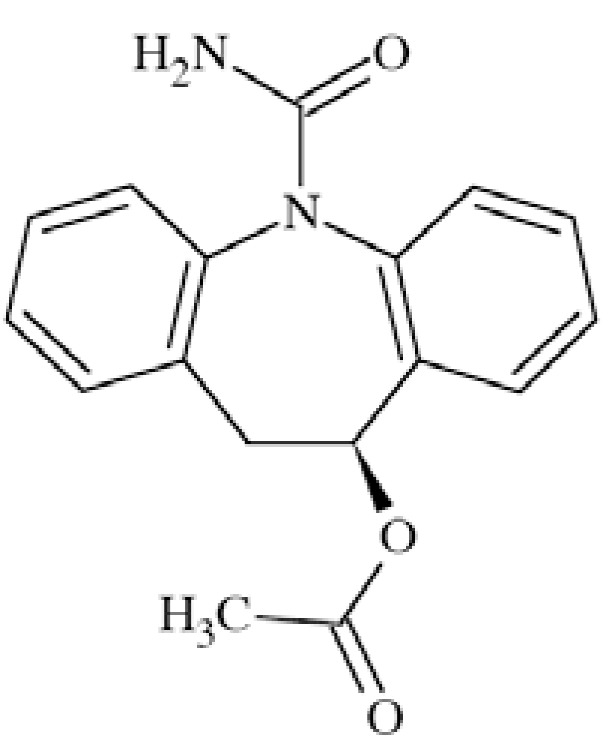
Chemical structure of eslicarbazepine acetate.

Eslicarbazepine acetate ((*S*)-(-)-10-acetoxy-10,11-dihyd ro-5*H*-dibenz[*b*,*f*]azepine-5-carboxamide, BIA 2-093) (Fig. **9**) is an AED second-generation to carbamazepine. It showed efficacy in several animal seizure models both electrically (MES) and chemically (pentetrazole, bicuculline, picrotoxin, 4-aminopyridine) evoked [51]. 

Although its precise mechanism of action is not yet explained, ESL proved to inhibit Na^+^ current in a voltage-dependent manner while tested in NIE-115 mouse neuroblastoma cells. It preferably binds to VGSCs in inactivated state what may result in limitation of repetitive firing and seizure spread. In tests performed in rat brain membranes ESL bonds in a competitive manner to neurotoxin site 2 of the channel, but not site 1, with IC_50_ of 222 µM (138-358) in displacing ^3^H-BTX [52]. ESL has been licensed in Europe (not in the USA) as adjunctive treatment for partial seizures with or without secondary generalization in adults [53]. 

### Lamotrigine (LTG) and Related Compounds

**Fig. (10):**
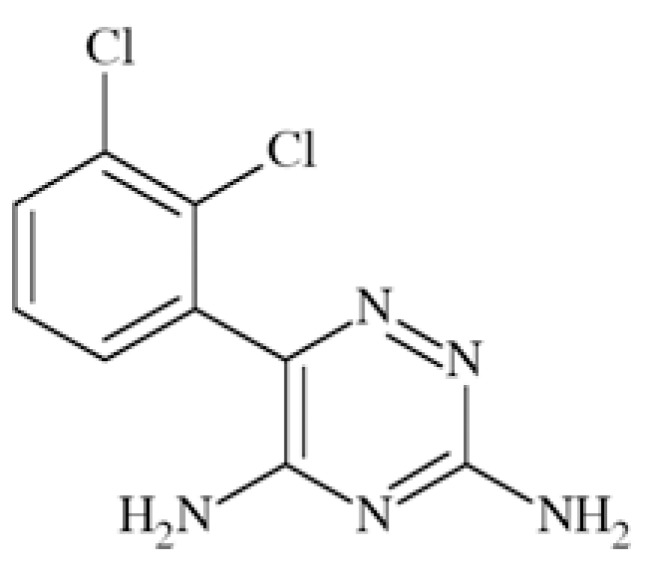
Chemical structure of lamotrigine.

Lamotrigine (6-(2,3-dichlorophenyl)-1,2,4-triazine-3,5-diamine) Fig. **(10**) proved efficacy in several animal seizure test: MES, 6Hz test, corneal kindled mice, hippocampal kindled rats, sound induced seizures. Its mechanism of action involves inhibition of sodium and calcium channels [22, 37]. 

Common pharmacophore concept for PTH enables to chose elements in the structure of LTG responsible for binding to the receptor site IV-S6. They are phenyl ring and amino group in position five of the triazine, which is moved from aromatic ring at the distance of three chemical bonds [46]. The binding affinity of LTG to rat brain Na_V_1.2 expressed in *Xenopus* oocytes in inactivated state was reported to be 31.9 µM [54] while compound’s affinity to the closed state of the channel was very low (IC_50_ 641 µM; inhibition of peak current at - 90 mV in human Na_V_1.2 α-subunit expressed in Chinese hamster ovary (CHO) cells) [55]. Many compounds with LTG related structure were synthesized. Examples of three, which had markedly different biological properties, helped in explanation of relationship of binding site of sodium channel with pharmacological effects. All compounds blocked sodium channel in a voltage-dependent manner but differed in their affinities for inactivated sodium channels (Table **3**). By means of assays with mutant sodium channels it was proved that all compounds bind to site located in S6 in domain IV, and more detailed to amino acid residues 1764, 1771 and in some extents also 1760. In fact, 1764 seems to be essential in voltage-dependent sodium channel blocking, whereas interaction with other residues is more variable [54]. 

### JZP-4

**Fig. (11):**
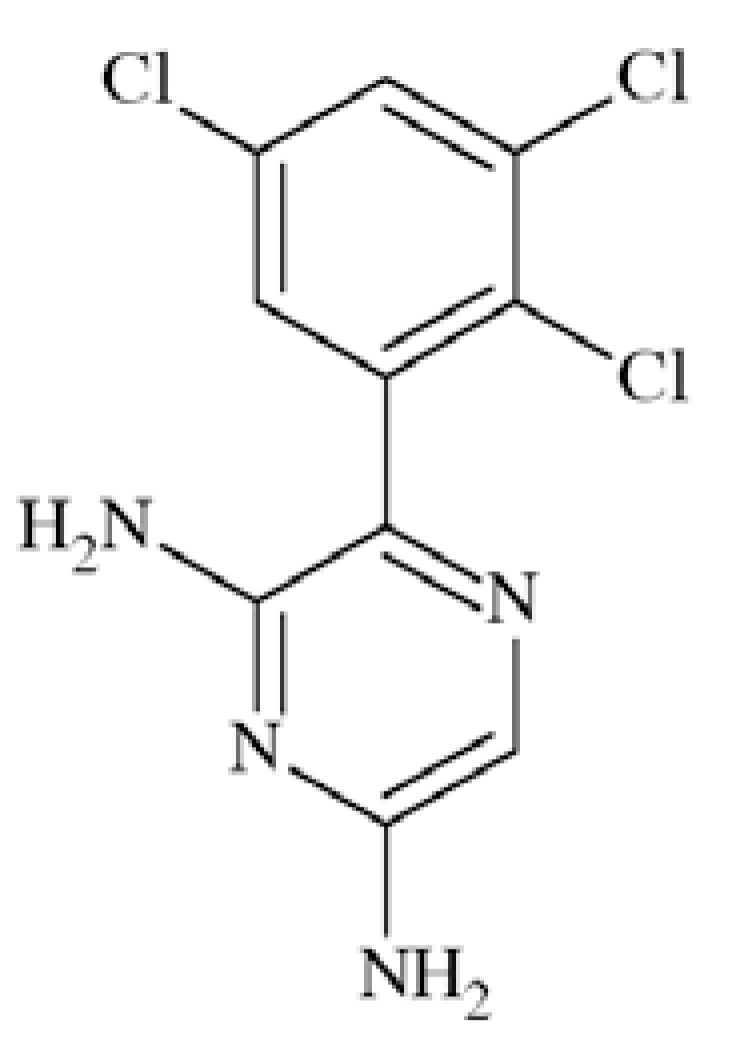
Chemical structure of JZP-4.

JZP-4 (3-(2,3,5-trichloro-phenyl)-pyrazine-2,6-diamine) Fig. **(11**) is a second generation drug to lamotrigine, with only slightly altered structure. It possesses broad spectrum of anticonvulsant activity in preclinical evaluation (MES, rat hippocampal and amygdala kindling, 6 Hz). It has inhibitory effect on both sodium and calcium channels [56].

Tests on human Na_V_1.2 sodium channels proved its voltage- and use-dependent inhibition, as well as possibility of hyperspolarizing shift in the inactivated state. IC_50_ values for JZP-4 were 165 and 6 µM at −90 and −60mV, respectively. While tested in Na_V_1.3 its IC_50_ were 333, 43 and 7 µM at −120, −90 and −70mV, respectively. In addition to the effect on Na_V_1.2A and 1.3 channels, JZP-4 also showed a weaker inhibition of NaV1.8/1.9 from rat dorsal root ganglia tissue. JZP-4 is in the Phase II of clinical evaluation in epilepsy. 

### Lacosamide (LCM)

**Fig. (12):**
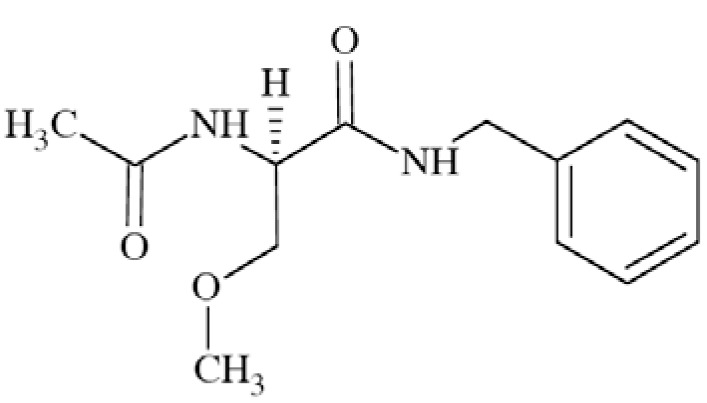
Chemical structure of lacosamide.

Lacosamide ((2*R*)-2-acetylamido-*N*-benzyl-3-methoxy propanamide; formerly harkoseride) Fig. **(12**) demonstrates broad anticonvulsant activity in several seizure models, like maximal electroshock seizure (MES) test, hippocampal kindling, audiogenic seizures, self-sustaining status epilepticus (SSSE), amygdala kindling, and 6 Hz test. Activity in 6-Hz test is unlike for other AEDs affecting sodium channels [57].

Lacosamide does not exhibit high-affinity for a range of receptors and ion channels. However, it showed weak affinity for site 2 of rat cortex VGSC (displacement of [^3^H]-batrachotixin – 25% of inhibition at 10 µM). After further test, it was proved that LCM’s anticonvulsant and analgesic effects are due to attenuation of Na^+^ currents. Lacosamide tested in recombinant human Na_V_1.3 and Na_V_1.7 sodium channels possessed higher affinity for inactivated channels than for resting but still less than carbamazepine. In contrary to other AEDs like carbamazepine, LCM probably affects sodium channel slow inactivation with no effect on fast inactivation. This is a unique mechanism of action which results in preferentially block the electrical activity of neurons that are chronically depolarized but not those possessing more normal resting potentials [15, 58]. 

LCM has been licensed for the treatment of partial-onset seizures. It has properties that may be also useful for a broad range of neuropathic pain patients *e.g*. in painful diabetic neuropathy [15, 57].

### Topiramate (TPM)

**Fig. (13):**
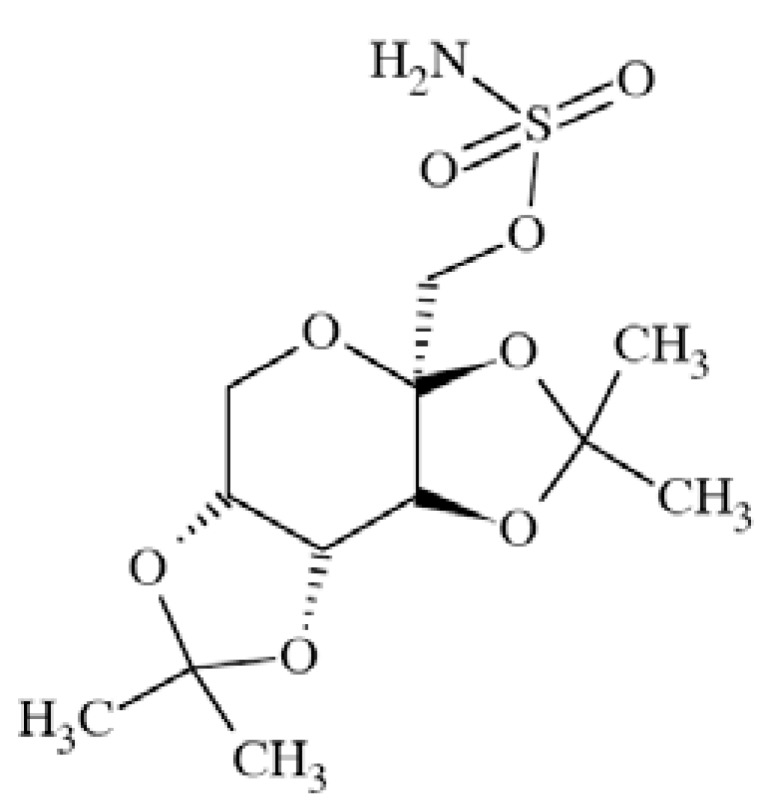
Chemical structure of topiramate.

Topiramate (2,3:4,5-bis-O-(1-methylethylidene)-β-*D*-fruc topyranosesulfamate) Fig. **(13**) exerts its antiepileptic activity by means of different mechanisms of action like VGSCs blockage, potentiation of GABAergic transmission, and AMPA receptor sites modulation. It shows relatively broad spectrum of anticonvulsant properties in animal studies like MES (mice and rats), genetically seizure-prone DBA/2 mice, amygdala kindled rats. Topiramate is inactive/weakly active in chemically-induced seizure models including pentetrazole, picrotoxin, bicuculline, strychnine [59]. 

It was proved that TPM blocks persistent Na^+^ current in Na_V_1.3 expressed in HEK293 cells with EC_50_ 61±37 nM and maximum block E_max_ 30±4%. At the same time, its ability to shift transient sodium current to negative potentials is much lower (ED_50_ 3.2 µM). This finding suggests that topiramate acts mainly by means of inhibition of persistent Na^+^ current on the contrary to popular AEDs like carbamazepine and phenytoin, which inhibit mainly transient Na^+^ current [8]. Its clinical indication is the management of refractory partial and secondarily generalized seizures [59]. At the doses of 25 up to 100 mg/day TMP was also approved for prophylaxis of migraine [60].

### Carisbamate

**Fig. (14):**
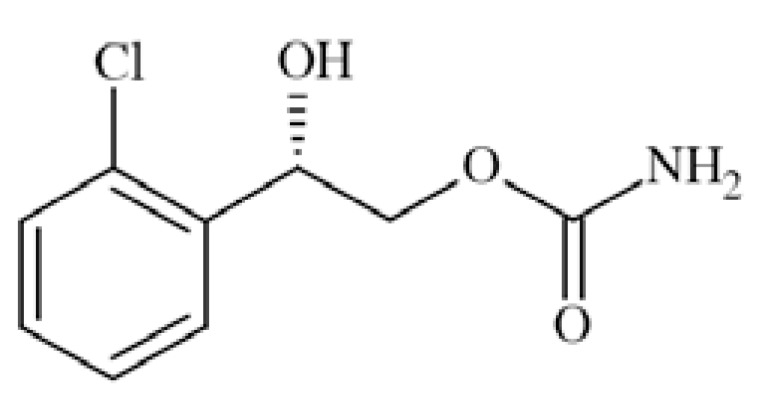
Chemical structure of carisbamate.

Carisbamate (*S*-2-O-carbamoyl-1-*o*-chlorophenyletanol, RWJ-333369) Fig. **(14**) showed a broad spectrum of activity in preclinical anticonvulsant evaluation, like in MES, scMET, bicuculine picrotoxin induced seizures, and audiogenic seizures. Moreover, it proved efficacy in amygdala kindled rats and lamotrigine-resistant kindled rats. Its proposed mechanism of action includes inhibition of VGSCs and modest inhibition of calcium channels [56, 22]. 

Carisbamate showed a concentration-, voltage- and use-dependent inhibition of rat Na_V_1.2 expressed in CHL1610 cells, with an IC_50_ value of 68 µM (at −67mV). In rat hippocampal neurons, carisbamate similarly blocked VGSCs, with an IC_50_ value of 89 µM (at −67mV), and inhibited repetitive firing of action potentials in a concentration-dependent manner (by 46% at 30 µM and 87% at 100 µM) [61].

### Rufinamide

**Fig. (15):**
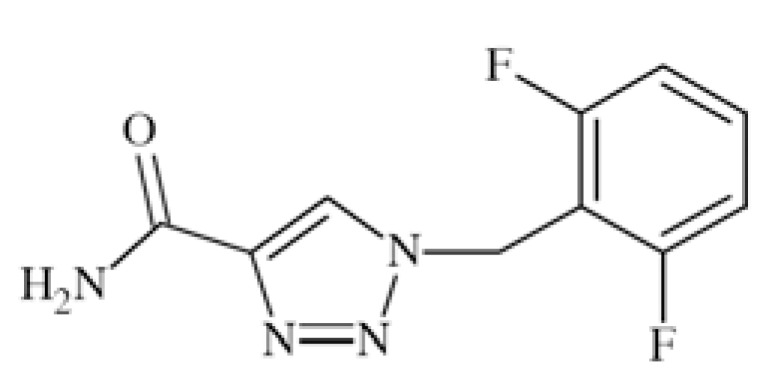
Chemical structure of rufinamide.

Rufinamide (1-(2,6-difluoro-phenyl)methyl-1H-1,2,3-tria zole-4-carboxamide, CGP 33101) (Fig. **15**) showed activity in MES and pentetrazole-induced test in rodents, as well as in bicuculline- and picrotoxin clonus in mice [53]. 

Modulation of sodium channels seems the main mechanism of action of that compound. At concentration 1 µM or higher it inhibited VGSCs recovery from inactivation in cortical neurons from immature rats. With EC_50_ of 3.8 µM it limited sustained repetitive firing of sodium dependent action potentials [62].

Rufinamide is available in Europe and the USA as efficacious and well-tolerated adjunctive treatment for patients with partial seizures and Lennox-Gastaut syndrome [53].

### Flunarizine (FLN)

**Fig. (16):**
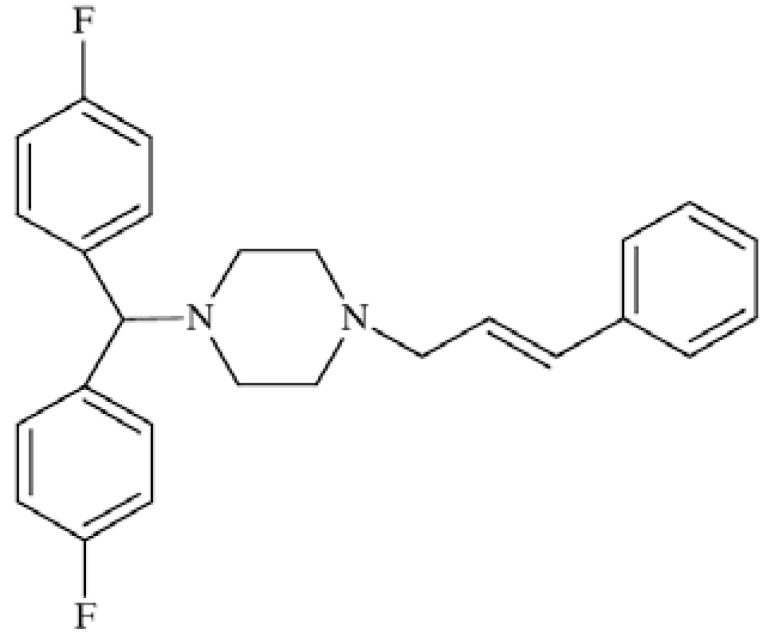
Chemical structure of flunarizine.

Flunarizine ((*E*)-1-[bis(4-fluorophenyl)methyl]-4-(3-phenyl-2-propenyl)piperazine) Fig. **(16**) acts by means of various mechanisms of action although not yet fully understood [63]. Recently it has been proved that the compound efficiently blocks cortical neuronal sodium current in a concentration- and use-dependent manner with IC_50_ value of 0.94 µM. Moreover, it delays the recovery from fast inactivated state INa. This finding suggests that inhibition of sodium current which can decrease cortical hyperexcitability may may be one of the brain targets of FLN [64]. It antagonizes T-type calcium channels, exhibiting affinity towards N-type channels of IC_50_=0.08 µM and towards L-type with IC_50_=0.31 µM [65]. Flunarizine has been successfully used for migraine prophylaxis [63].

### Riluzole

**Fig. (17):**
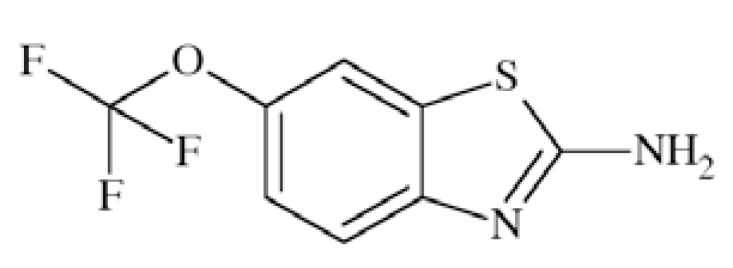
Chemical structure of riluzole.

Riluzole (2-amino-6-trifluoromethoxybenzothiazole, RP 54274) Fig. **(17**) is considered as neuroprotective agent with anticonvulsant properties. Its mechanism of action involves primary inhibition of VGSC but also reduction of glutamate release [32].

It inhibited persistent sodium current in dose-dependent manner with EC_50_ of 2 µM while tested in rat brain neurons [32]. Other studies conducted in bovine adrenal chromaffin cells showed blockade of Na^+^ channels by riluzole in concentration-dependent manner with IC_50_=5.3 μM and binding to the veratridine site 2 of channels. In the test using rat brain sodium channel α-subunits expressed in *Xenopus* oocytes the compounds blocked the close state of the channel with IC_50_ ranging from 30 to 90 µM, but inhibited the inactivated state of Na channels 150–300 times more effectively [66]. Riluzole in the only drug currently approved for the treatment of Amyotrophic Lateral Sclerosis (ALS) [32]. It is also a non-competitive NMDA antagonist. In a progressive stratial degeneration the compound was able to reduce motor symptoms associated with striatal lesions [67]. 

### Ranolazine

**Fig. (18):**
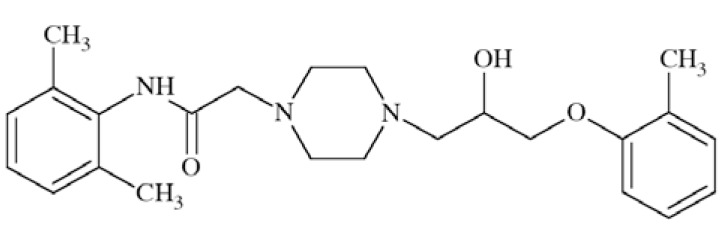
Chemical structure of ranolazine.

Ranolazine ((+)-*N*-(2,6-dimethylphenyl)-4-[2-hydroxy-3-(2-methoxyphenoxy)propyl]-1-piperazineacetamide) (Fig. **18**), a drug for the treatment of chronic angina [68], was previously evaluated as modulator of function of different sodium channels like Na_V_1.4, Na_V_1.5, Na_V_1.7, Na_V_1.8, and recently also Na_V_1.1. In the study, human wild-type and mutant Na_V_1.1 channels expressed heterologously in human tsA201 cells were used. The compound was able to block persistent current 16-fold more selective in comparison to tonic block of peak current and 3.6-fold more selective than use-dependent block of peak current. Interestingly, similar selectivity was observed for ranolazine in blockage of increased persistent current exhibited by Na_V_1.1 mutant channels. Ranalozine proved also ability to cross the blood-brain barrier while tested in rats, which together with inhibition of persistent current in mutant Na_V_1.1 channels give rise for possible providing a new useful therapeutic strategy for SCN1A-associated epilepsy and some migraine syndroms [69].

## 2 POTASSIUM CHANNELS

### Voltage-gated Potassium Channels

Voltage-gated potassium channels (VGKCs, K_V_) belong to broad family of potassium channels, including besides them Ca^2+^-activated (K_Ca_, ligand-gated), inward-rectifying (K_IR_), and two-pore (K_2P_) channels, which all contribute to the excitability of neurons, signaling in the nervous system as well as ion homeostasis. Potassium ion channels play critical role in repolarization of action potentials and regulation of firing frequency [70]. VGKCs were for the first time successfully cloned from *Drosophila melanogaster *cDNAs [71]. Tempel *et al.* [72] isolated mammalian cDNA of K_V_1.1 from mouse brain, later research has led to encoding of cDNA of other members of K_V_1 family, and finally to isolation of cDNAs of all currently known potassium channels [73]. In 2005 Gutman *et al.* [74] provided an opinion of International Union of Pharmacology concerning classification as well as deep review of pharmacology, regulation of expression, and disease association of voltage-gated potassium channels. In the current paper we would like to focus on VGKCs as targets in CNS disorders. 

**Fig. (19):**
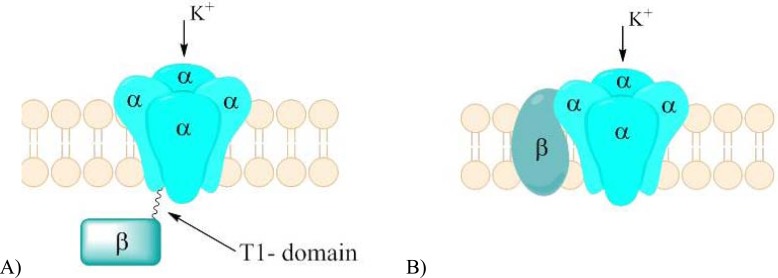
Schematic arrangement of the α-and β-subunits of K_V_ channel characteristic for K_V_1 and K_V_4 subfamily. There are differences in position of β-subunit.

Voltage-gated potassium channels contain four transmembrane pore-forming voltage-sensing α-subunits. Individual α-subunit of K_V_ channel consists of six segments (S1-S6). Four transmembrane S4 segments carry multiple positive charges and act as main voltage-sensing component, which responds to changes of membrane potential resulting in conformational alterations in the channel (voltage gating). Segments S5 and S6 from each of the four α-subunits form a pore, which is in fact an ionic conductance pathway. At the narrowest part of the pore, S5 and S6 connecting loop form a selectivity filter (P). Voltage-sensing segments surround the pore. Thus, the structure is similar to described above voltage-gated sodium channel. Although most of K_V_ channels are homotertamers (four α-subunits are the identical), they may also form heteroteramers, in which two or more distinct types of α-subunits occur (*e.g*. K_V_7.2/K_V_7.3). Those heteroteramers may possess different properties in comparison to homotetramers [70, 73]. Native neuronal potassium channels complexes contain both cytoplasmic and transmembrane auxiliary β-subunits Fig. **(19**). Several genes that code β-subunits were identified. Their alternative splicing can generate the number of functionally distinct isoforms. Moreover, although called auxiliary, they proved to be able to dramatically alter α-subunits expression and localization as well as functions by means of changing gating properties of channels [73]. 

3D reconstruction of the K_V_ α-subunit suggested that it is arranged in two distinct domains: the larger membrane-embedded and smaller cytoplasmic domain. Both domains are connected together by thin linker. The structure of the membrane-embedded domain is highly homologous among all K_V_ families [70]. On the other hand, the cytoplasmic domain differs both in structure and function between different K_V_ types. The very important part in cytoplasmic domain is so called tetramerization domain (T1), which is responsible for promoting the tetramerization of potassium channels subunits. T1 may be connected with cytoplasmic N-terminal fragment of the polypeptide chain, like in K_V_1- K_V_4 [75]. On the other hand K_V_7 and K_V_11 possess a unique tetramerization domain in the C-terminal region, which also form the binding sites for various ligands (calmodulin, phosphadiylinozytol-4,5-bisphosphate - PIP_2_, and cyclic nucleotide) that can modulate the channel’s function [76-78].

Currently K_V_ channels are divided based on the relative sequence homology into twelve subfamilies: K_V_1-K_V_12. K_V_1-K_V_4 channels were classified in one group, K_V_7 in another, K_V_5, K_V_6, K_V_8, K_V_9 in different ,and K_V_10-K_V_12 in the last. Among the subfamilies, K_V_5, K_V_6, K_V_8 and K_V_9 did not prove to yield functional expression in forming ion channels, that is why they are also called “electrically silent” α-subunit-like polypeptides. Undoubtedly they are able to modulate the expression and/or gating of channels formed from bona fide α-subunits which is similar to β-subunits functions. Names beginning with KCN refer to genes which code proteins of different α-subunits of potassium channels [73]. Early classification provided a division of voltage-gated potassium channels into three families: Shaker, KvLQT and ether-a-go-go. Ether-a-go-go family was further divided onto three subfamilies: eag, elk and erg. Those names still exist in the literature, they were created before providing the current classification and identification of broad range of currently known voltage-gated potassium channels (Table **4**). 

Voltage-gated potassium channels are present in whole organism, in different organs like: brain, spinal cord, skeletal and smooth muscle, heart, retina, lung, islets, spleen, thymus and many more (see Gutman *et al.* [74]). Expression of different subtypes throughout brain and spinal cord show specific patterns of subcellular localization, for example K_V_1 and K_V_3 are situated mostly in axons and in some dendrites (K_V_1.1, K_V_3.1, K_V_3.2), while K_V_2 and K_V_4 in neuronal somata and dendrites [73]. A great majority of known VGKCs are located in CNS but only few of them proved the ability to serve as drug targets in disorders like epilepsy (K_V_7.2-K_V_7.5), psychosis (K_V_7), ataxia type I (K_V_1.1), multiple sclerosis (K_V_1.3). There is also growing evidence that auto-antibodies reactive to voltage-gated potassium channels play a pathogenic role in a broad spectrum of CNS disorders. Raised level of voltage-gated potassium channels antibodies (VGKCs-Ab) have been reported in cases of limbic encephalitis, while the improvement in neuropsychological functioning in some patients correlated with the fall in antibodies. Thus, a term autoimmune limbic encephalitis (ALE) was introduced, for indistinguishable from other encephalopathies. Limbic encephalitis is commonly associated with syndromes like episodic memory impairment, disorientation, seizures, hallucinations, and sleep disturbance [79]. Raised level of VGKCs-Ab was also confirmed in patients with long standing drug resistant epilepsy [80] and in case of adult-onset drug refractory seizure disorder [81]. 

### Epilepsy

Voltage-gated potassium channels, because of their contribution to excitability of neurons, may serve as drug target in epilepsy. K^+^ currents occurring while opening the channels take part in reducing membrane excitability, thus VGKCs openers are of interest as antiepileptic drugs [82]. The especially interesting group of potassium channels while considering therapy of epilepsy is K_V_7 (KNCQ) family or sometimes even more specified K_V_7.2 and/or K_V_7.3. The members of KNCQ family, K_V_7.1 – K_V_7.5, are characteristic for different localizations in human organism, but only channels from K_V_7.2 to K_V_7.5 were found in brain (Table **5**). K_V_7 channels in physiological condition are activated by membrane depolarization at subthreshold membrane potentials (from about -60 mV) and produce a sustained outward current at membrane voltages negative to the firing threshold of action potentials, thus act as efficient inhibitors of neuronal excitability [83, 84]. 

K_V_7 channels were originally termed “M-channels” as all members of those family generated so called M-like currents when expressed in oocytes or cell lines. It was proved, that in native neurons, M-channels are composed of heteromeric K_V_7.2/K_V_7.3 subunits or homomeric K_V_7.2 subunits, probably with a contribution of K_V_7.5 [83]. 

Activity of K_V_7 channels may be modulated by means of several ligands of G-protein coupled receptors (GPCRs), which produce several signaling pathways (hydrolysis of PIP_2_; changes in local Ca^2+^ concentration; phosphorylation by protein kinases), which finally inhibit activity of potassium channels. Exact mechanism of that modulation is not completely understood. It was also proved that M-like currents are inhibited by activation of muscarinic receptors [84]. On the other hand, synthetic activators of K_V_7 seem to cause conformational changes leading to channel opening by direct binding to the protein [82].

Identification of KCNQ2 and KCNQ3 genes mutations as the molecular cause of benign familial neonatal seizures (BFNS; an autosomal dominant idiopathic epilepsy syndrome of newborns, characterized by unprovoked partial seizures typically beginning at the age of around three days), supported the position of those channels as drug targets in epilepsy. Those mutations are located in C-terminal cytoplasmic region, but missense mutations within the transmembrane have also been described [85, 86]. More evidence were found in animals. Watanabe *et al.* provided results from test in Kcnq2-knockout mice. Homozygotus mutant died within first postnatal day, while heterozygotus animals characterized normal behavior and morphology compared with wild-type mice. Further studies showed that Kcnq2+/- mice had much higher sensitivity to the chemoconvulsant – pentetrazole, [87]. Yang *et al.* gave evidence that mutation in Kcnq2 gene led to the decreased seizure threshold in electric-induced convulsions. Kncq2 mutation was also proved to be characteristic for *Szt*1 mice, which serve as genetic model of epilepsy [88]. Peters *et al.* showed that suppression of the M-currents in mice was the cause of spontaneous seizures and M-channels were found to be critical determinants of cellular and neuronal network excitability [89]. Pharmacological effectiveness in epilepsy was proved for K_V_7 channels activators (like retigabine described below). On the other hand, their blockers produced pro-epileptic side effects like tremors, thus their utility as therapeutics in other diseases is limited [83]. 

### Psychosis

K_V_7 (KCNQ) channels may represent a potential target for the treatment of psychosis, as some of them (KNCQ2 and KNCQ4) are expressed postsynaptically in dopaminergic neurons of mesolimbic and nigrostriatal pathways (*e.g*. in the ventral tegmental area). Opening of those channels is expected to decrease neuronal excitability, which can affect psychotic symptoms believed to be associated with an increased excitability of dopaminergic cells in the mesencephalon. The evidence that dopamine D2 receptors are functionally coupled with K_V_7 channels in dopamine neuron was proved by means of co-expressed channels and receptors in *Xenopus* oocytes and human neuroblastoma cells [90]. In other studies, the firing of dopaminergic neurons recorded in rat mesencephalic slices was significantly inhibited in a concentration-dependent manner by retigabine and that compound completely blocked the excitatory effect of dopamine D2 autoreceptor antagonists, suggesting that the modulation of dopaminergic activity by D2 autoreceptors would involve the activation of KCNQ2 and/or KCNQ4 channels [91]. More evidence came from *in vivo *electrophysiological studies conducted in anesthetized rats, in which K_V_7 channels activators (*e.g*. retigabine) suppressed burst firing activity in the ventral tegmental area, whereas XE-991, a selective KNCQ blocker, induced opposite effect. In the conditioned avoidance response paradigm one of *in vivo* rat models predictive for antipsychotic activity, retigabine was found to inhibit avoidance responses, moreover that effect was blocked by coadministration of XE-991. Furthermore, in phencyclidine (PCP)-sensitized rats, considered as a model for schizophrenia, retigabine was found to significantly inhibit the hyperlocomotor response to a phencyclidine challenge [92].

Above findings combined with immunocytochemistry test, which revealed that K_V_7.4 is the major K_V_7 channel type expressed in dopaminergic neurons [91] support for the hypothesis that those channels may serve as drug target in psychosis and that their activators could become a new class of antipsychotic drugs.

### Episodic Ataxia

Episodic ataxia type I (EA1) belongs to paroxysmal movement disorders. It is an autosomal-dominant disorder, characterized by intermittent cerebellar dysfunction with otherwise essentially normal brain function [93]. It was identified as a channelopathy connected with mutations in K_V_1.1 gene (KCNA1 in chromosome 12p13) [94]. A common clinical features associated with EA1 are: from CNS - occurrence of seizures, from peripheral nerves – neuromyotonia, but no connection with headache during acute episode was proved [95]. On the other hand, episodic ataxia type II is caused most often by the mutation in calcium channel gene CACNA1A, but potassium channel blocker – 4-amino pyridine, is one of the efficient treatment option [96]. Currently no VGKCs ligand is used in the therapy of EA1.

### Multiple Sclerosis

Multiple sclerosis (MS) is a disease of central nervous system, of which myelin damage is one of the pathological issues. There are various theories about the etiology of MS including immunologic, environmental, genetic, and infectious factors [97]. Myelin damage has been shown to cause abnormal K^+^ currents, thus potassium channels are believed to play an important role in multiple sclerosis, as they are responsible for conductance failure in that condition. Potassium channels blockade reduced this ionic leakage and improved conductance. Moreover, some VGKCs blockers were successfully used in many *in vitro* studies and animal models of neurodegenerative disorders as well as in clinical trials. Voltage-gated sodium channels, and especially K_V_1.3, were connected with several autoimmune diseases including MS. K_V_1.3 are expressed among others in immune system cells and organs like thymus, spleen, macrophages, lymphocytes. Blockage of those receptors localized in T-lymphocytes proved to be an effective approach in managing autoimmune disorders [98]. 

### Voltage-gated Potassium Channels Modulators

Voltage-gated potassium channels constitute a wide and hererogenous group thus no common binding pattern could not be determined. Modulators of different type potassium channels were recently reviewed by Wulff and Zhorov [99]. Here we would like to focus on selected VGKCs ligands and their utility in CNS disorders. 

### Retigabine (RTG; Ezogabine)

**Fig. (20):**
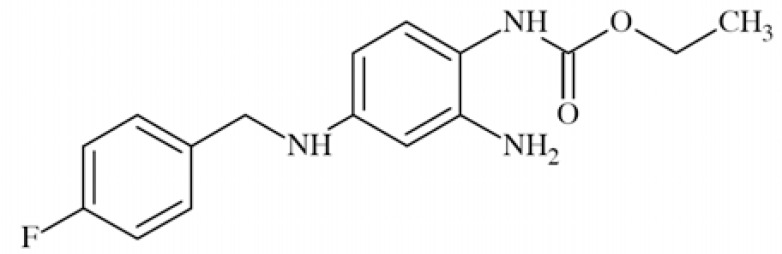
Chemical structure of retigabine.

Retigabine (*N*-[2-amino-4-(4-fluorobenzylamino)-phenyl]carbamic acid ethyl ester) Fig. **(20**) represents a unique mechanism of action among AEDs which is enhancing of the activity of K_V_7 channels in central nervous system resulting in reduction of neuronal excitability. The other mechanism constitutes enhancing of γ-aminobutyric acid transmission but only at supra-therapeutic concentration (while tested *in vitro*) [100]. The compound showed broad anticonvulsant activity in several animal seizure models: MES, scMet, 6-Hz test, hippocampal-kindled rats, amygdala-kindled rats ,and many more, including some genetic animal models of epilepsy [100]. Moreover, it had antipsychotic effect in an animal model of schizophrenia and mania [92]. It also showed activity in rat models of neuropathic pain [37].

Retigabine is thought to bind selectively to K_V_7.2 – K_V_7.5 channels, shifting the current–voltage curve to the left, which results that the channels open at more hyperpolarized membrane potentials [101]. The lack of RTG effect on K_V_7.1 may be due to inefficient binding, which, however, has not been proved [82]. It increased the rate of channels activation while tested in *Xenopus* oocytes expressing KCNQ2 [102]. Retigabine, at concentration 0.1 – 10 µM, affected CHO cells expressing K_V_7.2/K_V_7.3 heteromeric channel (CHO-KNCQ2/Q3) resulting in increasing M-like potassium currents and hyperpolarization of the cell membrane. Moreover, it shifted the voltage dependence of channel activation with an EC_50_ value of 1.6±0.3 µM [103]. The compound also significantly influenced properties of *Xenopus* oocytes membrane expressing recombinant human K_V_7.2/K_V_7.3, as well as channel itself. At the concentration of 10 µM RTG shifted both the activation threshold and voltage for half-activation by approximately 20 mV in the hyperpolarizing direction. It also affected kinetics of the channels, causing increased rate of activation and slowing deactivation. Membrane potential recordings showed that the compound caused a concentration-dependent hyperpolarization of the oocyte (IC_50_ of 5.2 µM) [102]. 

The prove that retigabine acts through activation of K_V_7 channels came also from *in vivo* animal studies. In two of them anticonvulsant efficacy was reduced by the selective K_V_7 blocker, XE-991.That happened in MES test in mice and in the rapid kindling model in rats [100]. Other test were performed in the *Szt*1 mouse model, in which functionality of the K_V_7.2 channel is impaired, because of C-terminal deletion in the Kcnq2 gene. Research showed that anticonvulsant potential of retigabine was then reduced [104]. It is worth to mention, that neuroprotective effect of RTG proved in some *in vitro* studies was not mediated by K_V_7.2 [100]. 

Systematic mutagenesis studies identified segment S5 as a binding site for retigabine, and more detailed a crucial tryptofan residue, which is specific for K_V_7.2 – K_V_7.5, while in K_V_7.1 there is leucine in corresponding position. Additional residues have also been found as affected by retigabine, like glycine residue in S6 [82]. 

Retigabine has recently been approved as adjunctive therapy in adults with partial-onset seizures [100]. 

### ICA-27243

**Fig. (21):**
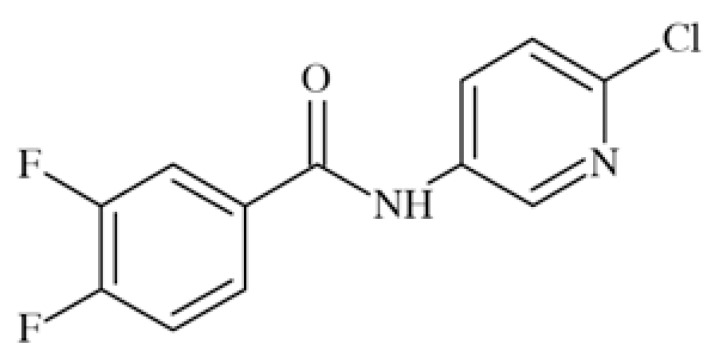
Chemical structure of ICA-27243.

ICA-27243 (*N*-(6-chloro-pyridin-3-yl)-3,4-difluoro-ben zamide) Fig. **(21**), considered as a second-generation structure to retigabine, is a selective activator of K_V_7.2/7.3. It showed in *in vivo* animal tests a broad spectrum of anticonvulsant activity including the MES, scPTZ, 6 Hz and kindling models [22].

ICA-27243 is a potent and selective activator of KV7.2/7.3 showing EC_50_=0.20±0.03 µM, determined in 86RB^+^-efflux assay conducted on recombinant human channels expressed in CHO cells. It the same test, EC_50_ for activation of K_V_7.4 was 7.1±0.1 µM and for K_V_7.3/7.5 was not able to be determined but estimated at about 10 µM. ICA-27243 shifted the voltage-dependent activation of K_V_7.2/7.3 to more hyperpolarized potentials in concentration-depen dent manner. The halfmaximal shift in the midpoint of the activation curve (Δ*V*_1/2_) was observed at 4.8±1.6 µM. The compound had no effect on GABA_A_-activated chloride channels, Na_V_1.2, or Ca_V_ channels.

Preclinical tests with ICA-27243 provided the evidence that K_V_7.2/7.3 activation alone is sufficient for broad-spectrum anticonvulsant activity in rodents. The finding that ICA-27243 can discriminate among different K_V_7 subtypes suggests that it may interact at a novel binding site on K_V_7.2 and/or K_V_7.3 channels that is not present in other K_V_7 channels. The binding site has anyway not yet been identified [105, 106].

### 4-Aminopyridine

**Fig. (22):**
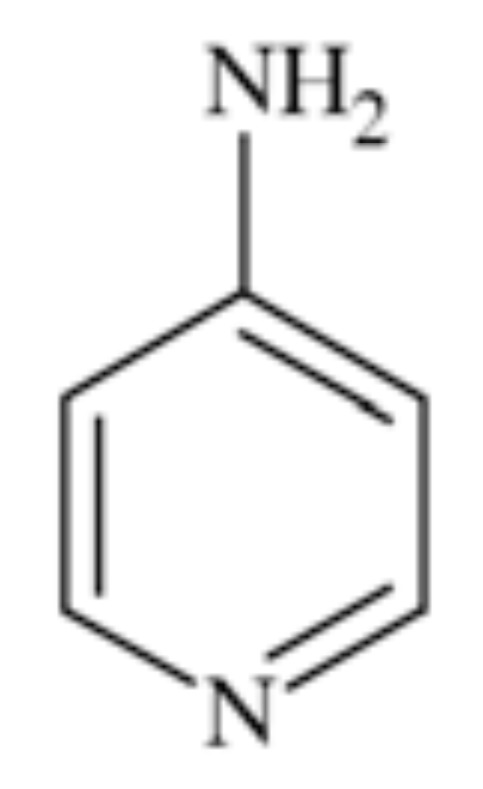
Chemical structure of 4-aminopyridine.

4-Aminopyridine (4-AP, fampridine) Fig. **(22**) inhibits in dose-dependent manner fast voltage-gated potassium channels including K_V_1.1, K_V_1.2, K_V_1.4- K_V_1.7, K_V_3.1- K_V_3.3, K_V_4.1. It also increases Ca^2+^ influx at presynaptic endings. 

In *in vitro* experiments it proved to selectively inhibit so-called A-potassium current, which is characteristic for some members of K_V_. The sensitivity of different channels varied from micromolar to millimolar concentrations of 4-AP and in particular channel the sensitivity depended on its state (open or closed). It was proved that the compound more readily affected open than closed channels and did so from the cytoplasmic site and is not transferred with the pore [107]. K_D_s while inhibiting K_V_2.1 and K_V_3.1 expressed in *Xenopus* oocytes were 17 and 0.08 nM, respectively and the mechanism of action remained similar [108]. It also proved activity in several *in vitro* models of neuronal degeneration, significantly improving function of neurons [107].

4-Aminopyridine proved efficiency in few clinical trials in patients with multiple sclerosis [107]. The compound was also efficient in episodic ataxia type II, probably due to increasing the inhibitory influence of the Purkinje cells [96]. It was approved in January 2010 for patients with MS to improve their walking [97].

## 3 CALCIUM CHANNELS

Calcium channels have been well known drug targets and still they are subject of intensive research. Lately, they have been reveiewed by Belardetti [109]. Voltage-gated calcium channels are present in membranes of excitable cells. They are opened during depolarization and this gives rise to an influx of Ca^2+^. Increase in concentration of this ions stimulates an ongoing depolarization by opening other voltage-gated ion channels. In addition they initiate a release of neurotransmitters [110]. Moreover, calcium ions act as a second messenger and activate enzymatic processes [111]. 

Calcium channels consist of two families: HVA (High Voltage-Activated) and LVA (Low Voltage-Activated). HVA family comprises L-, N-, P-, Q-, and R-type channels. They are heterotrimers of subunits α, β, and α_2_-δ. Subunit α forms a pore with ancillary subunit β. Subunit α_2_-δ forms a functional pore by linking with subunit α. LVA family consists of T-type channels which are monomers and are composed only of subunit α. LVA and HVA differ in function and electrophysiological activity. LVA requires a small amount of depolarization, close to resting membrane potential in order to be opened. In contrast to HVA, they are activated and inactivated very quickly. LVA also display low conductance and better resistance to blockers [112].

A role of Cav1 is to respond to depolarization by inducing intracellular processes. They occur in various sorts of tissues and are responsible for many intracellular activities. In skeletal, smooth muscles and myocytes an influx of Ca^2+^ leads to contraction. In neurons, Ca_v_1 modulate genes’ transcription. Majority of them occur in a cell body and proximal part of dendrites to ease access for Ca^2+ ^to nucleus. In addition they initiate release of neurotransmitters. This subtype of calcium channels have been a cardiovascular targets, however, due to their presence in neurons, they play some role in low-potassium triggered paralysis (Ca_V_1.1), autism, depression, bipolar disorder, or schizophrenia (Ca_V_1.2), and deafness (Ca_V_1.3) [109]. 

Ca_v_2 occur in presynaptic nerve terminals in most CNS synapses. Their main role is to mediate release of neurotransmitters to synapses. Depolarization results in an opening of Ca_v_2 and an influx of Ca^2+^. Ions bind with proteins called synaptogamins, placed on a surface of synaptic vesicles which contain neurotransmitters. Then synaptic vesicles fuse with presynaptic membranes and neurotransmitter is released. The release of neurotransmitter occurs by exocytosis. After that the neurotransmitter binds to a postynaptic receptor, mediating an excitation [113]. Mutations of Ca_v_2 channels can influence its functions. Changes in P/Q-type channels lead to appearance of absence epilepsy. By substitution particular amino acids, an activity of these channels are reduced. In consequence there is a diminished ability to excite neurons [114].

Ca_v_3 mainly occur in neurons and the heart. In CNS, they are responsible for a process of dreaming and their mutation leads to appearance of absence epilepsy [114]. These channels are capable of activation when a membrane potential is close to the resting membrane potential -60mV. A small depolarization triggers an opening of channels and an influx of Ca^2+^. In consequence the depolarization continues by activation Na^+^ and Ca^2+^ channels. At the time of repolarization, these channels deactivate slowly (become closed) which allows to flow of a larger amount of Ca^2+^. In the end of discharge hyperpolarization appears, bringing about closure of inactivated calcium channels [112]. It is essential as depolarization is possible only when calcium channels are closed (deactivated) [115]. 

**Fig. (24):**
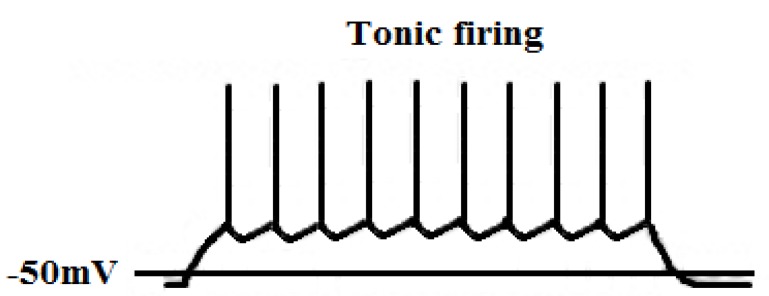
Tonic firing of neurons.

**Fig. (25):**
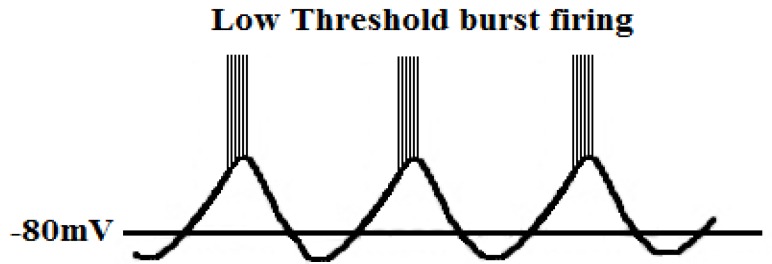
Low-threshold burst firing of neurons.

A feature of Ca_v_3 channels is that they contribute to various models of excitability in neurons. There are 3 models:

1. Tonic firing Fig. **(24**)
  It is a single burst of action potential. If a cell is more depolarized than -60mV for 50 – 100 msec, calcium channels are inactivated, and the cell responds to an excitatory input in tonic mode [115, 116].
2. Low-threshold burst firing Fig. **(25**)
  This type of firing occurs when a membrane is hyperpolarized below -65mV for >50 - 100 msec. The hyperpolarization is essential in order to make calcium channels shift from being inactivated to being closed. During depolarization calcium channels are activated and elicit a low-threshold Ca^2+^ spike (LTS) with a burst of 1–10 action potentials. Because of influx of calcium ions a membrane potential diminish to -40mV. Consequently it leads to an activation of voltage-gated sodium channels [115, 116].
3. Slow oscillations (<1Hz)
  T-type calcium channels produce a large window current in the membrane potential’s range of -60 to -40 mV and are always open and do not inactivate. It is possible because there is an overlap between activation, inactivation, deactivation within this range of membrane potential. It enables a sustained influx of calcium ions and plays an important role in the process of dreaming [117].

### Drug Dependency

L-type calcium channels play a necessary role in sensitization to psychostimulants, seen in an animal model of incentive/motivational aspect of cocaine and amphetamine dependency. Their block in the nucleus accumbens prevents cocaine reinstatement and self-administration in rats [120]. It would be interesting if clinical evidence was provided for use of a Ca_V_1 channel antagonist in treatment of drug dependency.

### Parkinson’s Disease

There is both molecular and clinical evidence that Ca_V_1.3 activity contributes to cell death within *substantia nigra*, leading to deficiency of dopaminergic neurons in this area, which is a known cause for Parkinson’s Disease. From the clinical point of view, cardiovascular patients treated by L-type calcium blockers penetrating blood-brain barrier significantly reduced risk of PD in patients [109].

It has been noticed that mutation in Ca_V_2.1 gene causes familial hemiplegic migraine [121]. Contrary to this fact, Ca_V_2.1 mice are severely ataxic and display absence seizures, followed by death at the age of 4 weeks [122]. Both these facts discourage from targetting Ca_V_2.1 in drug discovery process.

### Pain

N-type (Ca_V_2.2) calcium channels blockers have been a prime target for analgesics as they are expressed in the dorsal horn of the spinal cord. They transmit pain signals from afferent neurons to second order neurons projecting to higher CNS centers. They also are a target for descending activation of noradrenergic pathways by norepinephrine and for inhibition by opioid pathways [123]. The first analgesic drugs to be registered as antagonists of Ca_V_2.2 was ziconotide, a peptide derivative of ω-conotoxin-MVIIA. Its toxicity, which probably resulted from small selectivity to the targets, narrowed the use of these compounds. Intrathecal administration was also a considerable disadvantage so far [109]. However, there are some efforts in drug discovery for analgesics acting by antagonism of Ca_V_2.2 (see below). 

### Absence Epilepsy

Absence epilepsy is associated with generalized epileptic seizures which concerns 10% of population suffering from epilepsy. It is characterized by loss or impaired consciousness, which lasts up to 30 seconds. During an attack of epilepsy the patient displays absent impression on his face and does not respond. It is accompanied by quivering of the eyelids, turning pale and relaxation of facial muscles. Patient experiences amnesia and is not aware of having seizures. 

The cause of absence epilepsy is abnormal hyperexcitability of neurons in a thalamus-cortex circuitry. In EEG records occur oscillation consisted of multiple spike-waves and slow-waves at more than 2.5 Hz, predominantly 3–6 Hz. The thalamus-cortex circuitry comprises:

- Thalamic relay cells of the ventroposterolateral and ventroposteromedial thalamic region
- Pyramidal neurons in the cortex (layers III-VI)
- Reticular thalamic nucleus (RTN) [124]

**Fig. (26):**
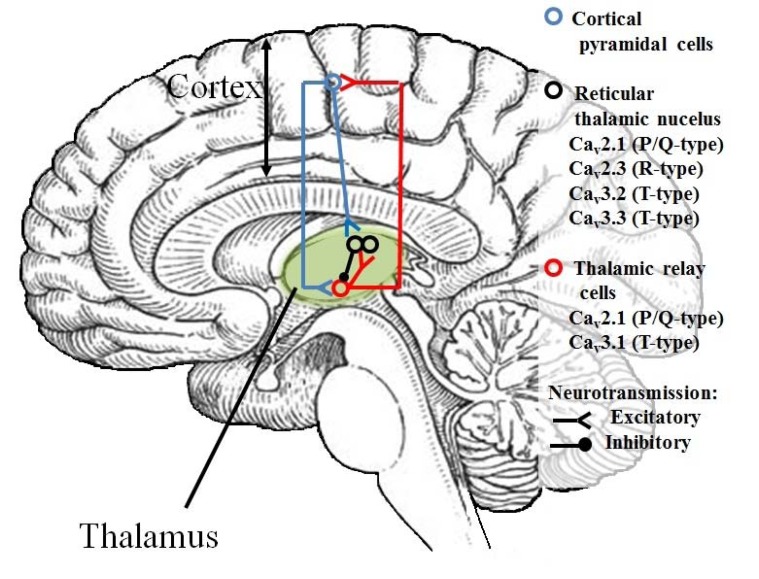
Thalamus-cortex circuitry.

Ca_V_2 and Ca_V_3 types of calcium channels are present in thalamic relay cells and RTN Fig. **(26**). Thus in both structures two models of neurons’ excitability are possible – tonic and burst firing. The tonic firing mode is characteristic of stages of high vigilance. The burst firing is characteristic of dreaming process and some pathological states like absence epilepsy [124].

In a properly working circuitry, thalamic relay cells exert glutamatergic projections on pyramidal neurons in layers III and IV. These pyramidal neurons project to pyramidal neurons in layer V and VI of the cortex. Then they re-innervate thalamic relay cells with glutamate as a neurotransmitter. Both thalamic relay cells and cortex are linked to reticular thalamic nucleus by glutamatergic neurons. RTN comprises only GABAergic interneurons. It is linked with thalamus and projects inhibitory neurotransmitter. GABA leads to hyperpolarization of memebrane of thalamic relay cells and this allows calcium channels to shift from inactivated state to deactivated state. Consequently calcium channels can be opened and the burst firing occurs [124]. In EEG record there are characteristic oscillations. Spikes represent an excitability of neurons during glutamatergic projections. Waves appear when RTN projects inhibitory neurotransmitter [125].

During absence epilepsy seizures, spike-wave discharges are observed in the cortex [126]. These discharges lead to an excitability of reticular thalamic nucleus. RTN projects GABA to thalamic relay cells and causes sustained inhibition of thalamic relay cells. This enables calcium channels to become deactivated enough. As a result, burst firing does not occur. It is claimed that inhibition of thalamus causes loss of consciousness during absence seizures [127]. 

Knock-out of Ca_V_2.1 calcium channels gives rise not only to seizures, but also to ataxia, while gain of function mutations may be related with familial migraine [109]. 

### Nifedipine

**Fig. (27):**
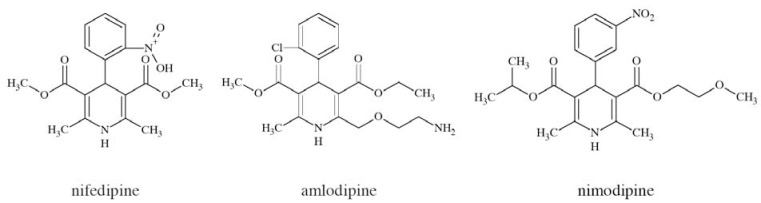
Chemical structures of nifedipine, amlodipine, and nimodipine.

Nifedipine, amlodipine, and nimodipine Fig. **(27**) are dihydropyridine derivatives. They have been developed as cardiovascular drugs, blocking L-type (Ca_V_1) calcium channels. Amlodipine blocks Ca_V_1.2 with IC_50_=100 nM and Ca_V_2.1 with IC_50_=10 µM. Many drugs from the group of dihydropyridines crossing blood-brain barrier, including nifedipine and nimodipine (and excluding amlodipine), proved efficacy in preventing Parkinson’s disease in over 25% of patients [128]. However, in an animal study, nifedipine lowered anticonvulsant activity of topiramate in mice (it doubled its ED_50_). The authors suggest some pharmacodynamic interaction, *e.g.* L-type calcium channel binding of topiramate, rather than pharmacokinetic or any other interaction between nifedipine and topiramate [129]. These facts concerning derivatives of dihydropyridine are premises for drug discovery of more selective derivatives vs. Ca_V_1 channels subtypes.

### Pregabalin

**Fig. (28):**
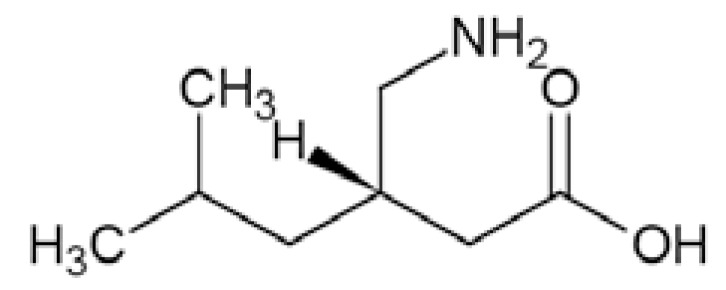
Chemical structure of pregabalin.

Pregabalin (CI-1008, (*S*)-3-aminomethyl-5-methylhexa noic acid) Fig. **(28**), is a derivative of GABA. It binds to the α2δ subunit of the Ca_V_2 calcium channels. Moreover, it increases GABA concentration in neuronal tissues and enhances activity of glutamate decarboxylase. It is a well-known antiepileptic drug, exhibiting both anticonvulsant and analgesic activity in central and peripheral neuropathic pain. [130-132]. 

### Gabapentin

**Fig. (29):**
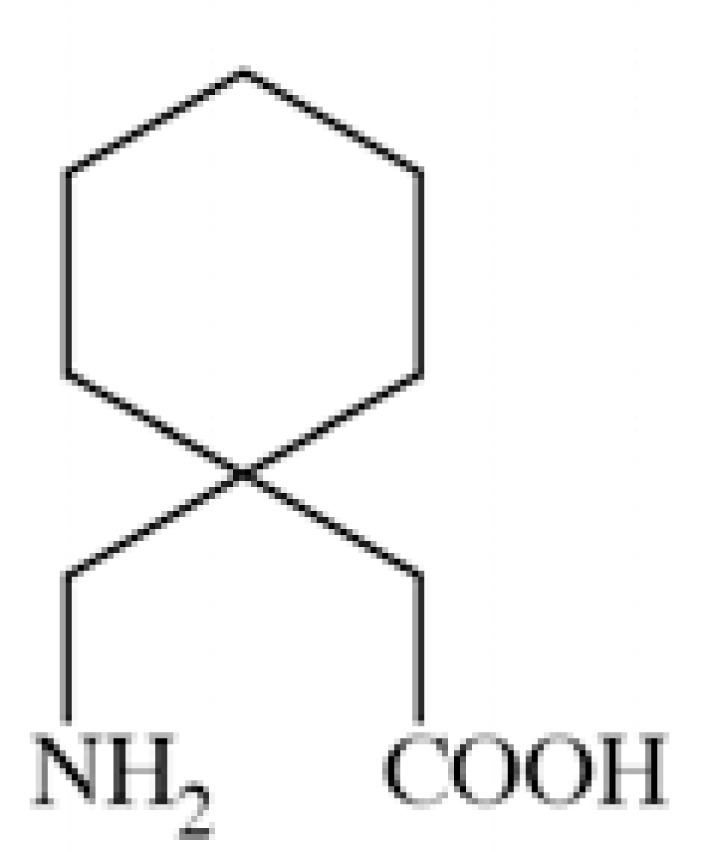
Chemical structure of gabapentin.

Gabapentin (1-(aminomethyl)cyclohexaneacetic acid) ** Fig. (29**) is a GABA derivative. It exhibits binding to α2δ subunit, causing inhibition of Ca_V_2 calcium channels. The additional mechanism is related with the above, *i.e.* it inhibits release of glutamate, relieving neuronal hyperexcitability. It is a well known antiepileptic drug with proved preclinical and clinical efficacy in epilepsy and pain [133, 134].

### Ziconotide

Ziconotide, SNX-111, this antagonist of Ca_V_2.2 is registered for treatment of chronic pain, although it is administered intrathecally [118]. It is a synthetic analog of ω-conotoxin-MVIIA. 

### Pyrazolylpiperidine Derivative

**Fig. (30):**
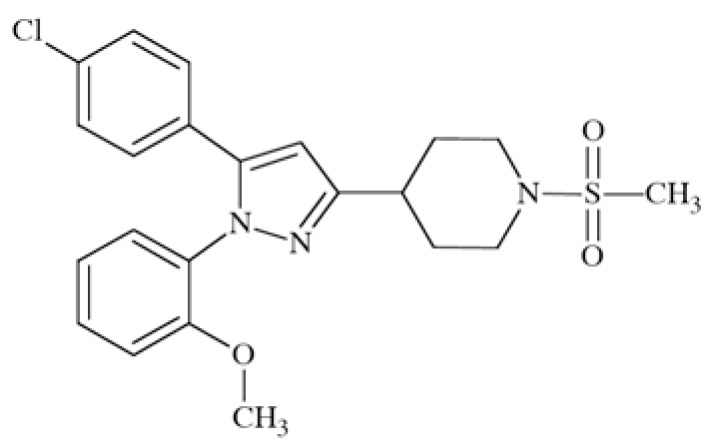
Chemical structure of 1-(2-methoxyphenyl)-3-[4-(1-methylsulfonyl)piperidinyl]-5-(4-chlorophenyl)pyrazol.

This compound (Fig. **(30**) has been synthesized for screening vs. Ca_V_2.2 channel, taking into account the need for an orally administered and metabolically stable anticonvulsant with a similar mechanism of action to ziconotide. It is active in a chronic constriction injury model for neuropathic pain (rats, *i.p.*) at 30 mg/kg b.w. (cold hypersensitivity) and in carrageenan-induced inflammatory pain (thermal hypersensitivity) [119].

### Ethosuximide and Mesuximide

**Fig. (31):**
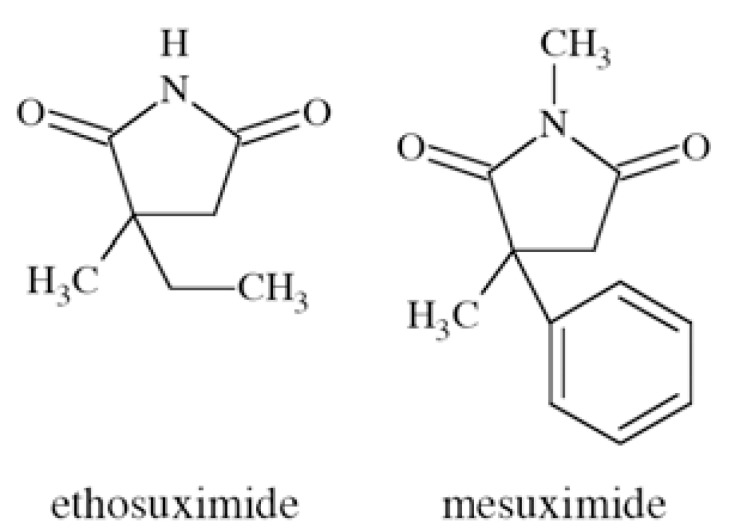
Chemical structures of ethosuximide and mesuximide.

Ethosuximide (3-ethyl-3-methylpyrrolidine-2,5-dione) and mesuximide (1,3-dimethyl-3-phenylpyrrolidine-2,5-dione) ** Fig. (31**) are well established anticonvulsants, active in absence epilepsy. They show affinities towards Ca_V_3 receptors. They are inactive in grand mal seizure models in rodents (*e.g.* MES), and active in petit mal epilepsy model – ethosuximide in scMET test with ED_50_=167 mg/kg b.w. (rats, *p.o.*) [56].

## 4 P2X RECEPTORS

Extracellular adenosine 5’-triphosphate (ATP) is an important excitatory transmitter both in the peripheral and central nervous system [135]. P2X receptors are a distinct family of ligand-gated membrane ion channels activated by extracellular ATP. Human P2X receptors are a family of seven isoforms designated P2X1 to P2X7. The isoforms share 26–54% sequence identity and each subunit is between 379 and 595 amino acids in length [136-138]. P2X1, P2X2, P2X3, P2X4, P2X5, and P2X7 isoforms form functional homomeric receptors, however heteromeric assemblies such as P2X2/3 and P2X1/5 channels are also possible [139, 140]. P2X7 receptors are unique among the P2X receptor family because they fail to form heteromeric forms and are activated by high ATP concentrations (>100 µM) [141]. 

**Fig. (32):**
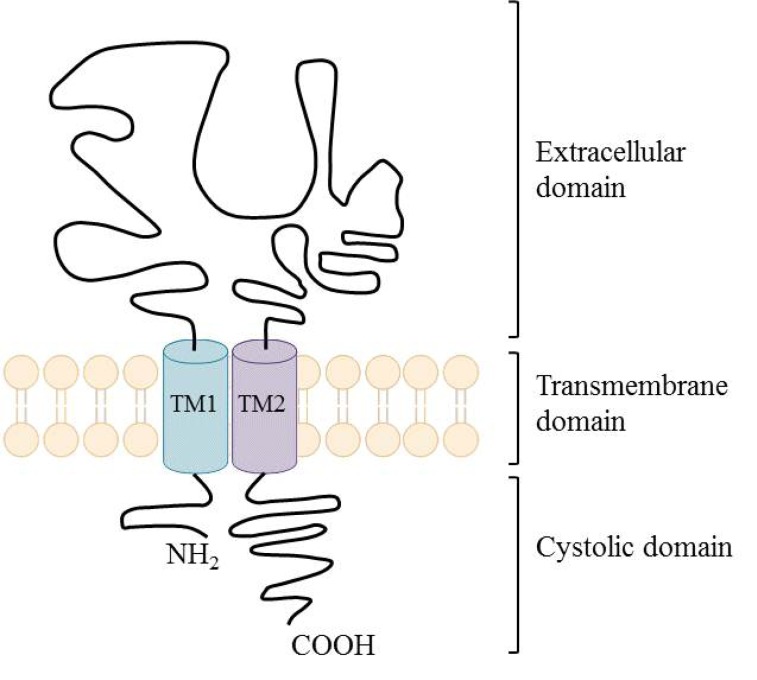
Schematic structure of P2X receptor.

P2X receptors (Fig. **(32**) are organized as trimers [142] with the subunits arranged in a “head-to-tail” order [143]. All have two transmembrane domains (TM1 and TM2), an extracellular ligand binding loop that contains ten conserved cysteine residues that form disulphide bonds and intracellular amino and carboxy termini [139, 144]. Both TM1 and TM2 are thought to line an integral ion pore [145]. Thus, the resulting channel is composed of six transmembrane domains, two from each of three subunits, the structure that is unique in the ion channel literature. 

The primary agonist of all P2X receptors is ATP and at least 3 molecules of ATP are bound to the extracellular portions of open P2X channels [143]. P2X receptors undergo conformational changes leading to the opening of a cationic pore within milliseconds of binding ATP [146]. P2X receptors display significant calcium permeability. Moreover, they show sodium and potassium permeability [136] and some of them are also permeable to chloride [147]. Aside from ATP, most P2X receptors are also activated by diadenosine polyphosphates or related dinucleotides and some nucleoside triphosphates [140, 148]. 

P2X receptors are widely distributed in cell types of nearly every origin, including neuronal, epithelial, immune and muscular. They are involved in actions such as synaptic transmission in the peripheral and central nervous systems, contraction of smooth muscle, platelet aggregation, macrophage activation, cell death and immunomodulation [149, 150].

P2X receptors are widely expressed on central and peripheral neurons - with P2X2, P2X3, P2X4 and P2X7 being the most abundant in the nervous system [151]. P2X receptors have been explored as therapeutic targets, *e.g.*, for chronic inflammatory diseases and pain. In particular, P2X3 and P2X7 receptor antagonists have been developed and exhibited antinociceptive or antiinflammatory activity in animal models of these diseases [152]. 

Apart from neuropathic pain conditions it has also been reported that the activation of P2X receptors may be involved in the process of excitotoxic neuronal injury caused by stroke [153]. It was proved that following ischemia especially P2X7 receptors are upregulated on neurons and glial cells in rat cerebral cortex [154]. The evidence from prior studies, however, showed that the deletion of P2X7 receptors (knock-out mice) and/or treatment with KN62, the antagonist of P2X7 receptors had minimal effect on ischemic cell death [155]. Another study showed that P2X receptors are involved in the mechanisms sustaining cell death evoked by metabolism impairment [156]. Additionally, both P2X4 and P2X7 receptors expressed on microglia might be involved in cortical damage produced by oxygen and/or glucose deprivation [157]. Several earlier studies have confirmed the idea as P2 receptors antagonists were able to abolish the cell death fate of primary neurons exposed to excessive glutamate [158], to hypoglycemia or chemical hypoxia [159, 160] and to serum/ potassium deprivation [161]. 

P2X7-mediated signaling is also implicated in neurodegenerative processes observed in CNS diseases, such as Parkinson’s and Alzheimer’s diseases and multiple sclerosis [162-166]. In case of Parkinson’s disease the release of ATP from disrupted cells might cause cell death in neighbouring cells expressing P2X7 receptors, consequently necrotic volume is increased [167]. Moreover, P2X7 receptors are specifically upregulated in the brain of patients with Alzheimer’s disease (AD) and in animal models of the disease [168]. 

In two different transgenic models of Huntington’s disease changes in P2X receptor-mediated neurotransmission in cortico-striatal projections have been identified. In addition, it was shown that P2X receptor antagonists might have potential as neuroleptic agents as microinjection of ATP analogues into the prepiriform cortex was able to induce generalized motor seizures [169]. Purinergic mechanisms and specific P2X receptor subtypes have also been involved in the pathogenesis of clinical conditions such as amyotrophic lateral sclerosis [170] and epilepsy [171, 172].

### Suramin

**Fig. (33):**
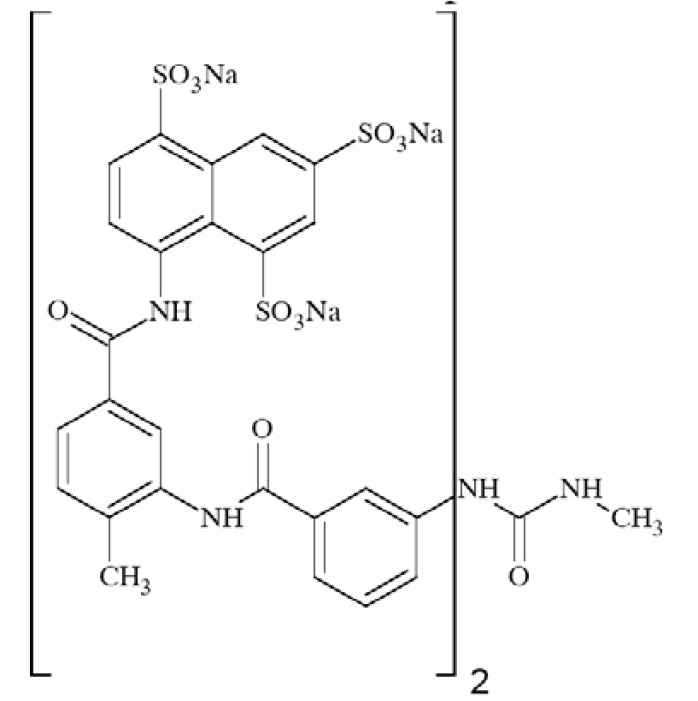
Chemical structure of suramin.

Suramin (Fig. **(33**) the best known antagonist of P2X receptors [173] was assessed for its neuroprotective potential in a model of experimental stroke in rats. In the study focal brain ischemia was induced by unilateral occlusion and transection of the middle cerebral artery (MCAT) and bilateral occlusion of the common carotid arteries (CCA). It was shown that suramin at a dose of 100 mg/kg significantly decreased both infarct and edema volume. Thus, the compound is an effective pretreatment neuroprotective agent [153].

### CE-224535

**Fig. (34):**
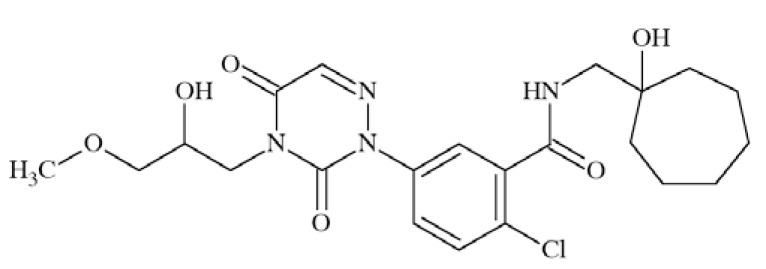
Chemical structure of CE-224535.

Pfizer’s compound CE-224535 (Fig. **(34**), a P2X7 receptor antagonist was recently evaluated in the clinic for the treatment of rheumatoid arthritis [174]. The compound is presently studied for the treatment of other conditions, such as pain and Alzheimer´ s disease [175]. 

## 5 NMDA RECEPTORS

**Fig. (35):**
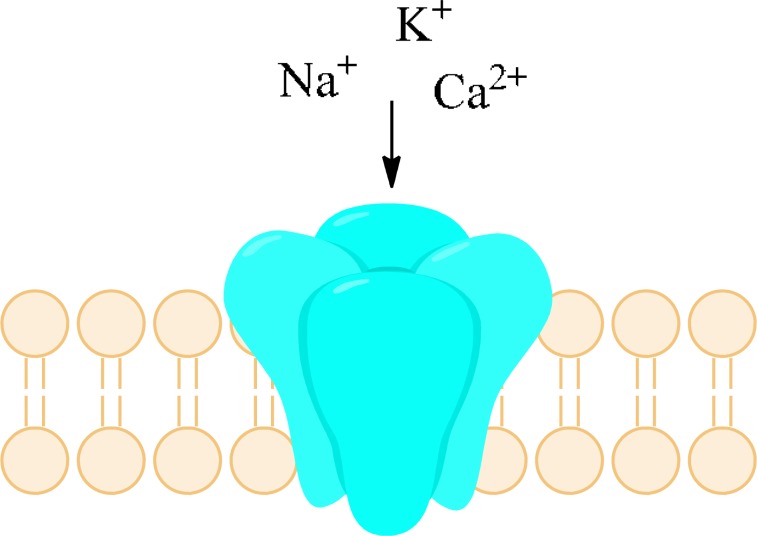
Schematic structure of NMDA receptor.

N-methyl-D-aspartate (NMDA) receptors (Fig. **(35**) are a subtype of ionotropic glutamate receptors widely distributed in CNS. NMDA receptors play a crucial role in synaptic transmission, neural plasticity and neurodegeneration [176, 177], thus may serve as potential CNS therapeutic targets [178]. 

NMDA receptors are hetero-oligomeric ligand-gated cation channels comprising four NMDA subtypes: NR1, NR2, and NR3 [179]. The subtypes include the ubiquitously expressed NR1 subunit; a family of four distinct NR2 subunits (A, B, C, and D); and more rarely two NR3 subunits (NR3A and NR3B) [180, 181]. Most NMDA receptors are believed to assemble as tetramers, associating two NR1 and two NR2 subunits in a ‘dimer of dimers’ quaternary architecture [181-183]. The subunits differ in their permeation and block by divalent ions and sensitivity to various endogenous and exogenous ligands. It was discovered that NR1 and NR2A subunits are the most common and NR2D are the rarest. More heterogeneous receptors exist in CNS, comprising multiple types of NR1 and/or NR2 subunit in one receptor complex. Thus, NMDA receptors including NR1/NR2B/ NR2A and NR1/NR2B/NR2D can be distinguished in many areas of the CNS [184, 185]. 

NMDA receptors’ intrinsic ion channel is permeable to monovalent cations such as sodium and potassium, and bivalent ones including calcium. In order to open the channel pore by NMDA receptors the simultaneous binding of glutamate (neurotransmitter role) and the co-agonist – glycine (or D-serine, modulators) is needed. The glutamate binding site is situated on the NR2 subunit and the glycine binding site is situated on the NR1 subunit. NMDA receptor activation takes place after presynaptic glutamate release and with appropriate glycine concentration in the synaptic cleft [177, 178, 182]. 

In general, NMDA receptors are found in many but not all cerebral cortex neurons and some cortical astrocytes [178, 186] and play major roles in both physiological and pathological states of the CNS. Inappropriate activation of NMDA receptors has been stated in the etiology of various disorders. 

### Stroke

The first clinical indication considered for NMDA receptor antagonists was stroke. In such ischemic or hypoxic conditions the increased amount of glutamate is released into the synaptic clefts, thus the overstimulation of the NMDA receptor is observed [187]. It was proved that the blockade of NMDA receptors resulted in neuroprotective effect in animal models of stroke [188]. Also in traumatic brain injury (TBI) glutamate is released in large quantities into the extracellular fluid by neurons and glial cells. This was confirmed in both animal models and human studies [189, 190]. Thus, the application of NMDA antagonists might be of therapeutic benefit.

NMDA receptors *via* calcium fluxes are able to down-regulate the stream signaling pathways leading to short-term and long-term neuronal changes [191]. Thus, alternation in these receptors causes deficits in learning and memory [192].

### Schizophrenia

Additionally, both genetic and pharmacological studies showed that glutamatergic dysfunction, particularly with involvement of NMDA receptors plays a vital role in the pathophysiology of schizophrenia [193, 194]. It was proved that mRNA encoding the NR2B subunit was increased in hippocampal and cortical regions in schizophrenia [195, 196]. Arias *et al.* [197] suggested that hyperphosphorylation of NR2B-containing receptors *in vivo *may cause increase of sensitivity to the endogenous transmitter, and induction of neuronal hyperexcitability and epilepsy.

### Parkinson’s Disease

In the pathophysiology of Parkinson’s disease (PD) over-reactivity of the glutamatergic pathway may be stated. Thus, the manipulation of the glutamatergic system may show anti-parkinsonian effects [198]. Various studies indicated that the NR2B subunit is a important molecular component in the etiology of PD and therefore should be considered as a potential therapeutic target for the treatment of the disease [199-203].

### Huntington’s Disease

Another severe disease related with NMDA receptors is Huntington’s disease (HD). It was confirmed that NR2B subtype NMDA receptors activation triggers the selective degeneration of the medium spinal striatal neurons in HD [204]. Mutant huntingtin may increase the number of functional NR1/NR2B-type receptors at the cell surface [205]. Moreover, the expression of polyglutamine-expanded huntingtin results in NMDA receptors sensitization and promotes neuronal apoptosis triggered by glutamate [206]. 

### Gavestinel

**Fig. (36):**
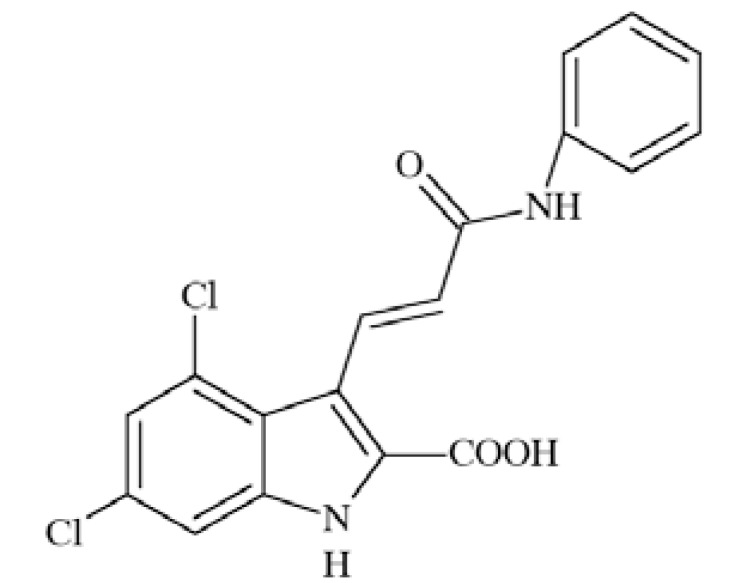
Chemical structure of gavestinel.

Gavestinel (Fig. **(36**) a selective antagonist at the glycine site of the NMDA receptor and one of the indole derivatives was evaluated in various animal models of stroke. In the middle cerebral artery occlusion (MCAO) model of focal ischemia in rats administration of the compound (3 mg/kg *i.v.*) up to 6 h from occlusion resulted in a significant reduction of the infarct volume measured histologically 24 h later [207]. Moreover, gavestinel was characterized in terms of *in vivo *potency by inhibition of convulsions induced by *N*-methyl-D-aspartate in mice. The compound inhibited convulsions induced by NMDA when administered by both *i.v.* and *p.o*. routes (ED_50_= 0.06 and 6 mg/kg, respectively) [187]. Also magnetic resonance imaging confirmed the potential neuroprotective activity of gavestinel when administered either before or up to 6 h after ischemia [208]. Gavestinel was well tolerated in early human studies and showed no apparent significant CNS or hemodynamic effects [209, 210]. In a randomised, double-blind, placebo-controlled trial to test whether gavestinel could improve functional outcome after acute stroke in human beings the treatment with the compound within 6 h of acute ischemic stroke did not improve the outcome [211]. Additionally, in clinical trials of this compound in patients with intracerebral hemorrhage gavestinel was not of substantial benefit or harm to patients [212]. 

### Selfotel

**Fig. (37):**
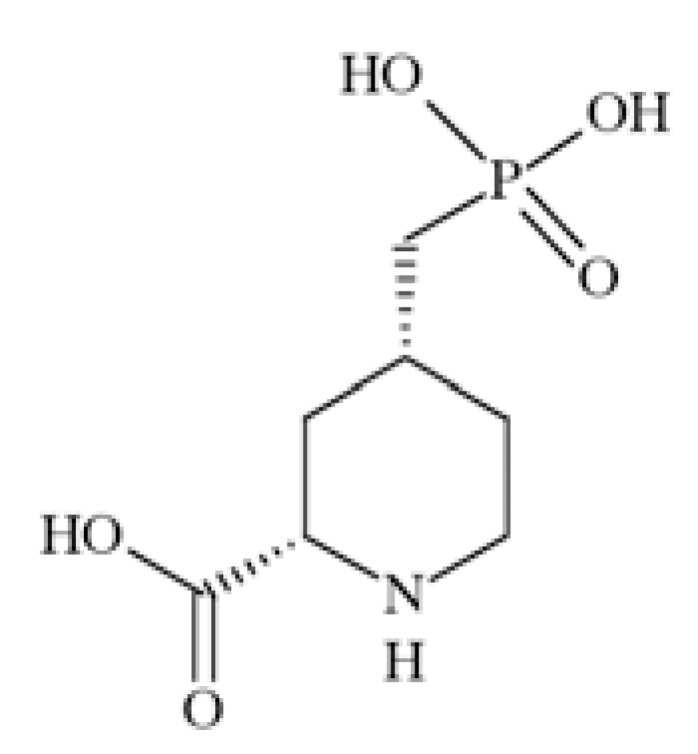
Chemical structure of selfotel.

The competitive glutamate antagonist selfotel (CGS 19755) (Fig. **(37**) in preclinical animal tests produced anticonvulsant, anxiolytic and anti-ischemic effects [213]. The compound reduced neuronal damage in animal models of stroke [214-216]. In the rat model of global cerebral ischemia selfotel treatment (dose ranged from 10 to 30 mg/kg intraperitoneally) resulted in neuroprotection that was observed when administration was not delayed more than 30 minutes after the onset of ischemia [215]. The neuroprotective effect of selfotel was also confirmed in rats with occluded left middle cerebral and common carotid arteries. Intravenous infusion of selfotel (10 mg/kg) reduced infarct volume [214]. The compound was also effective in a rabbit model of reversible CNS ischemia [216]. Grotta *et al.* [215] evaluated the safety and tolerability of selfotel in patients with acute ischemic stroke. The study showed that although adverse effects are related with the treatment in a dose-dependent fashion, a single intravenous dose of 1.5 mg/kg is safe and tolerable with adverse experiences that are controllable. In Phase III efficacy trials conducted in patients with severe traumatic brain injury the compound (four doses of 5 mg/kg each, 24 h apart) failed to demonstrate a satisfactory risk/benefit ratio. One explanation is that the compound which is competitive with glutamate at the receptor may not influence events at the receptor site [217].

### Aptiganel

**Fig. (38):**
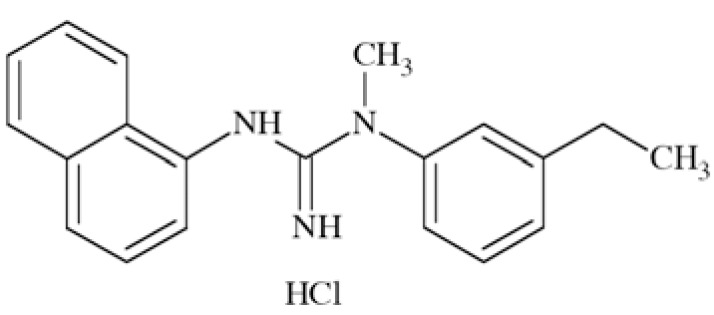
Chemical structure of aptiganel.

Aptiganel (Fig. **38**) acts as a noncompetitive NMDA antagonist [218]. In a rat model of human ischemic stroke, aptiganel hydrochloride administered intravenously was able to reduce (by 40-70%) the volume of brain damage and the accompanying neurological dysfunction. Moreover, in a rat model of temporary focal ischemia the compound was able to protect both cerebral gray matter and white matter from ischemic injury [219]. The study on controlled cortical impact injury of the left temporoparietal cortex in rats demonstrated that the compound decreased contusion volume and hemispheric swelling as well as water content [220]. Neuroprotective activity of aptiganel HCl was also confirmed in controlled cortical impact injury (CCII) in rats. Compound’s administration was associated with decrease contusion volume, less hemispheric swelling, a lower intracranial pressure and increased cerebral perfusion pressure [221].

In a dose-ranging, placebo-controlled, phase I study Muir *et al.*, [1994] confirmed that aptiganel’s pharmacokinetic effects are favourable for a potential neuroprotective therapy. 

In human volunteers, however, dose-limiting effects of aptiganel were blood pressure increases and an excess of CNS. In another study both the safety and tolerability of aptiganel were assessed in patients with acute ischemic stroke. It was demonstrated that a 4.5-mg intravenous bolus of aptiganel HCl followed by infusion of 0.75 mg/h for 12 h is a tolerable dose that can produce plasma drug concentrations shown to be neuroprotective in animal models [222]. Albers *et al.* [223] determined whether aptiganel improves the clinical outcome in acute ischemic stroke patients. The compound administered in the tested doses within 6 h of symptom onset is not effective in the treatment of patients with acute ischemic stroke and may be harmful. 

As non-selective NMDA receptor antagonists have impeding adverse side effects, attention has been directed towards compounds capable of modulating selectively certain subtypes of these receptors. In this field the NR2B-selective type of non-competitive antagonists has a strong potential, showing both analgesic and neuroprotective activity together with slight side effects [178, 224]. Many NR1/NR2B antagonists, including ifenprodil, eliprodil and the selective and potent congeners, traxoprodil and Ro 25-6981, offer promise in preclinical models of ischemia [225-229].

### Ifenprodil

**Fig. (39):**
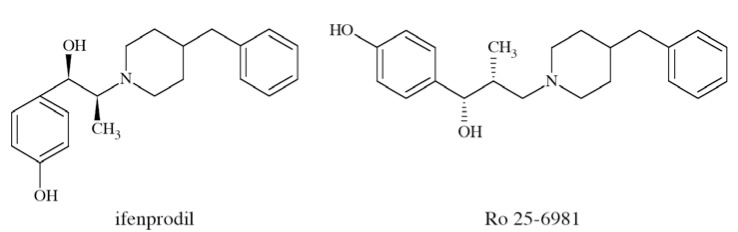
Chemical structures of ifenprodil and Ro 25-6981.

Ifenprodil (Fig. **(39**) NR2B selective NMDAR antagonist, when administered as a perfusion (0.3-3 mg/kg *i.v.*) over 3 hours after occlusion of the feline middle cerebral artery decreased the infarcted tissue volume (measured 4 days after occlusion) in a dose-related manner. At the highest dose a 42% reduction of infarcted volume was observed, substantially in cortical tissue [225]. The interaction of compound Ro 25-6981 (Fig. **(39**) with NMDA receptors proved that Ro 25–6981was more potent than ifenprodil [230]. 

### Eliprodil

**Fig. (40):**
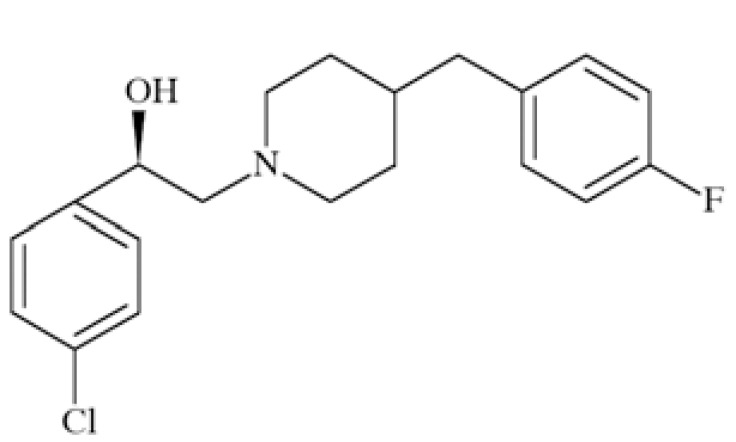
Chemical structure of eliprodil.

The neuroprotective activity of eliprodil (Fig. **(40**), NMDA receptor antagonist acting at the polyamine modulatory site, in brain trauma was examined in a rat model of lateral fluid-percussion brain injury. Eliprodil (10 mg/kg *i.p.*), reduced by 60% the volume of cortical damage. Moreover, the neuroprotective activity of the compound in experimental brain trauma using neuropathology was documented [226]. Eliprodil reduces lesion volume following traumatic brain injury [231].

### Taxoprodil

**Fig. (41):**
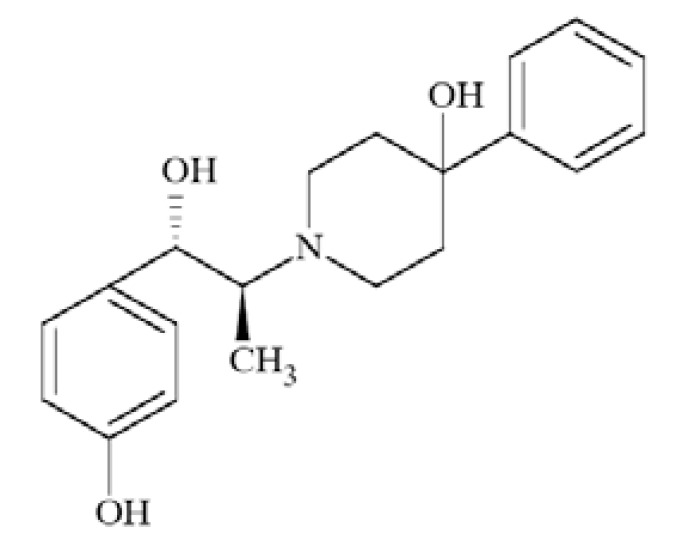
Chemical structure of traxoprodil.

The neuroprotective effect of traxoprodil (Fig. **(41**) - a competitive NMDA antagonist at NR2B binding subunit, was evaluated in a rat subdural hematoma (SDH) model. The drug was infused 30 min after induction of SDH. The reductions of ischemic brain damage achieved by traxoprodil was 29-37% [228]. Moreover, this drug exhibits a 69% reduction in infarct size in comparison to controls [232]. In humans it was well tolerated, penetrated the brain, and showed improvement in brain injury [232]. In a double-blind, placebo-controlled study of the safety, tolerability and pharmacokinetics of traxoprodil in patients with a mild or moderate traumatic brain injury it had no psychotropic effects and was well-tolerated in patients [232]. Moreover, the compound had direct antiparkinsonian actions in rodents (decreased haloperidol-induced catalepsy with ED_50_=0.5 mg/kg) and monkeys (1-methyl-4-phenyl-1,2,3,6-tetrahydropyridine (MP TP)- treated monkeys, traxoprodil (1 mg/kg)) [233]. Moreover, no side effects were apparent at any dose of traxoprodil. The drug also reduced the maximum severity of levodopa-induced dyskinesia approximately by 30% but it did not improve Parkinsonism. Side effects were dose-related dissociation and amnesia [234], although in another study the drug was well tolerated, although no efficacy was seen in severe TBI [235].

### Ro 63-1908

**Fig. (42):**
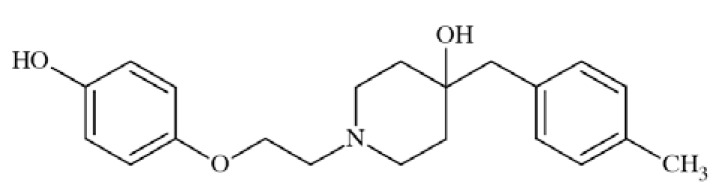
Chemical structure of Ro 63-1908.

Ro 63-1908 (Fig. **(42**) is a potent and highly selective antagonist of the NR2B subtype of NMDA receptors, active versus sound-induced seizures (ED_50_=4.5 mg/kg *i.p.*, DBA/2 mice). It also inhibited NMDA-induced seizures with an ED_50_ =2.31 mg/kg *i.v*. In addition, Ro 63-1908 gave a dose-related neuroprotective effect against cortical damage in a model of permanent focal ischemia. Maximum protection of 39% was seen at a plasma concentration of 450 ng/ml. There were no adverse cardiovascular or CNS side-effects observed at these active doses [236].

### Amantadine

**Fig. (43):**
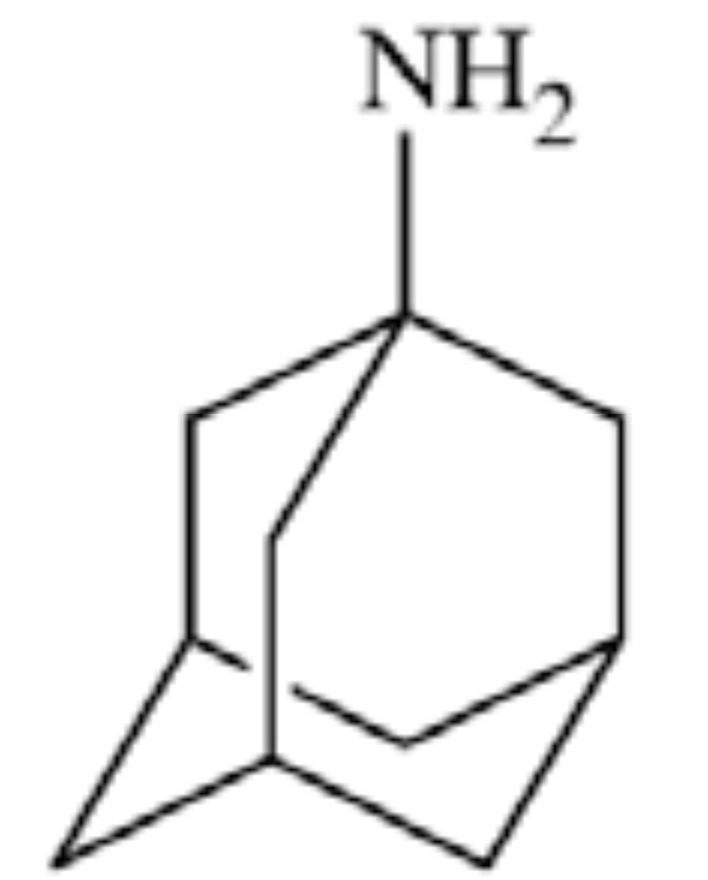
Chemical structure of amantadine.

Amantadine (Fig. **(43**), a low affinity NMDA antagonist represents an approach for treating Huntington’s disease [237]. Amantadine is also used as an adjuvant therapy in Parkinson’s disease. The compound exhibited efficacious effects in Parkinson's disease patients suffering from dyskinesias [238]. 

### Memantine

**Fig. (44):**
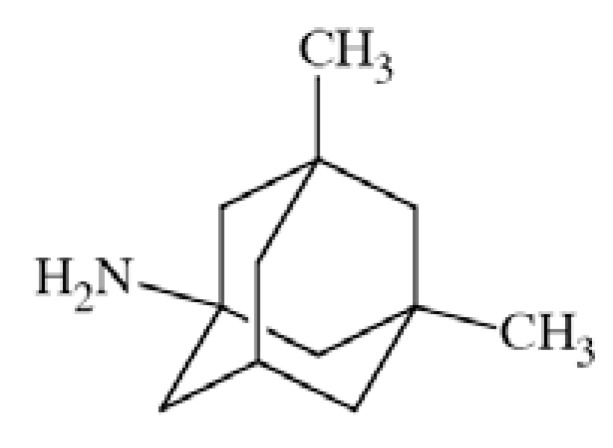
Chemical structure of memantine.

Memantine Fig. **(44**) is another low affinity NMDA receptor antagonist. The compound was tested for non-motor features of Parkinson’s disease and despite good tolerability the specific measures of sleepiness, fatigue, depression, and attention did not significantly improve [239]. It decreases probable REM sleep behavior disorder in patients with Parkinson's disease dementia [240]. It leads to improvements in cognitive functions, stabilization of motor impairments, and decreases in the severity of mental disorders, especially in patients with hyperhomocysteinemia [241, 242].

Memantine has also been studied as a treatment for Alzheimer’s disease (AD). It was associated with slowing of right hippocampal atrophy, and with improvement on one test of executive functioning as well as a test of confrontation naming ability [243]. 

### Dexanabinol

**Fig. (45):**
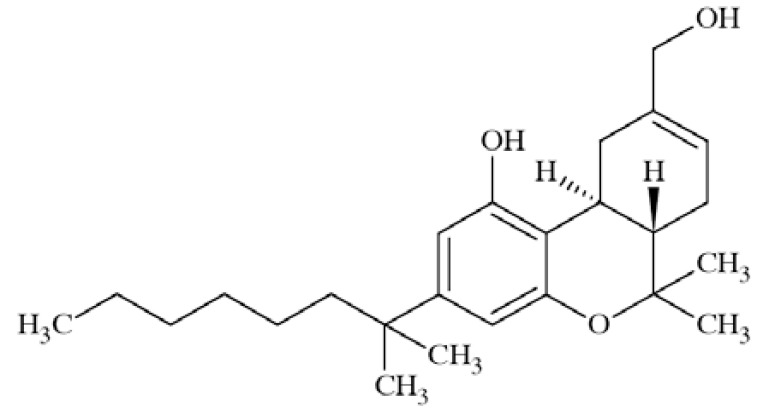
Chemical structure of dexanabinol.

Dexanabinol Fig. **(45**) belongs to non-competitive NMDA antagonists. Treated patients achieved significantly better intracranial pressure/cerebral perfusion pressure control without jeopardizing blood pressure. A trend toward faster and better neurologic outcome was also observed [244]. Further studies (phase III randomized, placebo-controlled clinical trial) showed that dexanabinol is safe, but not efficacious in treatment of TBI [245].

### Remacemide

**Fig. (46):**
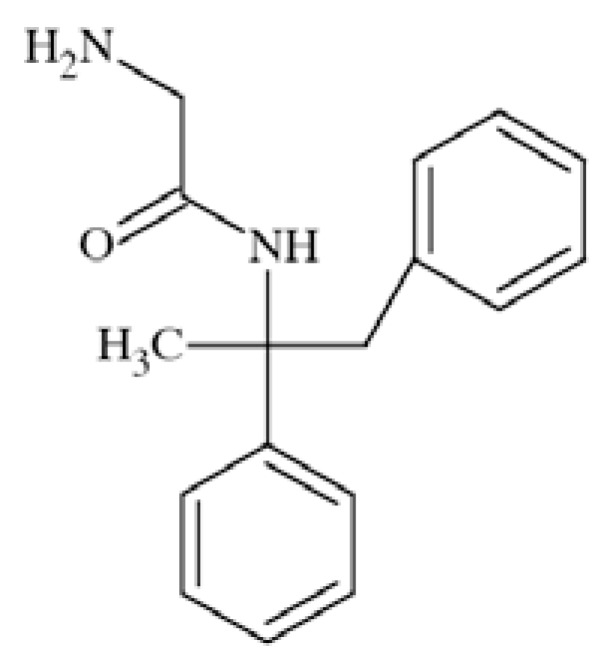
Chemical structure of remacemide.

Remacemide (Fig. **(46**) belongs to low affinity non-competitive NMDA antagonists. The drug exerts anticonvulsant activity both in various animal seizure models and in clinical studies. In addition, it seems to provide neuroprotection [246]. The antiepileptic effects include activity in genetic absence epilepsy rats from Strasbourg (GAERS), and in an audiogenic rat model. The study showed the effects of remacemide in tonic/clonic seizure, which was the first target of the drug, and confirm the effect of the anticonvulsant on absence seizures [247]. Moreover, no serious adverse events were observed [248]. Pretreatment with remacemide has a moderate neuroprotective effect against status epilepticus-induced neuronal damage [249]. 

Remacemide hydrochloride might also improve symptoms of Parkinson’s disease by modulating glutamatergic overactivity in the basal ganglia or slow worsening by decreasing excitotoxicity. In rodent and primate models of Parkinson’s disease systemically administered compound has antiparkinsonian activity and low potential for side effects. It can be a safe and tolerable adjunct to dopaminergic therapy for patients with PD and motor fluctuations [250]. 

### Neramexane

**Fig. (47):**
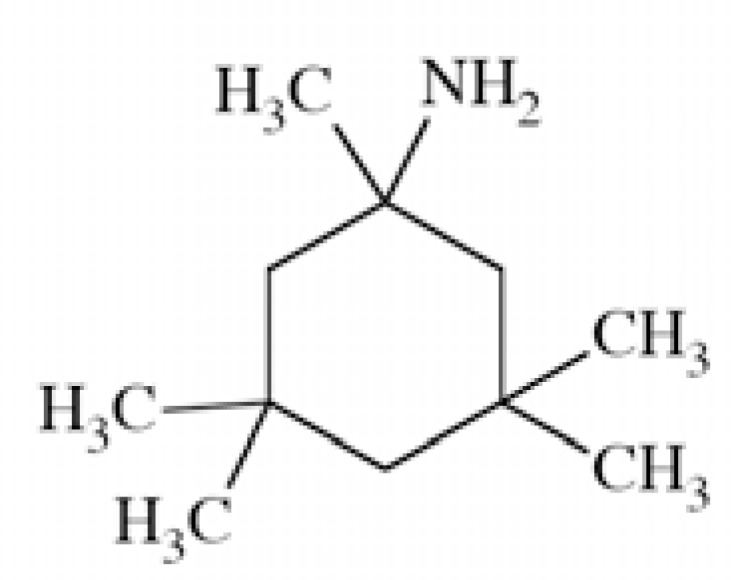
Chemical structure of neramexane.

Neramexane (Fig. **(47**) similarly to memantine, is an open-channel NMDA receptor blocker. The compound displays a similar pharmacokinetic and comparable clinical tolerability. It enhanced a long-term spatial (hippocampus-dependent) memory in adult rats, thus it was concluded that may be helpful in treatment of dementia [251].

### Dimebon

**Fig. (48):**
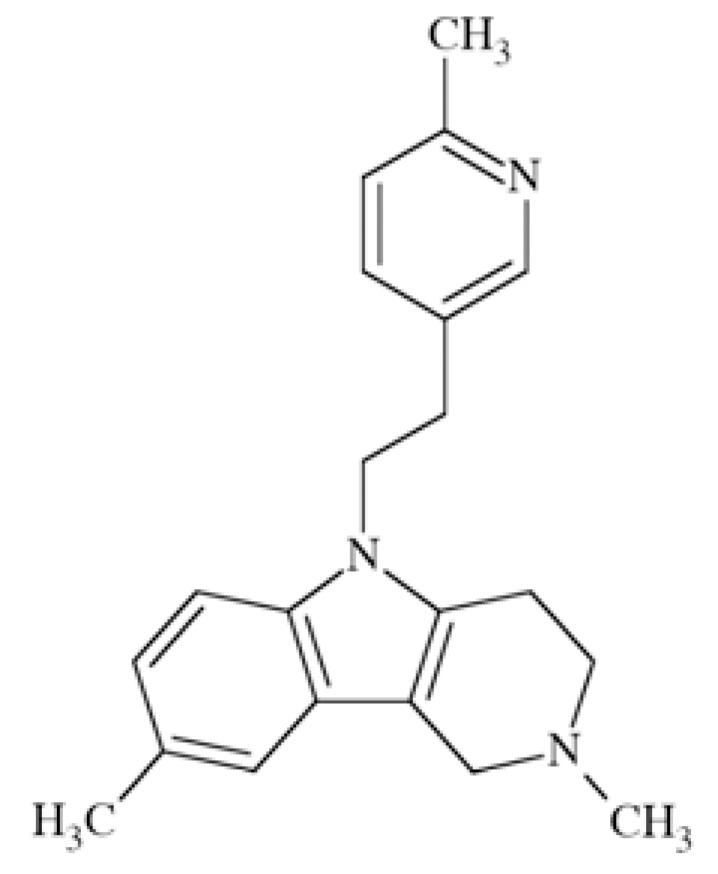
Chemical structure of dimebon.

Dimebon (Fig. **(48**) – an NMDA blocker at a site distinct from memantine, was initially classified as an antihistaminic drug. Systemic administration improves learning in animals with experimental model of Alzheimer's disease [252]. A randomised, double-blind, placebo-controlled study showed that dimebon was safe, well tolerated, and significantly improved the clinical course of patients with mild-to-moderate Alzheimer's disease [253].

### D-Cycloserine

**Fig. (49):**
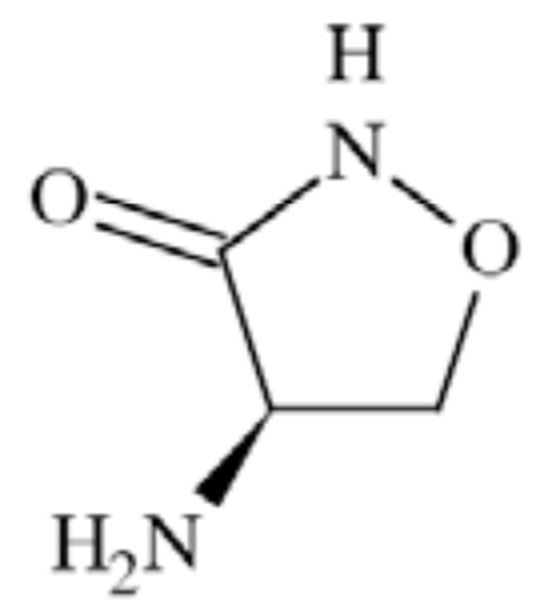
Chemical structure of cycloserine.

The antibiotic D-cycloserine (Fig. **(49**) is a partial glycine site agonist that improves glutaminergic activity in schizophrenia. This preliminary evidence suggests that D-cycloserine may improve negative symptoms and cognitive deficits over a narrow dose range when added to conventional antipsychotic agents [254]. Studies confirmed potential role of D-cycloserine in the treatment of negative symptoms in schizophrenia. It was stated however that the degree of symptom reduction may be modest [255]. Preliminary studies with once-weekly administration showed that D-cycloserine may play potential new role in schizophrenia by demonstrating benefit for negative symptoms, memory consolidation, and facilitation of cognitive behavioral therapy for delusions [256].

### Felbamate

**Fig. (50):**
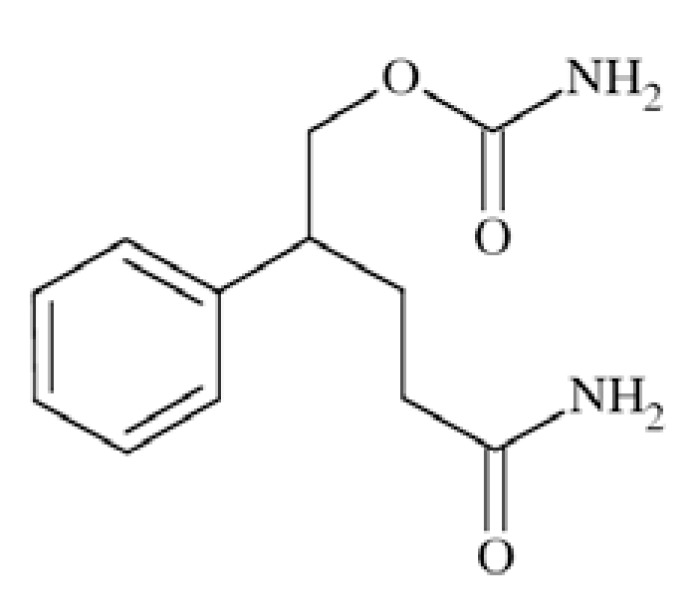
Chemical structure of felbamate.

Felbamate (Fig. **(50**) a non-competitive NMDA antagonist, showed a broad anticonvulsant profile similar to valproic acid when tested in experimental models. The compound blocks NMDA receptors but fails to exhibit the neurobehavioral toxicity characteristic of other NMDA receptor antagonists. It exhibits modest selectivity for NMDA receptors composed of NR1a/NR2B subunits. This selectivity could, in part, account for the more favorable clinical profile of felbamate in comparison with NMDA receptor antagonists that do not show subunit selectivity [257]. The studies indicated that felbamate is an effective anticonvulsant drug in DBA/2 mice [258]. The *in vitro* antiepileptic activity of the novel anticonvulsant drug felbamate was tested in rat hippocampal slices on the CA1 epileptiform bursting induced by different chemical epileptogenic agents.

## 6 ACID-SENSING ION CHANNELS

Acid-sensing ionic channels (ASICs) are almost ubiquitous in the mammalian nervous system, both at the periphery and in the brain [259]. They belong to H^+^-gated subgroup of the degenerin/epithelial Na^+^ channel (DEG/ENaC) family of cation channels [260]. In 1981, Krishtal and Pidoplichko described for the first time a receptor for H^+^ that carried inward Na^+^ currents in mammalian sensory neurons [261, 262]. The receptor was first cloned from the rat brain in 1997, by the group of Lazdunski [263]. Then, receptor was crystallized in 2007 [264]. 

ASICs appear to be the simplest of ligand-gated channels [263]. They are preferentially permeable to Na^+^, but to a lesser extent can also conduct other cations (*e.g.* Ca^2+^, K^+^, Li^+^ and H^+^). Individual subunits consist of two transmembrane domains, relatively short intracellular carboxy and amino termini and a large, cysteine-rich, extracellular domain [265, 260]. Investigation of the ASIC2a subunit showed that the pre-TM2 region is essential for pH sensitivity and gating [266]. ASICs are expressed in neurons and assemble into homomultimeric and heteromultimeric complexes. It is not known precisely how many subunits are required to form a channel. Some researchers, by the analogy with other DEG/ENaC channels suggested four to nine ASIC subunits [267] More recent studies concerning crystallization of a chicken ASIC1 in low-pH proved homotrimeric structure, where each subunit of the chalice-shaped homotrimer is composed of short amino and carboxy termini, two transmembrane helices, a bound chloride ion and a disulphide-rich, multidomain extracellular region enriched in acidic residues and carboxyl-carboxylate pairs within 3 Å, suggesting that at least one carboxyl group bears a proton. Between the acidic residues and the transmembrane pore there is a disulphide-rich ‘thumb’ domain poised to couple the binding of protons to the opening of the ion channel, thus demonstrating that proton activation involves long-range conformational changes [264, 268].

Individually expressed subunits form homomultimeric channels, whereas coexpression of 2 or more subunits allows formation of heteromultimeric channels. Surprisingly enough, ASIC2b does not form H^+^-gated channels when expressed alone but alters current properties when coexpressed with other ASICs. Coexpressing various subunits produces heteromultimers, with H^+^-gated currents revealing characteristics that in some cases reflect ‘average’ properties of contributing subunits and in other cases properties that lie outside the range of the individual subunits (*e.g.* rate of desensitization). Subunit composition also alters ion selectivity; for example ASIC1a homomultimeric complexes are permeable to Ca^2+^, whereas ASIC2-containing heteromultimeric complexes are not [260].

In heterologous cells, ASIC homomultimeric channels vary in terms of activation and desensitization kinetics as well as pH sensitivity. For example, in one study, the pH values required for half-maximal activation of murine ASIC subunits were 6.8 (ASIC1a), 6.2 (ASIC1b), 4.9 (ASIC2a) and 6.6 (ASIC3) [269, 270].

Activation and desensitization of ASICS occurs in response to extracellular acidification. The channels formed by the ASIC proteins are proton-gated cation channels, opening rapidly in the presence of extracellular acid. Opening of the ion pore could be accomplished by a concerted global rotation of the TM2 helices. Their fundamental role appears to be acid transduction, converting an acidic extracellular environment into a cellular signaling event, by allowing for the conduction of cations into the cell [271].

The underlying mechanism coupling proton binding in the extracellular region to pore gating is unknown, nevertheless the role of any ion channel in a disease process can often be distilled down into inappropriately increased or decreased activity. 

In the peripheral nervous system (PNS), ASIC function usually as sensory receptors, for example in specialized nerve endings. They were found in neurons innervating skin [265, 273, 274], heart [275], intestines – especially ASIC3 [276], muscles [277], bones [278], eyes [279, 280], ears [281] and taste buds [282].

There are two suppositions about localization of ASICs in the brain. One of them locate ASICs at synapses but most likely they are situated in the post-synaptic membrane or throughout the neuron [283].

ASICs are associated with four genes (ASIC1, -2, -3, and -4) that encode for 6 subunits: ASIC1a, ASIC1b, ASIC2a, ASIC2b, ASIC3 and ASIC4 [260]. When expressed in heterologous systems, all subunits except ASIC2b and ASIC4 can form functional homomultimeric channels with distinct electrophysiological and pharmacological properties. When co-expressed, the heteromultimeric channels may demonstrate characteristics dramatically different from their homomeric counterparts [284]. ASIC1 and -2; both have alternative splice transcripts: ASIC1a, -1b, -2a, and -2b. ASIC4 has not been shown to produce or modulate proton-evoked current and remains the least understood subunit.

The pluralism of nomenclature introduced by different groups of researchers can be confusing and should be systematized (Table **1**). The terms involving ‘acid-sensing ionic channel’ (ASIC) could seem to be most advantageous. Sometimes, names indicate also the tissue specificity (*e.g.* the dorsal root ASIC, DRASIC) but it is still unclear whether H^+^ are, in fact, natural ligands of these peculiar receptor channels. ASICs belong to the family of amiloride-sensitive ENaC/DEG receptor channels, which determines their synonyms as ‘X-NaC’. The ‘NaC’ in this abbreviation means ‘sodium channel’ and ‘X’ is related to a basic feature of the protein (DEG – degenerin, B – brain, L – liver, I – intestine refers to the members of this family that are degenerin proteins). It is easy to anticipate that the ASIC nomenclature is still not final [259]. 

### Anxiety

The primary function of the nervous system is to generate flexible behavior in a changing environment. The acid-sensing ion channel 1a (ASIC1a) is abundantly expressed in the amygdala complex and other brain regions associated with fear [283, 286]. Other work has shown that the amygdala, a collection of nuclei buried in the temporal lobe of the brain, is essential for both innate and learned fear in rodents and humans [300]. In 2009, group of Ziemann proved that ASIC1a in amygdala take part in the appearance of fear by detecting a decrease in extracellular pH and triggering cationic currents. They report that inhalation of carbon dioxide (CO_2_) – simulating suffocation - decreases the pH in the amygdala and yields freezing behavior in mice. Moreover, genetic deletion or pharmacological disruption of ASIC1a channel reduces fear associated with CO_2_ inhalation. This finding provides new insight into the mechanisms by which amygdala neurons detect threat and suggests a new role for amygdale chemosensation in learned fear [287]. 

### Depression

Depression remains one of the most disabling medical diseases but the molecular pathways underlying depression are poorly understood, and existing treatments are too often ineffective. One of the brain part, the amygdala play a central role in mood regulation. ASIC1a is expressed widely in the central and peripheral nervous systems and is robustly expressed in structures associated with mood including the amygdale. ASIC1a is required for acid-evoked currents in central neurons, where it contributes to synaptic plasticity and in the regulation of dendritic spines. Some preclinical screenings showed that genetically disrupting ASIC1a in mice produced antidepressant-like effects in the forced swim test, the tail suspension test, and following unpredictable mild stress. Pharmacologically inhibiting ASIC1a also had antidepressant-like effects in the forced swim test. The effects of ASIC1a disruption in the forced swim test were independent of and additive to those of several commonly used antidepressants [289].

### Epilepsy

There are some interesting insights in how ASICs interact epilepsy and seizures. One of them showed that acid-sensing ion channel 1a (ASIC1a) activation enhances neuronal excitability in the hippocampus and neocortex, indicating that ASIC1a might play a role in the generation and maintenance of epileptic seizures [292]. Other publication mentioned that ASICs’ inhibitor – amiloride neither altered the threshold for electroconvulsions, nor protected the animals against MES-induced seizures in mice but amiloride administered 75 and 100 mg/kg, *i.p.*, 120 min prior to the test significantly enhanced the anticonvulsant effects of carbamazepine, oxcarbamazepine and phenobarbital, by reducing their ED_50_ values in the MES test [291]. On the other hand, there was also established that seizures reduce extracellular pH. Extracellular acidosis, in turn, activates ASIC1a, which terminates seizure activity [290].

### Glioma

Gliomas are primary brain tumors with a complex biology characterized by antigenic and genomic heterogeneity and a propensity for invasion into normal brain tissue. High grade glioma cells possess a voltage-independent, amiloride-inhibitable, inward Na-current. This current does not exist in normal astrocytes or low grade tumor cells. Inhibition of this conductance decreases glioma growth and cell migration making it a potential therapeutic target. Some results have shown that the acid-sensing ion channels (ASICs especially ASIC2), are part of this current pathway [293]. 

### Huntington’s Disease

Huntington's disease (HD) is a neurodegenerative genetic disorder that affects muscle coordination and leads to cognitive decline and psychiatric problems. Some HD models have been studied, where amiloride derivative benzamil (Ben), a chemical agent used to rescue acid-sensing ion channel (ASIC)-dependent acidotoxicity, was examined whether chronic acidosis is an important part of the HD pathomechanism and whether these drugs could be used as novel therapeutic agents. Ben markedly reduced the huntingtin-polyglutamine (htt-polyQ) aggregation in an inducible cellular system, and the therapeutic value of Ben was successfully recapitulated in the animal model of HD [294].

### Multiple Sclerosis

Multiple sclerosis is one of the most common causes of progressive disability affecting young people. Multiple sclerosis is a neuroinflammatory disease associated with axonal degeneration. The neuronally expressed, proton-gated acid-sensing ion channel-1 (ASIC1) is permeable to Na^+^ and Ca^2+^, and excessive accumulation of these ions is associated with axonal degeneration. ASIC1 are hypothesized to contributes to axonal degeneration in inflammatory lesions of the central nervous system (CNS). In this context, ASIC1’s blockers could provide neuroprotection in multiple sclerosis [295]. According to some researches, blocking ASIC1 with amiloride protected both myelin and neurons from damage in the acute model, and when given either at disease onset or, more clinically relevant, at first relapse, ameliorated disability in mice with chronic-relapsing experimental autoimmune encephalomyelitis [288].

### Pain

Tissue acidosis is a common feature of many painful conditions. Protons are indeed among the first factors released by injured tissues, inducing a local pH decreasing that depolarizes peripheral free terminals of nociceptors and leads to pain. ASICs are excitatory cation channels directly gated by extracellular protons that are expressed in the nervous system. At the peripheral level, ASIC3 is important for inflammatory pain. At the central level, ASIC1a is largely expressed in spinal cord neurons where it has been proposed to participate in the processing of noxious stimuli and in central sensitization. Blocking ASIC1a in the spinal cord also produces a potent analgesia. Targeting ASIC channels at different levels of the nervous system could therefore be an interesting strategy for the relief of pain [272]. Pharmacological block of ASIC1a activity by the PcTx1 injected intrathecally produces strong analgesic effects in several rodent models of acute and chronic pain. The genetic knockdown of ASIC1a expression by intrathecal delivery of antisense oligonucleotides produces similar analgesic effects [296, 297]. 

### Amiloride

In the great majority of studies, amiloride has been used to block ASIC, but is a weak blocker, especially in contrast to its affinity for ENaCs (10–100-fold higher).

### Aminoglycosides (AGs)

Aminoglycosides (AGs) are a group of antibiotics whose structure is based on aminocyclitol rings interconnected by glycosidic bonds. Some studies showed that AGs may constitute a valuable tool for the modulation of ASICs, and may be a feasible starting point for the development of new molecules structurally related to the AGs but with a greater affinity, specificity, and selectivity for the different ASIC subunits [306].

### APETx2

APETx2 is a novel peptide toxin isolated from sea anemone venom, which selectively inhibits homomeric ASIC3 channels as well as the ASIC1+3, ASIC1b+3, and ASIC2b+3 heteromers. APETx2 is a basic peptide (pI = 9.59) of 42 amino acids, cross-linked by three disulfide bridges. The discovery of a toxin with ASIC3-blocking activity is of major significance given the substantial amount of evidence now implicating this channel in the transduction of acid-induced pain and hyperalgesia [303].

### A-317567

A-317567 is a novel non-amiloride ASIC blocker with distinct mode of action and improved potency both *in vitro* and *in vivo* over amiloride. A-317567 blockes ASIC-like currents in cultured dorsal root ganglion neurons [301] and cultured cortical neurons [289]. While further evaluation of the mechanism of action of A-317567 is required to fully understand its mode of action, this compound will provide a new tool for investigating ASIC biology, physiology, and pathophysiology [301].

### Diaminazene

**Fig. (55):**
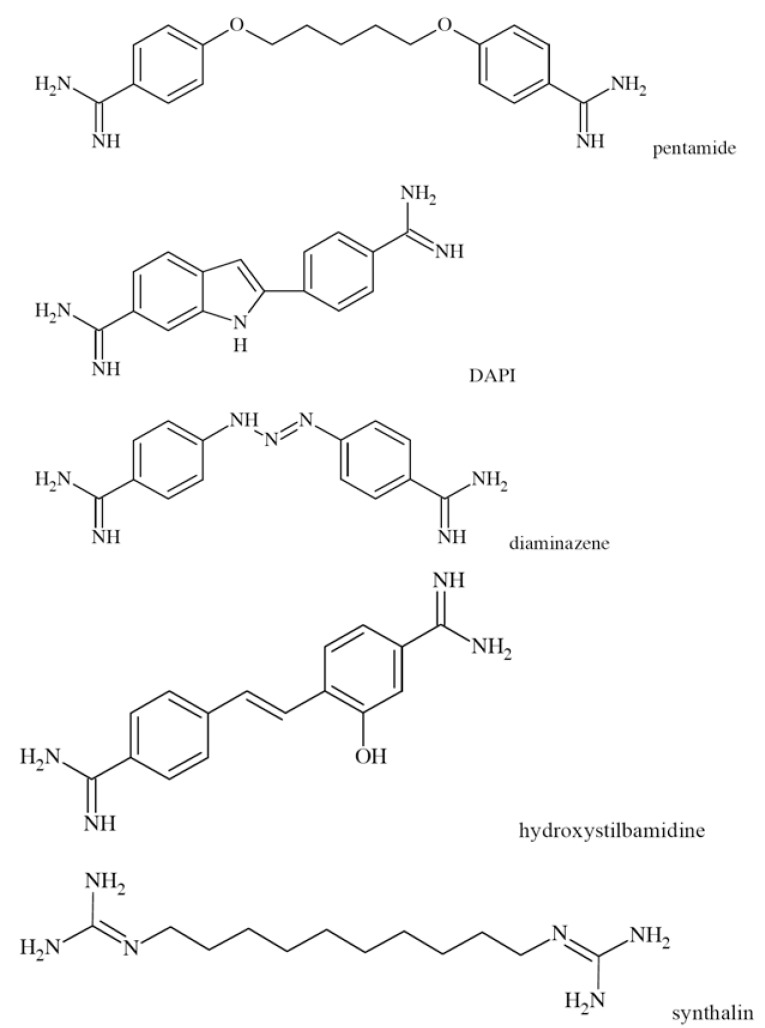
Chemical structures of aromatic diamidines [305].

Aromatic diamidines (Fig. **55**) are DNA-binding agents and have long been used in the treatment of leishmaniasis, trypanosomiasis, pneumocystis pneumonia and babesiosis. Moreover, some aromatic diamidines are used as skin-care and baby products and others have potential to suppress tumor growth or to treat malaria. The application of aromatic diamidines is not limited to anti-parasitics. Recently, it was found that aromatic diamidines such as pentamidine, hydroxystilbamidine, and diminazene potently block ASICs [305]. Each of these compounds inhibited ASIC currents, indicating that the linker in each of these diamidines can be variable, while the amidine group linked with an aromatic moiety appears to be essential. Among them, diminazene blocks ASICs with an IC_50_ of 0.3 μM, being the most potent small molecule inhibitor of ASICs. Moreover, diminazene inhibits sensory neuron-specific ASIC1b more strongly than any other ASIC subunits. 3μM diminazene almost completely blocks ASIC1b [311].

### Nafamostat

Nafamostat (Futhan, FUT-175) is a synthetic, competitive, reversible serineprotease inhibitor. Therefore, nafamostat functions as an anti-inflammation and anti-coagulation agent. It is clinically used, mainly in the East Asia, for the treatment of disseminated intravascular coagulation, acute pancreatitis, and as an anticoagulant in extracorporeal circulation. Nafamostat, like aromatic diamidines, is also a highly polarized, linear di-cationic agent governed by an amidine group at one terminus and a guanidine group at the other. Recently, nafamostat blocks ASICs heterogeneously expressed in *Xenopus *oocytes with an IC_50_ of 2–70 μM depending on the ASIC subtype [307].

### Nonsteroid Anti-inflammatory Drugs (NSAIDs)

**Fig. (57):**
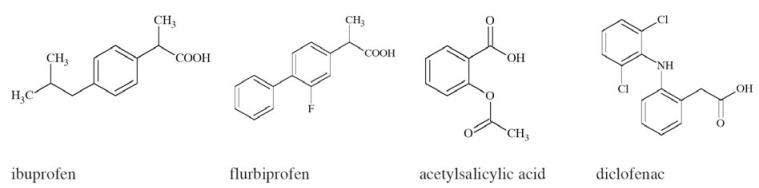
Chemical structures of some NSAIDs.

Nonsteroid anti-inflammatory drugs (NSAIDs) (Fig. **(57**) are major drugs against inflammation and pain. They are well known inhibitors of cyclooxygenases (COXs). However, many studies indicate that they may also act on other targets such as ASICs. There were proposed that the two effects, i.e., direct channel block and inhibition of inflammation-induced ASIC expression, play an important role in the antinociceptive effects of NSAIDs in addition to their well known effects on COXs and more particularly in case of inflammation [304].

### PcTx1

The first selective inhibitor of ASIC1a was Psalmo-toxin 1 (PcTx1). It was purified from the venom of the tarantula *Psalmopoeus cambridge* [308]. PcTx1 is a 40 amino acid peptide crosslinked by three disulfide bridges, it has a molecular mass of 4689.40 Da. 

**Fig. (58):**
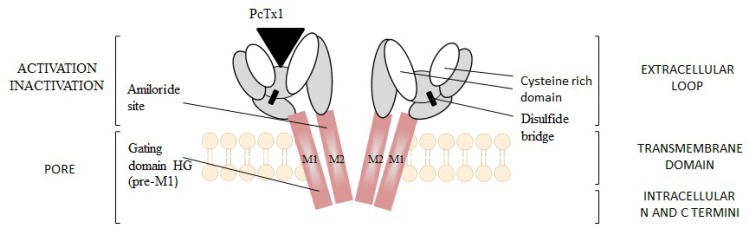
PcTx1 binding site on ASIC1a channel.

PcTx1 inhibit similarly homomeric ASIC1a channels in various cell expression systems with a very high affinity (see Table **9**). PcTx1 inhibits ASIC1a by increasing its apparent affinity for H^+^. PcTx1 affinity for ASIC1a depends on extracellular pH and is the highest in pH=7.4. However, the mechanism by which PcTx1 increases the apparent H^+^ affinity remains unclear [312]. PcTx1 also interacts with ASIC1b, a splice variant of ASIC1a. However, PcTx1 does not inhibit ASIC1b but promotes its opening; under slightly acidic conditions, PcTx1 behaves like an agonist for ASIC1b [313]. At the molecular level, it was suggested that the ion pore structure, constituted by trans-membrane M1 and M2 segments and intracellular pre-M1 and post-M2 regions (Fig. **(58**), are not directly involved in either the PcTx1 binding site or in its mechanism of inhibition. Amiloride, a known pore blocker of ENaC/DEG/ASIC channels, does not inhibit PcTx1 binding, which also supports this conclusion. PcTx1 binds principally on both cysteine-rich domains I and II (CRDI and CRDII) of the extracellular loop; the post-M1 and pre-M2 regions, although not involved in the binding site, are crucial for the ability of PcTx1 to inhibit ASIC1a current by modification of channel gating; the linker domain between CRDI and CRDII is important for their correct spatial positioning to form the PcTx1 binding site [310]. 

## 7 TRANSIENT RECEPTOR POTENTIAL (TRP) CATION CHANNELS

The superfamily of transient receptor potential (TRP) cation channels function as cellular sensors of various extra- and intracellular environmental changes. The first member of TRP channels was identifed in 1969 (by Cosens and Manning) in *Drosophila *mutants that displayed a transient response to light [314]. The family of transient receptor potential canonical (TRPC) proteins was the first group of TRP homologs cloned in mammals after discovery of the TRP protein in *Drosophila* [315]. 

The TRP channels comprises of five families (TRPA, TRPC, TRPM, TRPN and TRPV) and two subfamilies (TRPML and TRPP), which share several key properties: they are nonselective cation channels and most have six transmembrane domains [316, 317]. They are also divided into subtypes (Table **10**) [318].

**Fig. (59):**
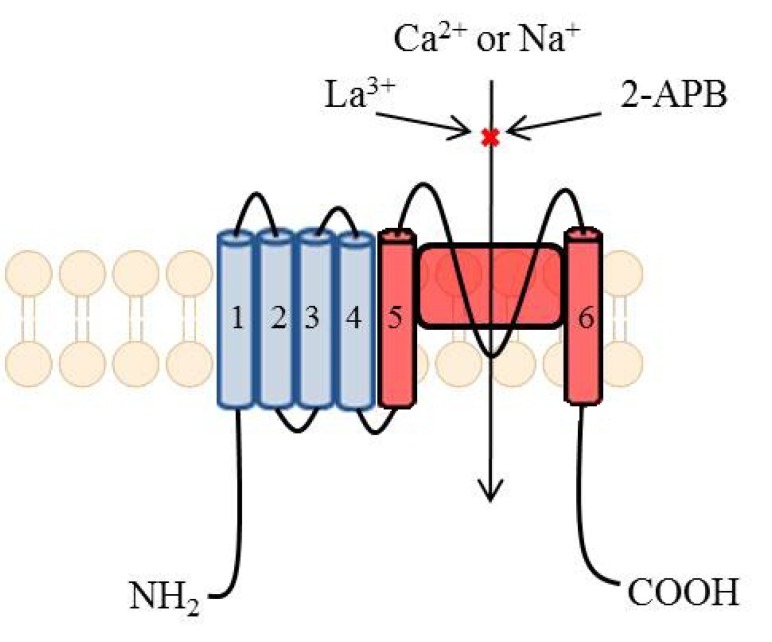
Schematic structure of TRP channels [320].

It was determined that, TRPs are tetrameric cation channels [319]. Each channel subunit contains six transmembrane segments (S1–S6) and a pore-forming region formed by a loop between the S5–S6 segments. Both the amino (N) terminus and the carboxyl (C) terminus are located intracellularly. Most of the TRP channels have an N-terminal ankyrin repeating domain and a C-terminal TRP domain. The long N and C termini of TRPs also contain several additional regulatory domains which are relatively conserved in most of the TRPs (Fig. **(59**) [314].

TRPA (ankyrin) family has one member: TRPA1 which contributes to the detection of intense mechanical stimuli. It has a characteristic large number of ankyrin protein repeats, thought to act as a spring and intracellular anchor, which may contribute to mechanosensory transduction. It responds to a range of environmental irritants e.g. formaline, extracts of mustard, cinnamon, garlic, oregano [321-323]. TRPA1 is localised in gastro-oesophageal vagal afferents, splanchnic colonic afferents and pelvic colonic afferents [316]. 

There are seven members in this family (TRPC1–7). The TRPC channels formed by homo- or heteromeric TRPC proteins are Ca^2+^-permeable nonselective cation channels. According to the similarity in amino acid sequence, the mammalian TRPCs can be classified into four subgroups: TRPC1, TRPC2, TRPC3/6/7, and TRPC4/5, while the TRPC2 is a pseudogene in primates. The TRPC proteins have six transmembrane domains, and both N- and C-termini of these proteins are intracellular, suggesting that these channels can be regulated by intra cellular signaling molecules [324]. These channels can be activated in various cell types by G-protein-coupled receptors (GPCR) and receptor tyrosine kinases (RTK) through a phospholipase C (PLC)-dependent mechanism. Therefore, TRPC channels may act as a sensor for environmental cues. In mammals, they were found in brain, heart, testis, ovaries, lung, retina, endothelia and adrenal glands. They partake in multiple physiological functions such as neuronal excitability in Purkinje cells, acrosome reaction in sperm cells, pheromone response, vasorelaxation and neurotransmitter release. They represent an important Ca^2+^ source for cells and have also been associated with Ca^2+ ^oscillations [325].

TRPM subunits are localized in dorsal root ganglion neurons, taste buds, gut epithelium, brain, kidney, heart, prostate, intestine, liver and lung [325, 326]. TRPM family is now widely researched because it mediates the cooling and soothing sensation of *e.g.* menthol and eucalyptol. In this context, TRPM8 may be critical in alleviating pain [316]. 

The TRPN family has not been detected in mammals, but is present in *Drosophila*, zebrafish and worm. It has been proposed to be sensitive to mechanical stimuli and is expressed in mechanosensory neurons of the ear and eye [327].

The TRPV, also called vanilloid family, is probably the best known subfamily of TRP channels, notably TRPV1 which is activated by the vanilloid chilli extract capsaicin. They are localized in neurons innervating gut, colon, stomach, skin, urinary bladder, spleen, liver, placenta, kidney, lung and testis [317, 325, 316].

The polycystin, or TRPP family has four members, TRPP1, 2, 3 and 5. Their name is derived from an association between a TRPP mutation and polycystic kidney disease. They play roles in developmental guidance and ciliary function. They recently received interest in connection with a role in acid sensing [316].

The mucolipin, or TRPML family has three members, one of which is associated with mucolipidosis. They have an intracellular function putatively in vesicle formation [316]. 

### Alzheimer’s Disease

Cognitive impairment and emotional disturbances in Alzheimer's disease (AD) result from the degeneration of synapses and neuronal death in the limbic system and associated regions of the cerebral cortex. Some indications suggest a role for TRPC6 in Alzheimer’s disease. Mutations in the presenilin genes are linked to the development of early-onset Alzheimer’s and transient expression of presenilin mutants (N141I, M239V) along with TRPC6 in human embryonic kidney - 293 cells results in a strong inhibition of agonist (1-oleoyl-2-acetyl-sn-glycerol (OAG) and angiotensin II (Ang II)) induced Ca^2+^ entry. Interestingly, the loss of function PS mutant D263A augments the activity of TRPC6 [328, 329].

### Bipolar Disorder

The pathophysiology of bipolar disorder has been connected to a variety of TRP channels. Findings of Goedding and coworkers implicate the calcium-permeable TRPM2 and canonical TRPC3 in the pathogenesis of bipolar disorder (BD). These channels are involved in calcium and oxidative stress signaling, both of which are disrupted in BD. Thus, authors sought to determine if these channels were differentially affected by oxidative stress in cell lines of BD patient origin using B lymphoblast cell lines from bipolar I disorder (BD-I) patients, the oxidative stressor and TRPM2 and TRPC3 respective activators H_2_O_2 _and OAG. Data support an important role for TRPM2 and TRPC3 in sensing and responding to oxidative stress and in transducing oxidative stress signaling to intracellular calcium homeostasis and cellular stress responses, all of which have been implicated in the pathophysiology of BD [330]. It is also worth mentioning in connection with depressive mental disorders that leptin, which is connected to TRPC1 (and probably also TRPC3), has antidepressant effects and that impaired leptin production contributes to depression-like phenotypes in a rat model [331].

### Ischemic Stroke

It is known that restoring extracellular Ca^2+^ following a period of low Ca^2+^ concentrations paradoxically causes an increase in intracellular Ca^2+^ levels that can lead to cell death. The mystery of this ‘Ca^2+^ paradox’ is made more intriguing by observations that lowering concentrations of extracellular Ca^2+^ and/or Mg^2+^ paradoxically enhances the entry of Ca^2+^ into hippocampal neurons. Some research concluded that extracellular Ca^2+^ and Mg^2+^ are important regulators of intracellular Ca^2+^, through their modulation of TRPM7 and potentially other non-selective cation channels such as TRPM2 [332, 333].

### Parkinson’s Disease

1-Methy-4-phenylpyridium (MPP1) induces Parkinson disease-like syndromes. When applied to human dopaminergic SH-SY5Y neuroblastoma cells, MPP1 causes decreased expression and plasma membrane localization of TRPC1. In an opposite manner, TRPC1 overexpression reduces the neurotoxicity of MPP1. Some studies showed that activation of TRPC1 by thapsigargin or carbachol decreased MPP1 neurotoxicity Thus TRPC1 may execute a neuroprotective role in dopaminergic neurons. Furthermore, TRPC1 may inhibit degenerative apoptotic signaling to provide neuroprotection against Parkinson’s disease-inducing agents [334, 335].

### Schizophrenia

Schizophrenia is a chronic, debilitating psychiatric disorder that afflicts people of all races and carries a life-time risk of the order of 1%. Schizophrenia is accompanied by morphological changes in the brain that include enlarged ventricles, a reduced cortical thickness, but an increased prefrontal neuron density. Patients also exhibit reduced pain sensitivity and a diminished niacin skin flare response. The latter symptoms hint towards a defect in TRPV1-expressing afferent nerve fibers. Direct evidence for links between schizophrenia and TRP channels is lacking. However, several aspects of the pathophysiology of the disorder point to a possible involvement of TRP channels. Links between TRP channels and schizophrenia with respect to neurodevelopment, dopaminergic and cannabinoid systems, thermoregulation, and sensory processes, are still discussed. Investigation of these links holds the prospect of a new understanding of schizophrenia with resultant therapeutic advances [336, 337].

### Traumatic Brain Injury (TBI)

Traumatic brain injury classically results from mechanical injury induced by physical impact or a rapid change in acceleration/deceleration of the head. TBI can quickly lead to dysfunction of the microvessel endothelial cells in brain, including disruption of blood–brain barrier (BBB). It is now evident that elevation of cytosolic calcium levels [Ca^2+^]i can compromise the BBB integrity, however the mechanism by which mechanical injury can produce a [Ca^2+^]i increase in brain endothelial cells is unclear. Ca^2+^ is a ubiquitous second messenger that plays key roles in the regulation of cellular processes such as gene expression, secretion and apoptosis. Ca^2+^ channels are of particular interest in cell proliferation because of the profound anti-proliferative effect seen when extracellular Ca^2+^ is removed. Evidence from studies of many cell types indicates that Ca^2+^ entry mechanisms have an essential role in this effect. The concentration of Ca^2+^ is carefully controlled through the regulation of a variety of membrane channels and pumps including TRP channels [338]. It was stated that blockade of TRPC1 and TRPP2 both by chemical inhibitors (amiloride, LOE908) and by siRNA knockdown assays directed at TRPC1 and TRPP2 expression abolished the injury transient [Ca^2+^]i increase. Taking into account these fact it can be stated that mechanical injury of brain endothelial cells induces a rapid influx of calcium, mediated by TRPC1 and TRPP2 channels [339].

## 8 GABA RECEPTORS

GABA (γ-aminobutyric acid) is the most abundant inhibitory neurotransmitter found in the mammalian CNS, where about 20% of all neurons are GABAergic [342]. GABA acts by binding to two different types of receptors, ionotropic receptors (GABA_A_ and GABA_c_) and metabotropic receptors (GABA_B_). Two GABA receptors subfamilies GABA_A_ and GABA_C_ differ in their ability to form endogenous heteromeric and homomeric receptors and in their physiological and pharmacological properties [343]. 

GABA_A_, as ligand-gated ion channels, facilitate rapid responses. Activation leads to two types of GABAergic inhibition: phasic and tonic inhibition. Classical phasic GABA_A_ receptor-mediated inhibition is produced by brief exposure of postsynaptic GABA_A_ receptors to high concentrations of GABA. On the other hand, tonic GABA_A_ receptor-mediated inhibition results from the activation of extrasynaptic receptors by low concentrations of ambient GABA [344]. In many regions of the brain GABAA receptor-mediated currents are dominated by tonic currents, which account for about 75%-90% of total inhibitory currents [345]. 

**Fig. (65):**
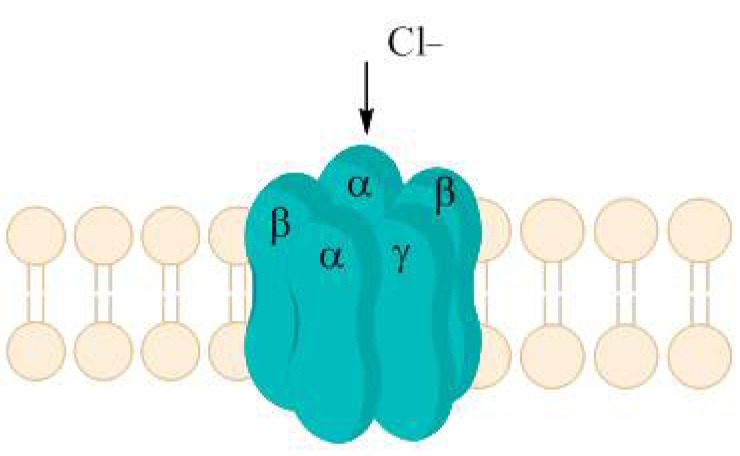
Schematic structure of GABA_A_ receptor.

GABA_A_ receptors (Fig. **(65**) are a heterogeneous family of ligand-gated ion channels that mediate inhibitory neurotransmission in the CNS. They are assembled from five subunits in a heteropentameric chloride ion channels. Various GABA_A_ receptor subunit subtypes as well as splice variants can be distinguished, such as α1-α6, β1-β3, γ1-γ3, δ, ε, π and θ. Each GABA_A_ receptor subunit is composed of a long extracellular N terminus, four transmembrane domains (M1-M4), one extracellular M2-3 loop, two intracellular loops (M1-2 and M3-4) and a short extracellular C terminus. 

αβγ and αβδ receptors are the principal isoforms present* in vivo* [346]. The most omnipresent and predominant synaptic receptor in the brain is α1β2γ2 isoform [347]. The αβδ GABA_A_ receptors are localized extra- or perisynaptically and mediate GABAergic tonic inhibition. In comparison with synaptically localized αβγ receptors, αβδ receptors are more sensitive to GABA, show relatively slower desensitization and display lower efficacy to GABA agonism [348]. A lot of studies have implicated the important role of tonic inhibition in neuronal excitability mediation under physiological conditions. 

Recent studies have demonstrated that defects in αβδ receptor function or tonic inhibition resulted in neurological disorders such as epilepsy [349, 350]. Studies regarding the genetics of human epilepsy demonstrated that various heritable mutations in GABA_A_ receptor subunits are associated with epilepsy [351]. It is well established that activation of GABA_A_ receptors plays an important role in sleep [352]. It was indicated that especially β3 GABA_A_ receptor subunit is involved in this condition [353]. 

Aside from epilepsy and sleep disturbances there are a number of pathological conditions for which GABA_A_ receptors represent vital therapeutic targets such as anxiety disorders, cognitive disorders, moods disorders and schizophrenia [354].

GABA_A_ receptor, apart from response to GABA, can be also allosterically regulated by an increasing number of agents such as benzodiazepines (BZs), barbiturates, anticonvulsants and anesthetics [355]. Three distinct binding sites present on GABA_A_ receptors were identified: GABA/ muscimol, t-butylbicyclophosphorothionate (TBPS)/picrotoxin, and benzodiazepine [356].

GABA_A_ receptors are omnipresent in the CNS. Thus, the current goal in neuropharmacology is to target drugs selectively to defined GABA_A_ receptor subtypes. This new strategy may help to improve the therapeutic spectrum of the presently available drugs and indicate new therapeutic indications. Additionally, such subtype-specific drugs are expected to display fewer concomitant unwanted effects, as they influence only a small population of GABA_A_ receptors. 

As the number of compounds interacting with GABA_A_ receptors is impressive a complete coverage of all of them within this review would be difficult. Then, only recently developed compounds showing efficacy at GABA_A_ receptors is presented. 

### SL651498

**Fig. (66):**
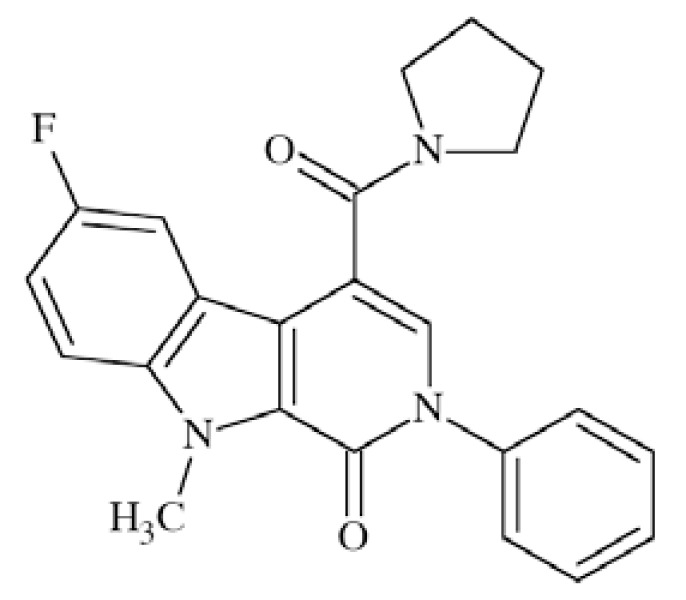
Chemical structure of SL651498.

SL651498 (Fig. **66**) a pyridoindole derivative is a full agonist at GABA_A_ receptors containing α2 and α3 subunits and a partial agonist at GABA_A_ receptors containing α1 and α5 subunits. The compound showed anxiolytic-like activity similar to that of diazepam (minimal effective dose (MED): 1-10 mg/kg, *i.p.*) in conflict models such as the elevated plus-maze, the light/dark test, and the defense test battery in rats and mice. Additionally, the study indicated that SL651498 produced ataxia, muscle weakness or sedation at doses much higher than those producing anxiolytic-like activity. No appearance of tolerance to its anticonvulsant effects or physical dependence was observed in mice [357]. SL651498 elicits anxiolytic-like effects similar to conventional BZs. The compound also fully induced muscle relaxation but in contrast to classical BZs produced minimal ataxia in monkeys [358]. The highest dose of SL651498 demonstrated slight effects on saccadic peak velocity (SPV) and smooth pursuit performance, albeit to a much lesser extent than lorazepam. Additionally, in contrast to lorazepam, none of the SL651498 doses influenced visual analogue scale alertness, body sway, attention, or memory tests [359]. 

### MRK-409

MRK-409 (Fig. **(37**) also known as MK-0343 was originally designed to be a less sedating anxiolytic, based on reduced efficacy at the α1 subtype and significant efficacy at α2 and α3 subtypes of the GABA_A_ receptor. The anxiolytic-like and sedative activity was observed at minimum effective doses corresponding to occupancies, depending on the model used, ranging from about 35% to 65% yet there were minimal overt signs of sedation at occupancies greater than 90% [360]. MK-0343 at 0.75 mg in humans was equipotent with lorazepam, on the other hand diminished effects on memory and postural stability were observed. 0.25 mg dose only influenced postural stability to a similar extent as MK-0343 0.75 mg [361]. Recent studies showed that treatment with MRK-409 leads to sedation in humans at a dose (2 mg) corresponding to the levels of occupancy considerably less than those predicted formerly in rodent models to be required for anxiolytic activity. It was concluded that the preclinical non-sedating anxiolytic profile of MRK-409 could not be translated into humans and further development of the compound was halted.

### BL-1020

**Fig. (68):**
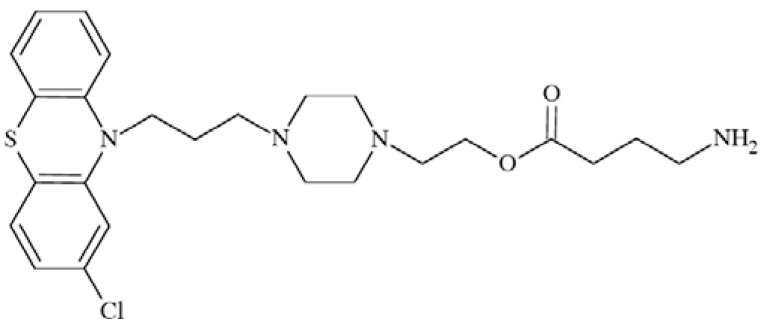
Chemical structure of BL-1020.

BL-1020 (Fig. **(68**) is a novel, orally bioavailable ester of perphenazine and GABA. Unlike first- and second-generation antipsychotics, it has agonist activity at GABA_A_. Compound’s efficacy in acute and subchronic schizophrenia rat models was evaluated by Geffen *et al.* [362]. The study showed that acute and subchronic treatment with BL-1020 antagonized amphetamine-induced hyperactivity, with significantly lower catalepsy and sedation compared to equimolar perphenazine. Phase-II, open-label study suggested that 20 to 40 mg/d of BL-1020 is associated with clinically relevant improvement of psychosis with no worsening of extrapyramidal symptoms (EPS) [363]. Fitzgerald [364] summarized clinical trials of BL-1020 and concluded that the compound was well tolerated in all trials conducted, and clinically meaningful improvements were demonstrated in phase II trials in patients with schizophrenia.

### L-838417

**Fig. (69):**
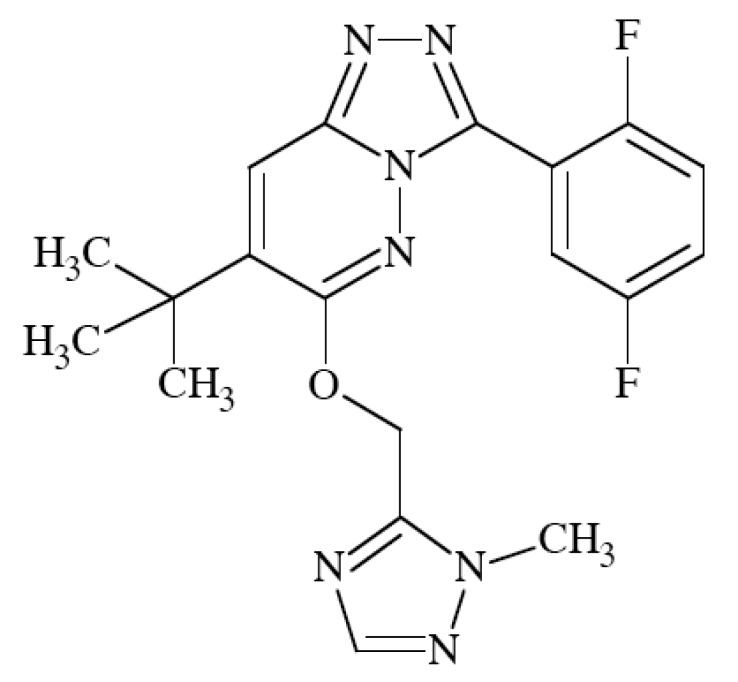
Chemical structure of L-838417.

L-838417 (Fig. **(69**) is the novel benzodiazepine site ligand, a partial agonist at GABAA receptors containing an α2, α3 or α5 subunit and an antagonist at α1 receptors, giving an anxiolytic profile devoid of sedation [365, 366]. It showed anxiolytic-like activity in wild-type rats as shown in the elevated plus-maze test and in a conditioned fear-potentiated startle protocol. Significant motor effects were not observed [367]. L-838417 was also anxiolytic in murine Vogel conflict test [368]. Additionally, the compound exhibited anticonvulsant activity [369]. 

### TPA-023

**Fig. (70):**
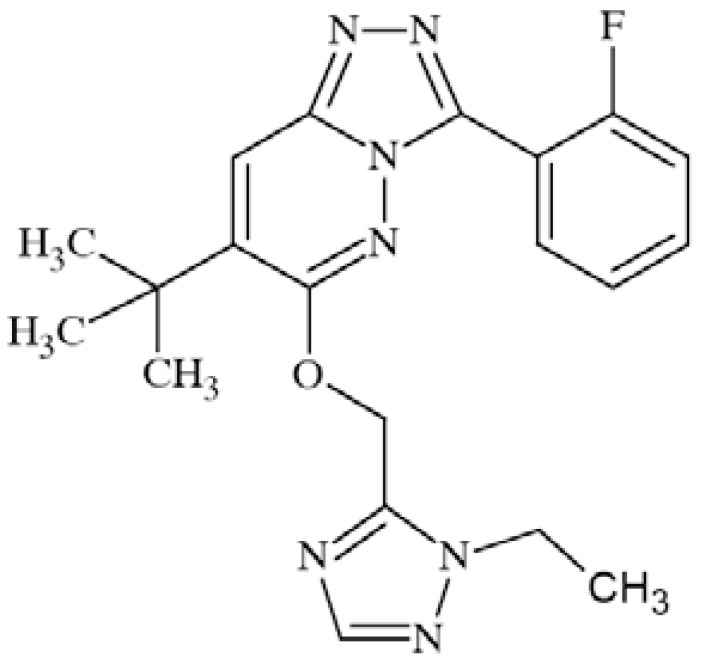
Chemical structure of TPA-023.

TPA-023 (Fig. **(70**) is an agonist selective for α2 and α3-containing GABA_A _receptors. In unconditioned (elevated plus maze) and conditioned (fear-potentiated startle and conditioned suppression of drinking) rat models of anxiety the compound showed anxiolytic-like activity with minimum effective doses (MED; 1–3 mg/kg) corresponding to 70 to 88% occupancy. No appreciable sedation was stated in a response sensitivity (chain-pulling) assay at a dose of 30 mg/kg. The compound elicited anxiolytic effect in the squirrel monkey conditioned emotional response assay (MED of 0.3 mg/kg), however, it was not able to produce any sedation in a lever-pressing test of sedation. Finally, TPA-023 showed anticonvulsant activity in a mouse pentylenetetrazole seizure model [370]. TPA-023 had significant dose dependent effects on saccadic peak velocity that approximated the effects of lorazepam [371]. However in contrast to lorazepam, TPA-023 had no detectable effects on saccadic latency or inaccuracy. In addition unlike lorazepam, TPA023 did not affect VAS alertness, memory or body sway. 

**Fig. (71):**
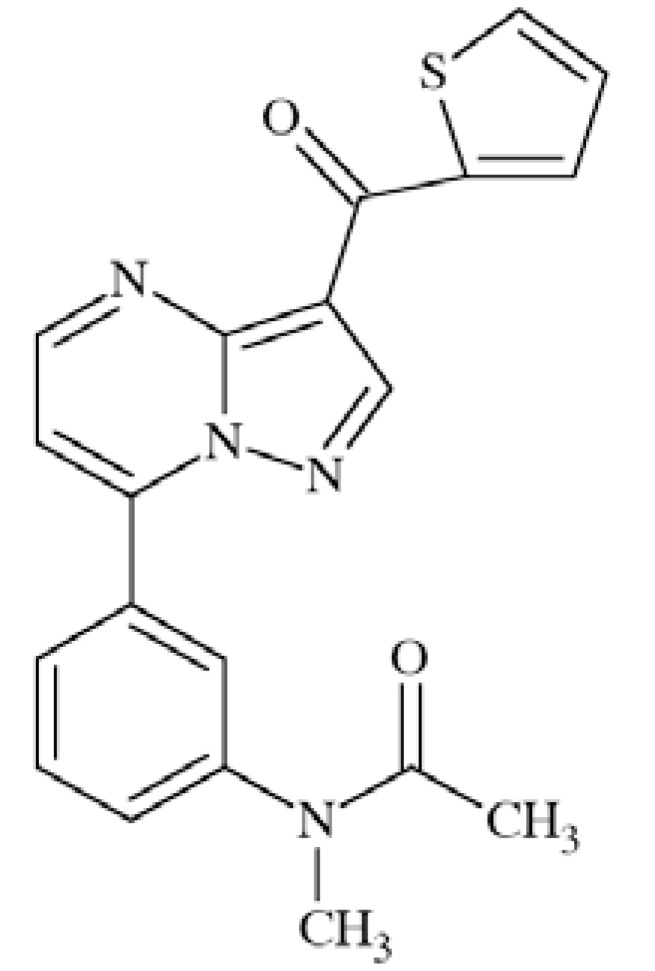
Chemical structure of indiplon.

Indiplon (Fig. **(71**) is a high-affinity allosteric potentiator of GABA_A_ responses that demonstrates preference for α1 subunit-containing GABA_A _receptors. The compound is studied for the treatment of insomnia. A double-blind, placebo-controlled trial investigated the efficacy and safety of modified-release indiplon in elderly patients with chronic insomnia. The study demonstrated that indiplon (15 mg), was well tolerated and significantly improved all patient-reported measures of sleep during the of treatment [372]. Long-term (3 months) nightly treatment with the drug (10 mg and 20 mg) has confirmed its efficacy in sleep onset, maintenance, and duration [373]. Indiplon’s activity was also confirmed in healthy volunteers in a model of transient insomnia [374]. 

### Y-23684

**Fig. (72):**
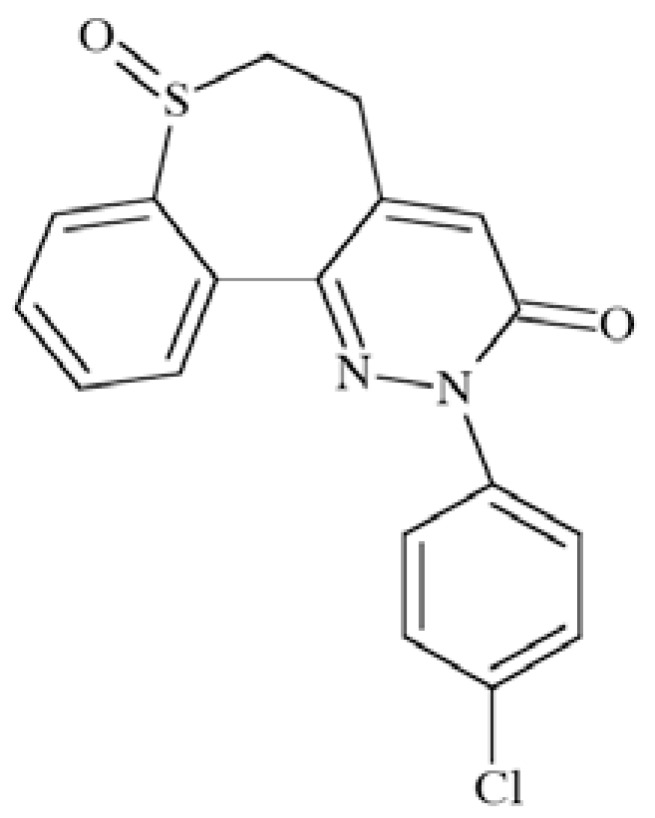
Chemical structure of Y-23684.

Y-23684 (Fig. **(72**) is pyridazinone derivative and selective partial agonist at BZ receptors. The compound is highly suggested to be a potent and selective anxiolytic agent with fewer side effects than classical BZ-anxiolytics. Studies comparing the pharmacological properties of Y-23684 with a classical benzodiazepine agonist diazepam demonstrated that in rat conflict tests (Geller-Seifter and water-lick tests), Y-23684 showed an antipunishment action at doses 2-4 times lower than diazepam and was at least as efficacious as diazepam in rodent models of anxiety (social interaction and elevated plus-maze tests). In a mouse model of anxiety (exploration (light/dark box) test), Y-23684 was two fold less effective as diazepam. In these paradigms, the compound demonstrated a selective anxiolytic activity over a wide dose-range without loss of efficacy and sedative action. Further studies with Y-23684 proved that the impairment of motor coordination (rotarod) and the enhancement of CNS depressants action by Y-23684 was weaker than that of diazepam [375]. Behavioral profiles in the mouse defence test battery confirmed Y-23684 anxiolytic potential that was comparable to that of BZs in the MDTB [376]. 

**Fig. (73):**
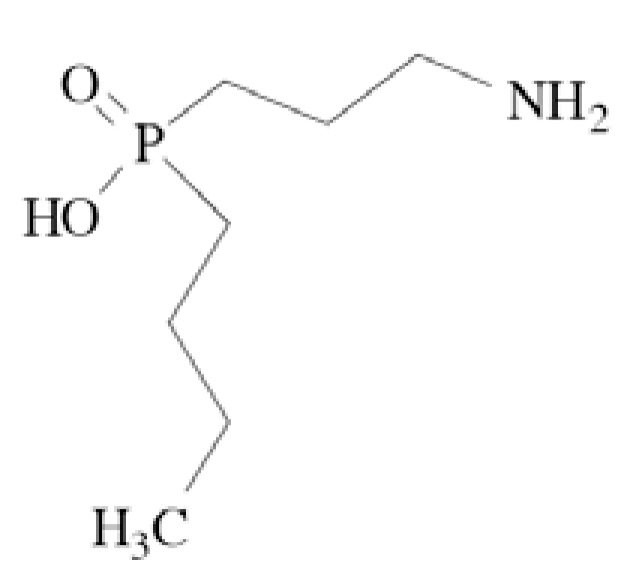
Chemical structure of CGP36742 or SGS742.

Most recently the development of selective brain-penetrating GABA_C_ receptor antagonists is of particular interest [377]. In this group of compounds there are conformationally restricted analogs of the orally active GABA_B/C _receptor antagonist (3-aminopropyl)-*n*-butylphosphinic acid (CGP36742 or SGS742) (Fig. **73**). These *cis*- and *trans*-(3-aminocyclopentanyl)butylphosphinic acids (*cis*- and *trans*-3-ACPBPA) are highly potent and selective for ρ1 GABA_C_ receptors, being greater than 100 times more potent at GABA_C_ than GABA_A _receptors and 50 times more potent at GABA_C_ than GABA_B_ receptors. These compounds showed potential use in sleep disorders, preventing the development of experimental myopia. Additionally, the substances enhanced learning and memory in rats [378, 379].

## 3 CONCLUSIONS

This review shows that there is a significant progress in understanding etiopathogenesis and molecular biology also in CNS diseases, which have been a major challenge for decades. However, drug discovery process seems too slow to provide specific ligands for ion channels/receptors subtypes. Many receptors subtypes lack a ligand, and if an effect of agonism/antagonism would be beneficial, this fact indicates a strong need for drug discovery in this field. 

Currently, many old drugs are tested for their potential use in diseases which are still defined as “unmet need”. This in turn implies side effects, which are rarely positive, as in the case of dihydropyridine derivatives binding to Ca_V_2.1 (P/Q) channels. In most cases of drugs the side effects are negative, and this together with effectiveness of old drugs in new diseases, is the greatest premise for drug development in novel subtypes of receptors. Nifedipine can serve as an example of an old drug, which on one hand could prevent from Parkinson’s disease, and on the other hand – it diminishes anticonvulsant activity of topiramate. 

Ion channels have been biological targets for the first synthesized drugs (*e.g.* phenytoin, carbamazepine, diazepam). Therefore, they seemed unattractive for some pharmaceutical companies, seeing no use in developing a “me-too” drug. However, this review shows that understanding of mechanisms and discovery of subtypes of ion channels may increase their involvement in therapies in the future (with some exceptions, of course, e.g. Ca_V_2.1 channels). CNS diseases, which are particularly hard to treat, are waiting to be managed, and we hope to have proved that this process is possible with use of novel ion channels ligands developed by the pharmaceutical market.

## Figures and Tables

**Fig. (1):**
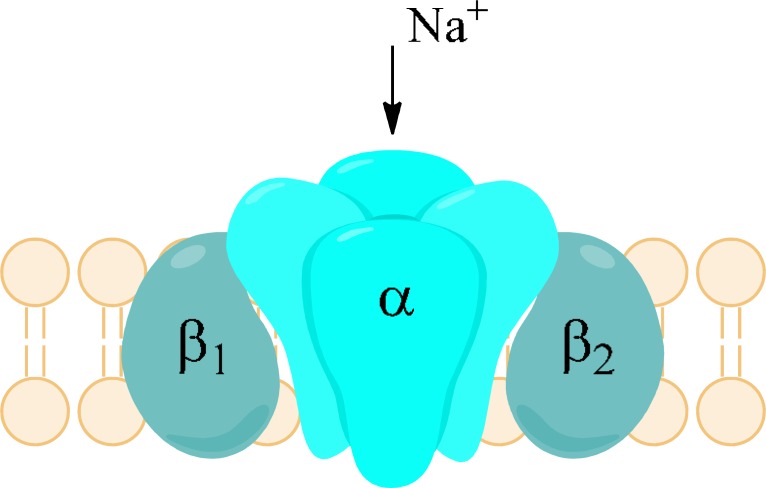
Schematic structure of VGSC.

**Fig. (7):**
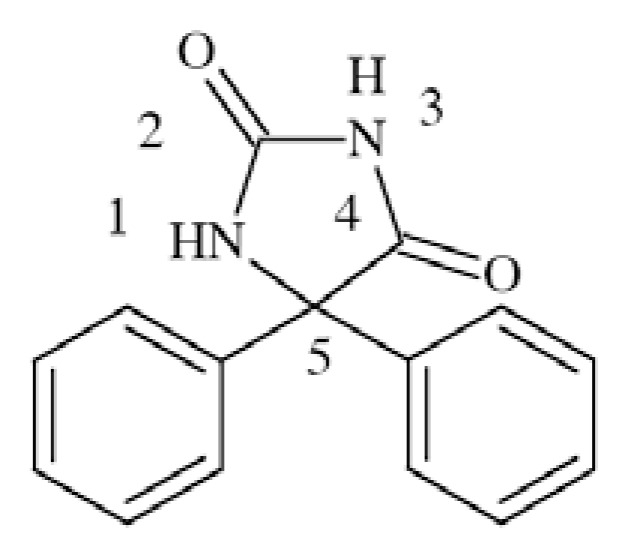
Chemical structure of phenytoin.

**Fig. (23):**
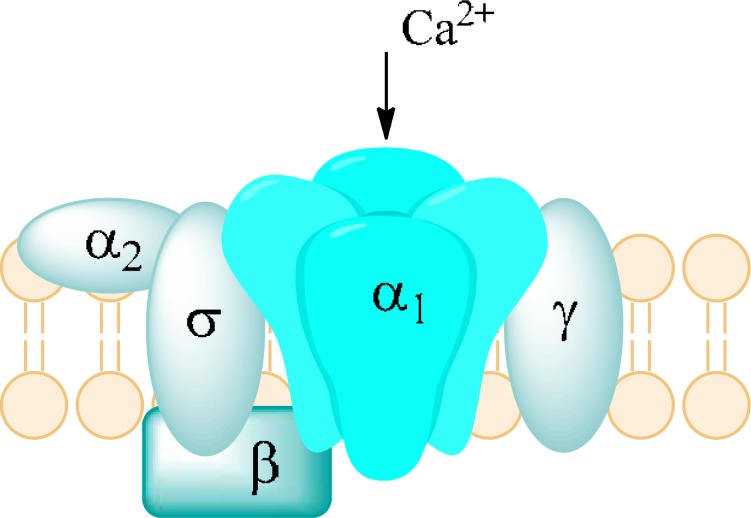
Schematic structure of calcium channels.

**Fig. (51):**
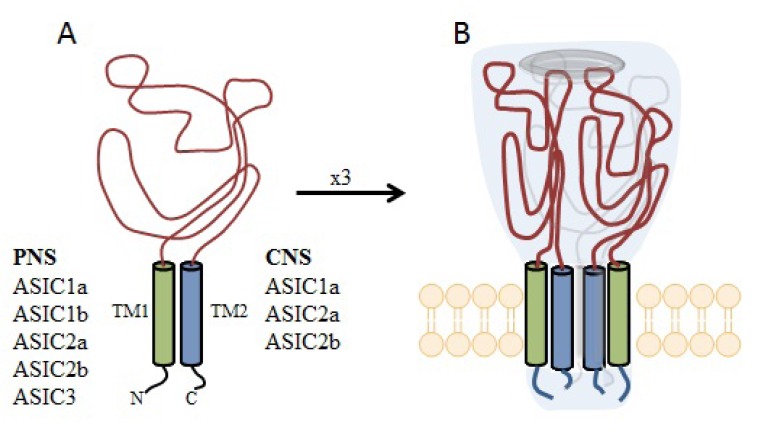
(**A**) Schematic concentration of an ASIC unit, (**B**) Trimeric organization of a functional ASIC channel (TM1 and TM2 tramsmembrane domains 1 and 2) [272].

**Fig. (52):**
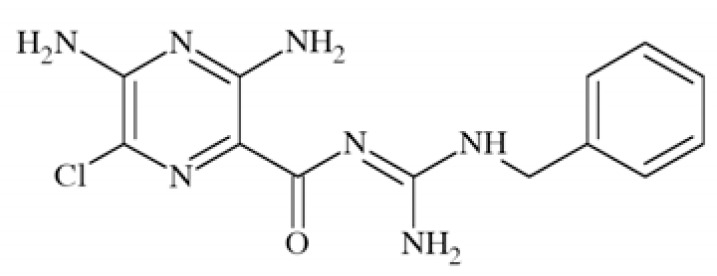
Chemical structure of benzamil.

**Fig. (53):**
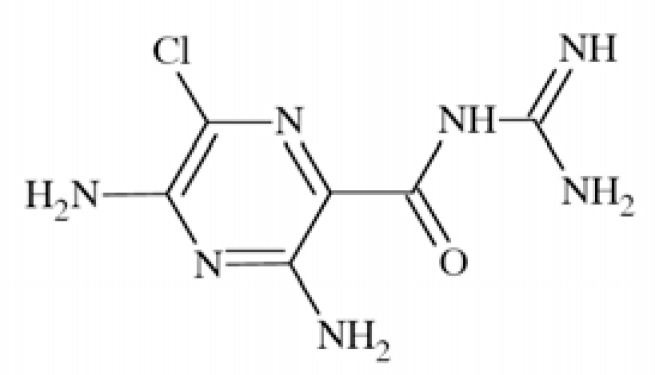
Chemical structure of amiloride.

**Fig. (54):**
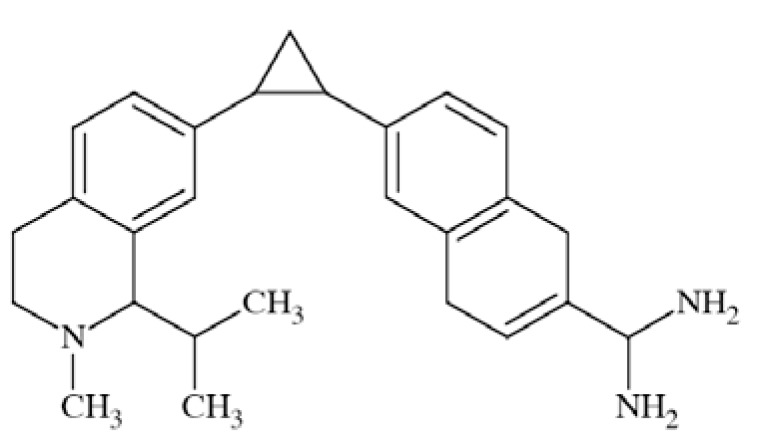
Chemical structure of A -317567.

**Fig. (56):**
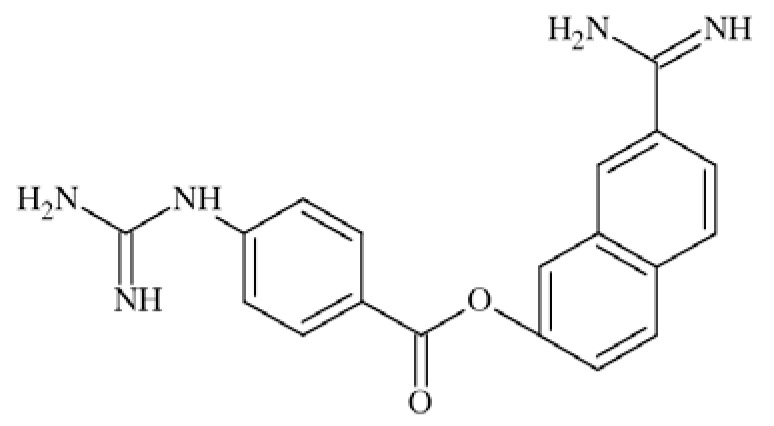
Chemical structure of nafamostat.

**Fig. (60):**
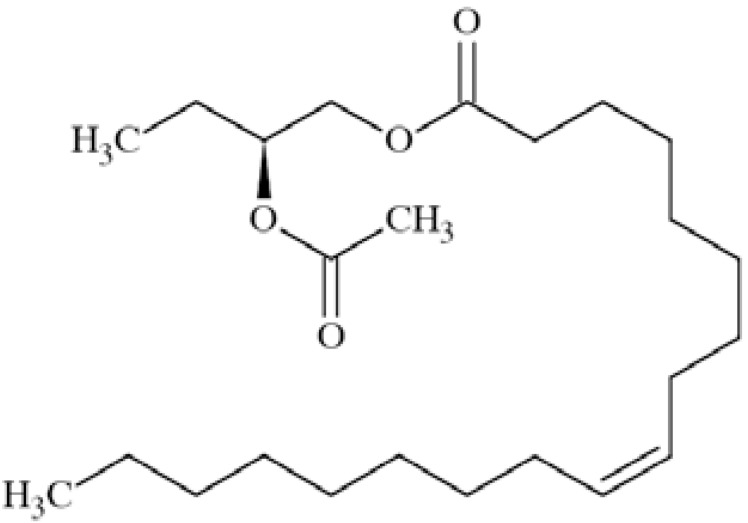
Chemical structure of OAG.

**Fig. (61):**
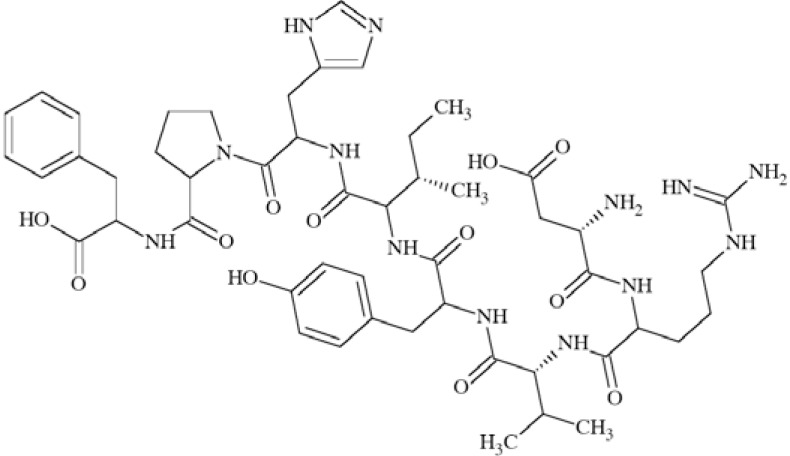
Chemical structure of angiotensin II.

**Fig. (62):**
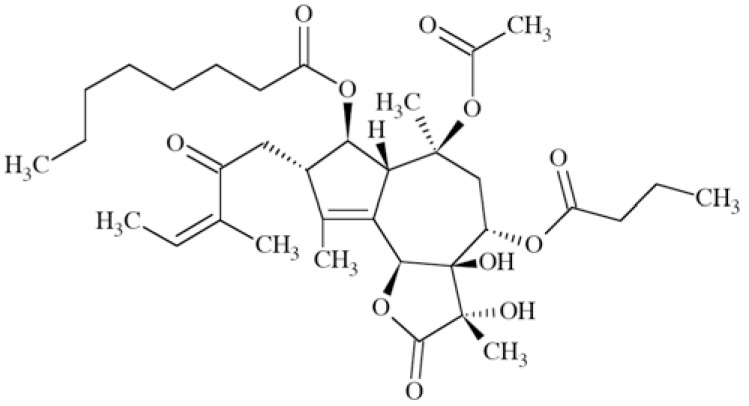
Chemical structure of thapsigargin.

**Fig. (63):**
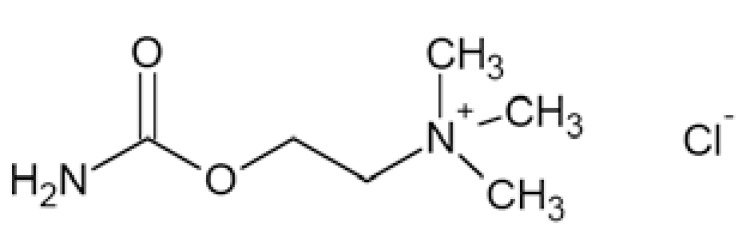
Chemical structure of carbachol.

**Fig. (64):**
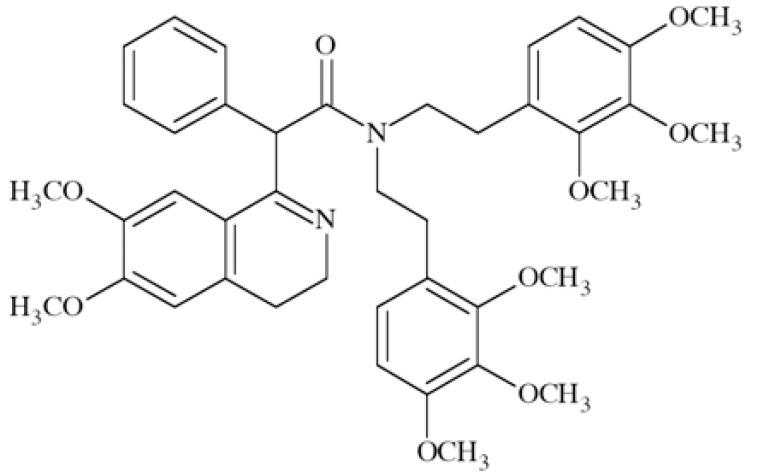
Chemical structure of pinokalant (LOE 908) [340, 341].

**Fig. (67):**
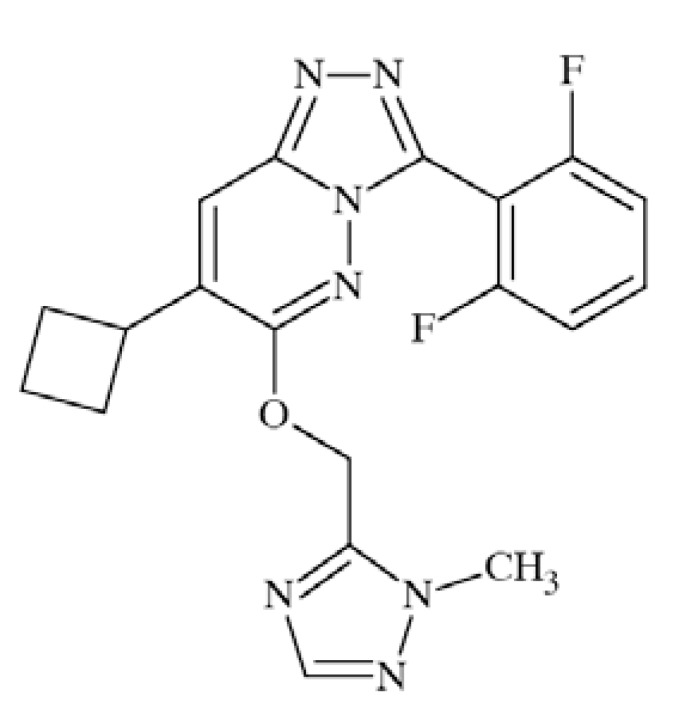
Chemical structure of MRK-409.

**Fig. (74):**
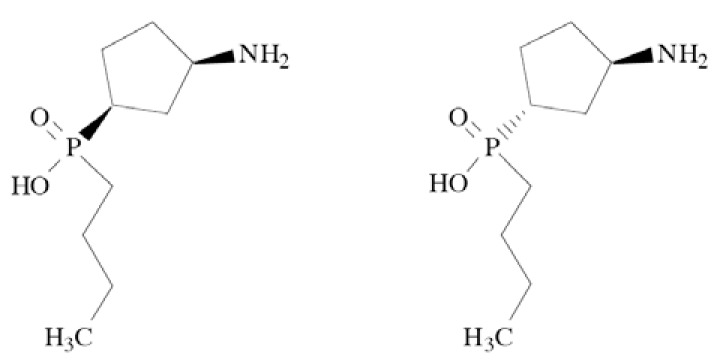
Chemical structures of *cis*-3-ACPBPA and *trans*-3- ACPBPA.

**Table 1.** Localization of Different Types of Voltage-gated Sodium Channels in Human Organism

Type of Sodium Channel α-Subunit	Human Gene	Localization	Disease
Na_V_1.1	SCN1A	Brain: neuronal somata, proximal dendrites [2, 12]	Epilepsy
Na_V_1.2	SCN2A	Brain: predominantly along axons: unmyelinated and myelinated [2], axon initial segments [13]	Epilepsy
Na_V_1.3	SCN3A	Brain: neuronal somata, proximal dendrites; [2, 12], upregulated expression in epileptic hippocampal neurons [14]	Epilepsy, Central neuropathic pain [15]
Na_V_1.4	SCN4A	Skeletal muscle [2]	
Na_V_1.5	SCN5A	Cardiac muscle; some neurons [2], microglial cells [16]	Atrial fibrillation, ventricular fibrillation [10]
Na_V_1.6	SCN8A	Brain: axon initial segments, nodes of Ranvier, neuronal somata, dendrites of projection neurons [2] proximal dendrites [12], unmyelinated and myelinated axons [17]	Ataxia
Na_V_1.7	SCN9A	Peripheral primary sensory neurons [2] Trigeminal ganglion neurons [18]	Neuropathic pain
Na_V_1.8	SCN10A		
Na_V_1.9	SCN11A		

**Table 2.** Epilepsy Syndromes As Result of Mutations in Sodium Channels Genes

Gene with Mutation	Epilepsy Syndrome

SCN1A	Severe myoclonic epilepsy of infancy (SMEI) [21]
	Generalized epilepsy with febrile seizures plus (GEFS+) [21]
	Benign febrile seizures (FS) [13]

SCN2A	Benign neonatal-infantile familial seizures (BNIFS) [11]
	Generalized epilepsy with febrile seizures plus (GEFS+) [11]
	Idiopathic generalized epilepsy [22]

SCN1B	Severe myoclonic epilepsy of infancy (SMEI) [10]
	Generalized epilepsy with febrile seizures plus (GEFS+) [10]
	Childhood absence epilepsy [22]
	Temporal lobe epilepsy [22]

**Table 3.** Binding Affinities, Pharmacological Properties and Proposed Biding Sites of LTG and Similar Compounds [54]

	K_D_ [µM] Rat Brain Na^+^ Channels (Na_V_1.2) Expressed in *Xenopus *oocytes in Inactivated State (- 50 mV)	Pharmacological Properties in Preclinical Evaluation	Position of Amino Acid Residues in IV- S6 Binding Site

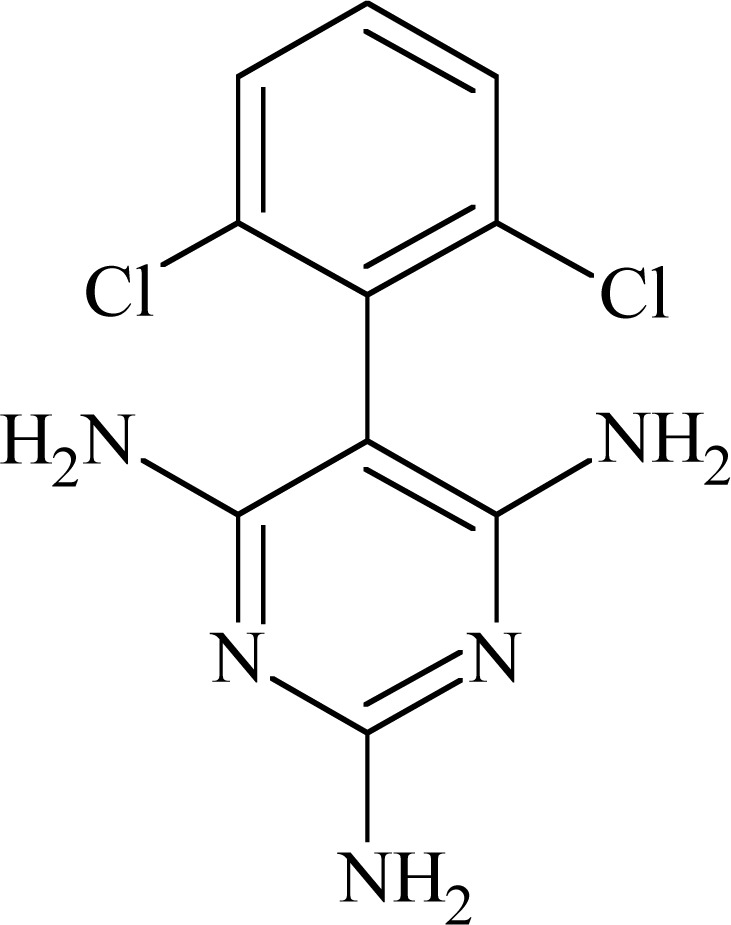	17.3	Analgesic but no anticonvulsant activity	1764 – strong interaction
			1771 – strong interaction
			1760 – weaker interaction

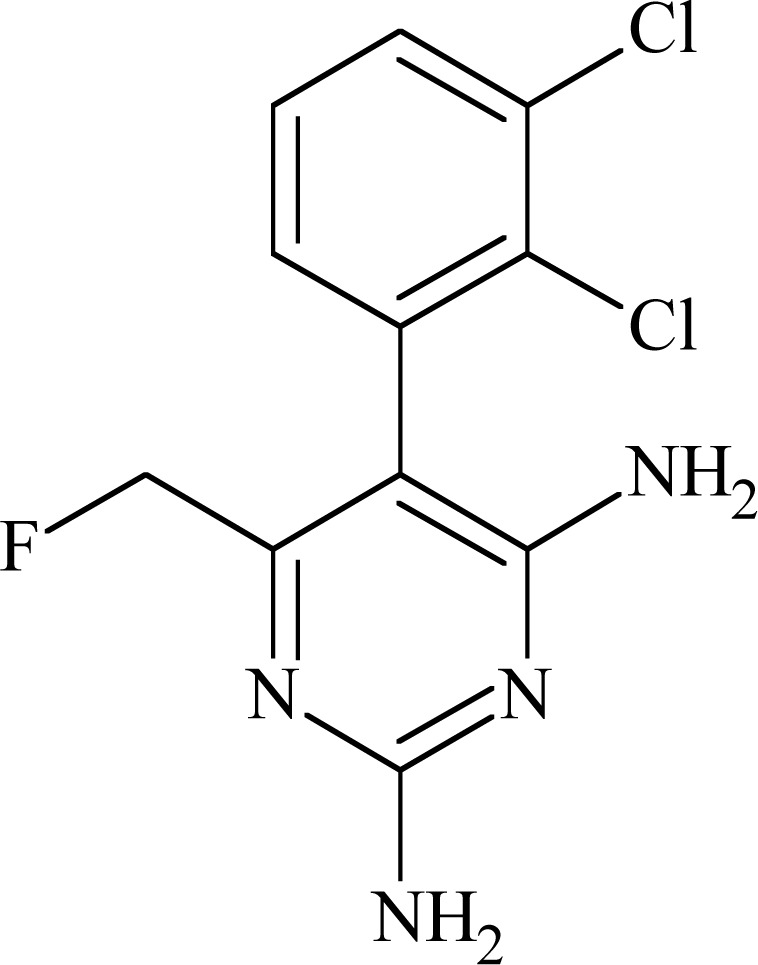	3.7	Analgesic and anticonvulsant activity	1764 – much stronger interaction;
			1771 – modest interaction;
			1760 weaker interaction

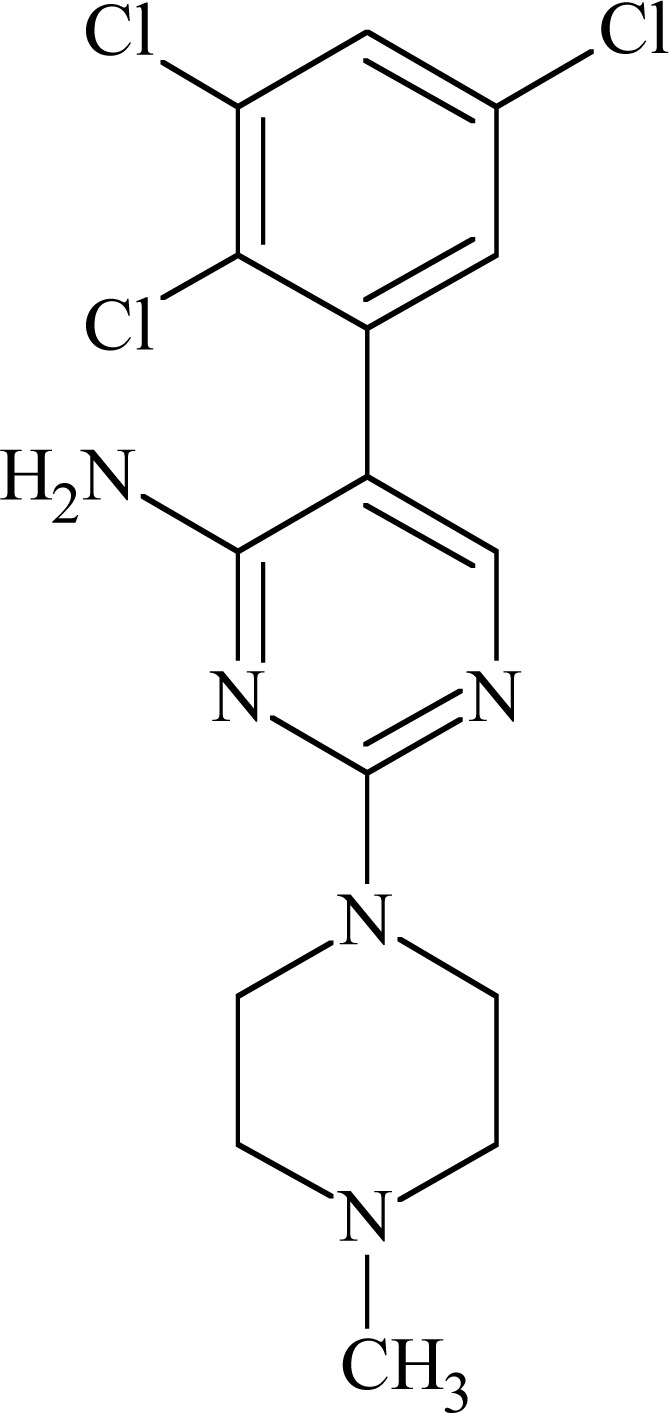	10.3	Prevention of neuronal toxicity following stroke	1764 – strong interaction;
			1771 – weak interaction;
			1760 – weak interaction;
			Possible interactions with amino acid
			Residues in other transmembrane segments

**Table 4.** Classification of Voltage Gated Potassium Channels [According to 74]

Other Name	Type of Potassium Channel	Human Gene	Other Name	Type of Potassium Channel	Human Gene
*Shaker*-related family	K_V_1.1	KCNA1	KvLQT	K_V_7.1	KCNQ1
	K_V_1.2	KCNA2	KQT2	K_V_7.2	KCNQ2
	K_V_1.3	KCNA3	-	K_V_7.3	KCNQ3
	K_V_1.4	KCNA4	-	K_V_7.4	KCNQ4
	K_V_1.5	KCNA5	-	K_V_7.5	KCNQ5
	K_V_1.6	KCNA6	K_V_2.3rc	K_V_8.1	KCNV1
	K_V_1.7	KCNA7	-	K_V_8.2	KCNV2
	K_V_1.8	KCNA10	Modifiers	K_V_9.1	KCNS1
*Shab*-related family	K_V_2.1	KCNB1		K_V_9.2	KCNS2
	K_V_2.2	KCNB2		K_V_9.3	KCNS3
*Shaw*-related family	K_V_3.1	KCNC1	eag1	K_V_10.1	KCNH1
	K_V_3.2	KCNC2	eag2	K_V_10.2	KCNH5
	K_V_3.3	KCNC3	erg1	K_V_11.1	KCNH2
	K_V_3.4	KCNC4	erg2	K_V_11.2	KCNH6
*Shal*-related family	K_V_4.1	KCND1	erg3	K_V_11.3	KCNH7
	K_V_4.2	KCND2	elk1, elk3	K_V_12.1	KCNH8
	K_V_4.3	KCND3	elk2	K_V_12.2	KCNH3
Modifier	K_V_5.1	KCNF1	elk1	K_V_12.3	KCNH4
Modifiers	K_V_6.1	KNCG1			
	K_V_6.2	KCNG2			
	K_V_6.3	KCNG3			
	K_V_6.4	KCNG4			

**Table 5.** Localization of KV7 Family Members in Humans [73, 74, 83]

Type of Potassium Channel α-subunit	Localization			Disease In Which Serves As Target
	CNS	Subcellular	Other	
K_V_7.1	-	-	Heart, kidney, rectum, lung, placenta, cochlea	Arhytmias
K_V_7.2	Brain, ganglia	Axons	Lung, testis, heart, eye, small intestine, breast	Epilepsy
K_V_7.3	Brain, ganglia	Axons, neuronal somata, dendrites	Testis, retina, colon, eye	Epilepsy
K_V_7.4	Brain		Cochlea, vestibular hair cells, placenta	Epilepsy Psychosis
K_V_7.5	Brain, ganglia	Neuronal somata, dendrites	Skeletal muscle	Epilepsy

**Table 6.** Types of Calcium Channels and their Antagonists [112, 118-119]

Family	Subfamily	Subtype/Type	Type	Antagonist
HVA	Ca_v_1	Ca_v_1.1	L	Nifedipine Amlodipine
		Ca_v_1.2	L	
		Ca_v_1.3	L	
		Ca_v_1.4	L	
	Ca_v_2	Ca_v_2.1	P/Q	-
		Ca_v_2.2	N	ω-conotoxin-MVIIA, ziconotide Pyrazolylpiperidine derivative
		Ca_v_2.3	R	Lamotrigine
LVA	Ca_v_3	Ca_v_3.1	T	Ethosuximide Mesuximide
		Ca_v_3.2	T	
		Ca_v_3.3	T	

**Table 7.** Nomancletures of ASICs

Subunit	Isoform	Synonym
ASIC1	ASIC1a	ASICα, BNaC2α
	ASIC1b	ASICα, BNaC2β
ASIC2	ASIC2a	MDEG1, BNaC1α, BNC1
	ASIC2b	MDEG2, BNaC1 β, BNC1
ASIC3		DRASIC, TNaC
ASIC4		SPASIC

Alternative nomenclature of ASICs: BNaC - Na channel in the brain; MDEG - mammalian degenerin; DRASIC - dorsal root ASIC; SPASIC, spinal cord ASIC; TNaC - testis [273, 263, 285].

**Table 8.** Disorders in CNS Involving ASICs

Disorder	ASIC Subunit			
	1	2	3	4
Anxiety [283; 286, 287]	+			
Autoimmune encephalitis [288]	+			
Depression [289]	+			
Epilepsy [290-292]	+	+		+
Glioma [293]	+	+		
Huntington’s disease [294]	+	+		
Multiple sclerosis [295, 288]	+			
Pain [272, 296-299]	+	+		

**Table 9.** Binding Affinities of Known ASIC Ligands

Ligand	Subunit	IC_50_

A-317567 [301]	ASIC1	2.0 µM
	ASIC2	29.1 µM
	ASIC3	9.5 µM

Amiloride [263, 301-302]	ASIC1a	10 µM
	ASIC1b	21 µM
	ASIC2a	0.15 µM
	ASIC3	37.2 µM
	ASIC3	63.0 µM

APETx2 (Sea anemone toxin) [303]	ASIC3	63.0 nM
	ASIC1a+3	2.0 µM
	ASIC1b+3	0.9 µM
	ASIC2b+3	117.0 nM

Acetylsalicylic acid [304]	ASIC3	260 µM

DAPI [305]	ASIC in hippocampal neurons	2.8 µM

Diclofenac [304]	ASIC3	92 µM

Diminazene [305]	ASIC in hippocampal neurons	0.3 µM

Flurbiprofen [304]	ASIC1a	350 µM

Gentamycin [306]	ASIC in dorsal root ganglion neurons	44 µM

Hydroxystilbamidine [305]	ASIC in hippocampal neurons	1.5 µM

Ibuprofen [304]	ASIC1a	350 µM

Nafamostat [307]	ASIC depending on the subunit	2-70 µM

Neomycin [306]	ASIC in dorsal root ganglion neurons	32 µM

PcTx-1 (Tarantula toxin, psalmotoxin 1) [308-310]	ASIC1a	0.9 nM

Pentamidine [305]	ASIC in hippocampal neurons	38 µM

Synthalin [305]	ASIC in hippocampal neurons	29 µM

**Table 10.** Classification of TRP Channels

	Type	Name	Subtype
Families	TRPA	Ancyrin	TRPA1
	TRPC	Canonical	TRPC1-7
	TRPM	Melastatin	TRP1-8
	TRPN	No mechanorecepor potential	
	TRPV	Vanilloid	TRP1-6
Subfamilies	TRPP	Policystin	TRPP1-3, 5
	TRPML	Mucolipin	TRPML1-3
